# Seven-transmembrane receptor protein RgsP and cell wall-binding protein RgsM promote unipolar growth in Rhizobiales

**DOI:** 10.1371/journal.pgen.1007594

**Published:** 2018-08-13

**Authors:** Simon Schäper, Hamish C. L. Yau, Elizaveta Krol, Dorota Skotnicka, Thomas Heimerl, Joe Gray, Volkhard Kaever, Lotte Søgaard-Andersen, Waldemar Vollmer, Anke Becker

**Affiliations:** 1 LOEWE Center for Synthetic Microbiology (SYNMIKRO), Philipps-Universität Marburg, Marburg, Germany; 2 Faculty of Biology, Philipps-Universität Marburg, Marburg, Germany; 3 Center for Bacterial Cell Biology, Institute for Cell and Molecular Biosciences, Newcastle University, Newcastle upon Tyne, United Kingdom; 4 Department of Ecophysiology, Max Planck Institute for Terrestrial Microbiology, Marburg, Germany; 5 Institute for Cell and Molecular Biosciences, Newcastle University, Newcastle upon Tyne, United Kingdom; 6 Research Core Unit Metabolomics, Hannover Medical School, Hannover, Germany; The University of North Carolina at Chapel Hill, UNITED STATES

## Abstract

Members of the Rhizobiales (class of α-proteobacteria) display zonal peptidoglycan cell wall growth at one cell pole, contrasting with the dispersed mode of cell wall growth along the sidewalls of many other rod-shaped bacteria. Here we show that the seven-transmembrane receptor (7TMR) protein RgsP (SMc00074), together with the putative membrane-anchored peptidoglycan metallopeptidase RgsM (SMc02432), have key roles in unipolar peptidoglycan formation during growth and at mid-cell during cell division in *Sinorhizobium meliloti*. RgsP is composed of a periplasmic globular 7TMR-DISMED2 domain, a membrane-spanning region, and cytoplasmic PAS, GGDEF and EAL domains. The EAL domain confers phosphodiesterase activity towards the second messenger cyclic di-GMP, a key regulatory player in the transition between bacterial lifestyles. RgsP and RgsM localize to sites of zonal cell wall synthesis at the new cell pole and cell divison site, suggesting a role in cell wall biogenesis. The two proteins are essential for cell wall biogenesis and cell growth. Cells depleted of RgsP or RgsM had an altered muropeptide composition and RgsM binds to peptidoglycan. RgsP and RgsM orthologs are functional when interchanged between α-rhizobial species pointing to a conserved mechanism for cell wall biogenesis/remodeling within the Rhizobiales. Overall, our findings suggest that RgsP and RgsM contribute to the regulation of unipolar cell wall biogenesis in α-rhizobia.

## Author summary

Bacteria face the challenge of maintaining their peptidoglycan cell wall integrity during growth and division. The enzymes involved in cell wall biogenesis are tightly regulated and targeting of these enzymes by β-lactams cause cell death. In contrast to the well-characterized mode of dispersed cell wall formation in many rod-shaped bacteria, the mechanisms controlling polar cell wall formation in α-proteobacteria are largely unknown. Seven-transmembrane receptors (7TMRs) are widespread in eukaryotes and prokaryotes but only few of them have been functionally studied in bacteria. Here we suggest that a 7TMR-DISM protein cooperates with a putative peptidoglycan-hydrolyzing protein, to facilitate unipolar cell wall formation and cell division in the Rhizobiales. This 7TMR-DISM protein also contributes to degradation of the second messenger cyclic di-GMP.

## Introduction

Rod-shaped bacteria have evolved diverse modes of cell growth. In *Bacillus subtilis*, *Escherichia coli* and *Caulobacter crescentus*, cells grow by elongating along the lateral sidewall, incorporating peptidoglycan (PG) cell wall material in a dispersed pattern along the sidewall [1]. Other bacteria elongate by zonal growth in which PG is incorporated at one or both cell poles [2]. Unipolar growth is observed in α-proteobacterial Rhizobiales, such as *Agrobacterium tumefaciens* and *Sinorhizobium meliloti* [3], while Gram-positive Actinomycetales including *Mycobacterium tuberculosis* grow bipolarly [4]. Later in the cell cycle, all species switch PG synthesis completely or partially to mid-cell for cell division [1]. During PG synthesis nascent material is incorporated into the pre-existing PG structure (sacculus), by the activity of hydrolases which selectively cleave bonds in the stress-bearing sacculus [5]. Because the integrity of the PG sacculus is essential for maintaining cell shape and resisting turgor [6], cell growth and PG synthesis require precise spatial and temporal regulation of the incorporation of new material into the PG sacculus [7]. Rod-shaped bacteria with a dispersed mode of PG incorporation along the lateral cell wall utilize the cytoplasmic actin-like MreB protein to direct PG synthesis. Dynamic filaments or patches of MreB are believed to serve as platforms for the intracellular and extracellular PG synthesis machineries [8,9]. By contrast, most rod-shaped bacteria with polar growth do not contain MreB homologs [10] and it is currently unknown how polar cell wall elongation is regulated in these bacteria.

Bis-(3′-5′)-cyclic dimeric guanosine monophosphate (c-di-GMP) has a central role in the regulation of motility, adherence, biofilm formation and virulence and in several bacterial species, also for promoting cell cycle progression, growth and development [11–15]. c-di-GMP is synthesized by diguanylate cyclases (DGCs) with a conserved GGDEF domain and degraded by phosphodiesterases (PDEs) with either an EAL domain or a HD-GYP domain [16]. In the α-proteobacterium *C*. *crescentus*, the spatial organization of proteins involved in c-di-GMP metabolism contributes to cell polarity and cell cycle progression [14], and we showed previously that strong overproduction of this second messenger inhibited growth and resulted in cell filamentation in *S*. *meliloti* [17].

Seven-transmembrane receptors (7TMRs) form the largest, most ubiquitous and most versatile family of membrane receptors. In eukaryotes, they are involved in signaling via interaction with cytoplasmic G-proteins [18]. A distinct class of bacterial 7TMRs consists of so-called 7TMR-DISMs, which stands for 7TMR with diverse intracellular signaling modules [19]. 7TMR-DISM proteins contain seven transmembrane α-helices (7TMR-DISM_7TM domain) fused to various cytoplasmic signaling and/or extracytoplasmic 7TMR-DISMED1 or 7TMR-DISMED2 domains [19]. These extracytoplasmic domains have been predicted to bind ligands such as carbohydrates [19]. Common cytoplasmic signaling modules in 7TMR-DISM proteins include histidine protein kinase domains, GGDEF and EAL domains involved in c-di-GMP homeostasis and sensory Per-Arnt-Sim (PAS) domains. PAS domains are known to sense small molecules, ions, gases, light or redox state [20]. With up to 14 paralogs per genome, for example in the spirochete *Leptospira interrogans*, 7TMR-DISMs are widely distributed in both Gram-negative and Gram-positive bacteria [19]. However, only a few of these proteins have been functionally characterized. These include the *Pseudomonas aeruginosa* 7TMR-DISM histidine kinases RetS and LadS, which are involved in regulation of biofilm formation, and the GGDEF domain containing protein NicD, which is involved in biofilm dispersal [21,22].

The 7TMR-DISM protein SMc00074 (renamed RgsP for rhizobial growth and septation c-di-GMP phosphodiesterase) was previously shown to be essential in *S*. *meliloti* [17,23,24]. Here, we provide evidence that RgsP is important for PG synthesis in certain α-rhizobial species. We identify SMc02432 (a putative membrane-anchored periplasmic PG metallopeptidase, renamed RgsM for rhizobial growth and septation metallopeptidase) as a RgsP interaction partner and show that both are required for unipolar cell growth in *S*. *meliloti* and related Rhizobiales. RgsP was also found to be an active PDE, substantially contributing to c-di-GMP homeostasis and hence possibly connecting c-di-GMP signaling with spatiotemporal control of PG synthesis.

## Results

### RgsP is required for normal growth and cell morphology

RgsP is composed of 7TMR-DISMED2, 7TMR-DISM_7TM, PAS, GGDEF and EAL domains. Previous systematic mutagenesis of c-di-GMP-related genes identified *rgsP* (*SMc00074*) as a potentially essential gene in *S*. *meliloti* Rm2011 [17]. C-terminally 3×FLAG-tagged RgsP accumulated in growing cells and was only detected at very low levels in stationary phase cells (S1A Fig), suggesting a role during cell growth. To further study the function of this protein, we constructed a RgsP depletion strain (Rm2011 *rgsP*^*dpl*^) by placing the native *rgsP* gene under the control of an IPTG-inducible promoter. Using the same promoter, we confirmed depletion of a C-terminally 3×FLAG-tagged RgsP variant in the absence of IPTG (S1B Fig).

**Fig 1:**
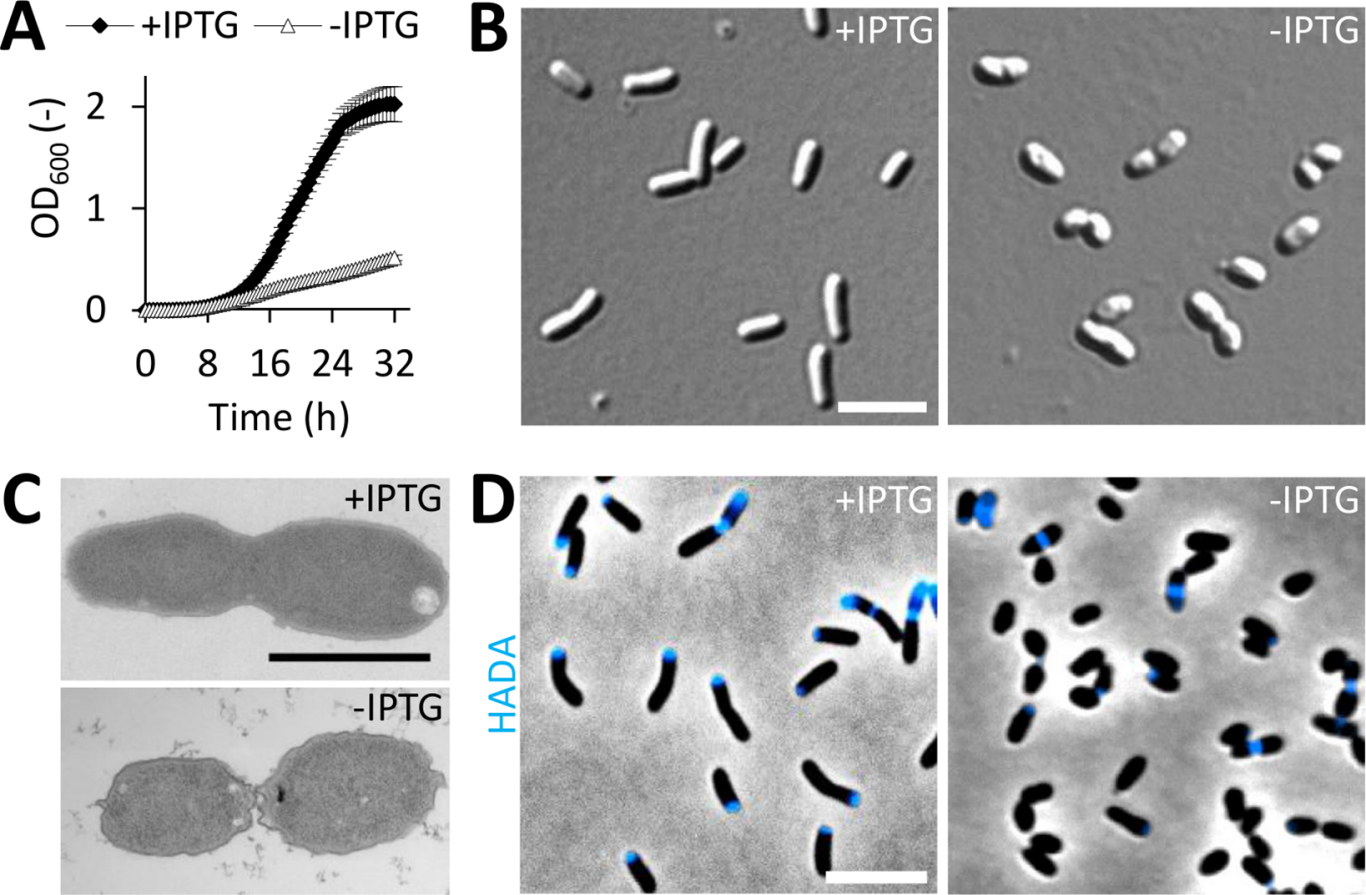
RgsP function is related to cell growth and division. (A) Growth of *S*. *meliloti* RgsP depletion strain Rm2011 *rgsP*^*dpl*^ in TY medium in presence or absence of IPTG. OD_600_ was recorded every 30 min. Error bars represent the standard deviation of six biological replicates. (B-D) Microscopy analysis of Rm2011 *rgsP*^*dpl*^, grown in TY medium in presence or absence of IPTG for 24 h. (B) DIC microscopy images. (C) Transmission electron microscopy images. (D) Phase contrast and fluorescence microscopy images of cells pulse-labeled with HADA for 3 min. (B,D) Bar, 5 μm. (C) Bar, 1 μm.

Growth of Rm2011 *rgsP*^*dpl*^ was strongly dependent on IPTG (Fig 1A). Cells cultured in the presence of IPTG showed wild type-like growth and morphology, whereas RgsP-depleted cells lost the rod shape and had an irregular, uneven cell surface (Fig 1B and 1C; S2 and S3A Figs). 4.7% of these cells were stained by the dead cell indicator propidium iodide (S4A Fig). This suggests that the physiological effect of RgsP depletion probably is mostly bacteriostatic after 24 h of incubation in absence of IPTG. To estimate the capability of RgsP-depleted cells to resume growth, we analyzed colony formation and microscopically monitored growth on TY medium containing IPTG. In cultures of Rm2011 *rgsP*^*dpl*^ grown without added IPTG for 12 or 24 h, the number of colony forming units (CFU ml^-1^ OD_600_^-1^) was reduced by 67.8% or 97.4%, respectively, compared to IPTG-supplemented cultures (S4B Fig). Following the fate of RgsP-depleted cells (previously grown in the absence of IPTG for 24 h), 25.8% were able to recover and to give rise to microcolonies on TY agarose pads with IPTG. In contrast, only 3.0% of the cells were able to divide at least once during the 12 h observation period on pads lacking IPTG (S4C Fig). Thus, RgsP depletion predominantly resulted in growth arrest, which was relieved upon IPTG addition in a minor fraction of the cells only. However, when interpreting these results, it has to be taken into account that the cells grown under depletion conditions may contain some residual amounts of RgsP.

Zones of PG synthesis were visualized by HADA (7-hydroxycoumarin-3-carboxylic acid-D-alanine)-labeling [25]. In IPTG-supplemented cultures with induced *rgsP* expression, 98% of the cells were stained at one of the cell poles and/or septal region. By contrast, only 33% of the RgsP-depleted cells incorporated HADA (Fig 1D; S5A Fig). Moreover, upon RgsP depletion, the proportion of pre-divisional cell doublets with visible septum constriction increased to 42% compared to 13% in RgsP-replete cultures (Fig 1D; S5B Fig). These results suggest that polar cell wall synthesis and late stages of cell division were impaired in the absence of RgsP.

### RgsP localizes dynamically during the cell cycle

**Fig 2:**
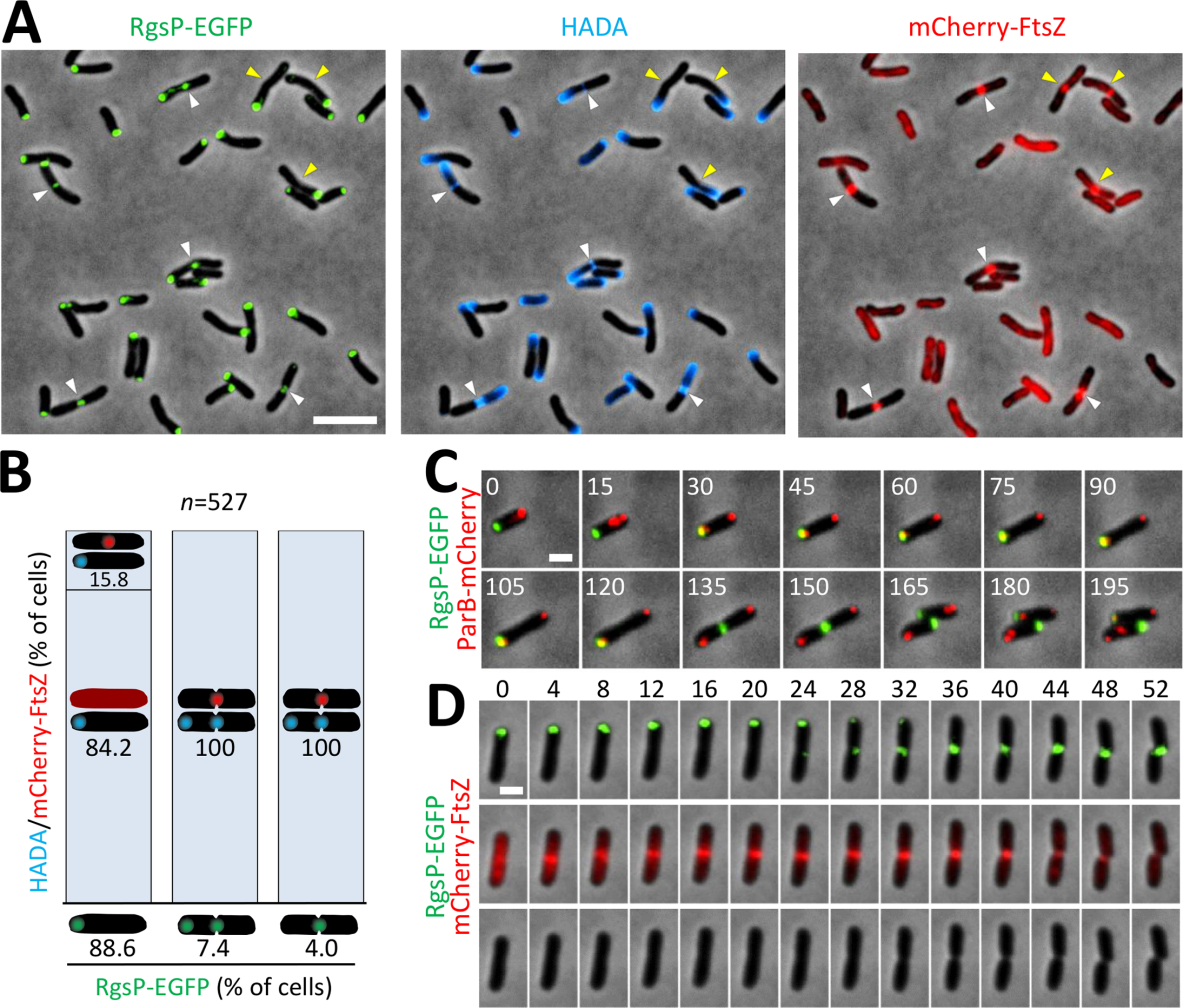
RgsP localizes to sites of zonal cell wall synthesis. (A) Fluorescence microscopy images of exponentially growing Rm2011 *rgsP-egfp* (gene fusion at the native genomic location) harboring pSRKGm-*mCherry-ftsZ*. Cells were pulse-labeled with HADA for 3 min. Cells with a mCherry-FtsZ focus (yellow arrowheads) and additional RgsP-EGFP and HADA signals at mid-cell (white arrowheads) are indicated. Bar, 5 μm. (B) Quantitative analysis of localization of HADA-stained regions of zonal cell wall synthesis and mCherry-FtsZ relative to localization of RgsP-EGFP in exponentially growing cells. The population was first divided into three classes dependent on EGFP signal localization and each class was evaluated for location of mCherry-FtsZ and HADA signals. *n*, total number of cells considered for statistical analysis. (C,D) Time-lapse microscopy images of Rm2011 *rgsP-egfp* harboring either pSRKGm-*parB*-*mCherry* (C) or pSRKGm-*mCherry-ftsZ* (D). The doubling time of analyzed bacteria was approximately 135 min (C). Time is given in minutes. Bar, 1 μm. Signals: HADA, blue; EGFP, green; mCherry, red.

To determine the subcellular localization of RgsP, we replaced *rgsP* with *rgsP-egfp* at its native genomic location. In exponentially growing *S*. *meliloti* cells, RgsP-EGFP co-localized with HADA-labeled sites of PG synthesis at one of the cell poles and the division site (Fig 2A and 2B). Time-lapse microscopy showed that RgsP-EGFP remained at the pole during the entire phase of cell elongation until it relocated to the mid-cell region (Fig 2C). We next localized RgsP-EGFP simultaneously with ParB-mCherry. ParB binds to the *parS* sites close to the chromosomal origin of replication [26]. Early in the cell cycle, a single fluorescent ParB-mCherry focus localized at the old cell pole and RgsP-EGFP localized at the opposite, new pole (Fig 2C). Later, a second ParB-mCherry focus moved to the new pole. Following a period of co-localization (~90 minutes) of RgsP-EGFP and ParB-mCherry at the new pole, the polar RgsP-EGFP signal disappeared and appeared at mid-cell. Thus, RgsP-EGFP localizes at the new cell pole early in the cell cycle and later at the cell division site. Importantly, these are the sites of PG synthesis.

To relate the temporal pattern of RgsP relocation to the mid-cell to known pre-divisional processes, we co-localized RgsP with the early divisome component FtsZ [7]. mCherry-FtsZ showed diffuse fluorescence in growing cells, and localized to the mid-cell in pre-divisional cells (Fig 2A). Septal localization of RgsP-EGFP always coincided with the presence of a mCherry-FtsZ focus at mid-cell, but not *vice versa* (Fig 2A and 2B), suggesting that RgsP accumulates at the septal site later than FtsZ. Time-lapse microscopy showed localization of RgsP-EGFP at mid-cell about 24 minutes after occurrence of the mCherry-FtsZ focus. This RgsP-EGFP relocalization was immediately followed by the onset of septum constriction, which was completed by cell division about 24 minutes later (Fig 2D). This implies that RgsP may also have a function in septation.

The dynamics of RgsP relocation to the mid-cell region was analyzed at a higher time resolution relative to PleD. We found previously that the DGC PleD localizes to the new cell pole within 20 minutes before cell division [17]. Simultaneous tracking of PleD-EGFP and RgsP-mCherry showed that the accumulation of PleD-EGFP at the growing cell pole temporally correlated with the RgsP-mCherry signal fading away at the pole and relocating to the division site (S6 Fig), indicating mutually exclusive localization of RgsP and PleD at the new pole.

### The N-terminal domains of RgsP are essential

**Fig 3:**
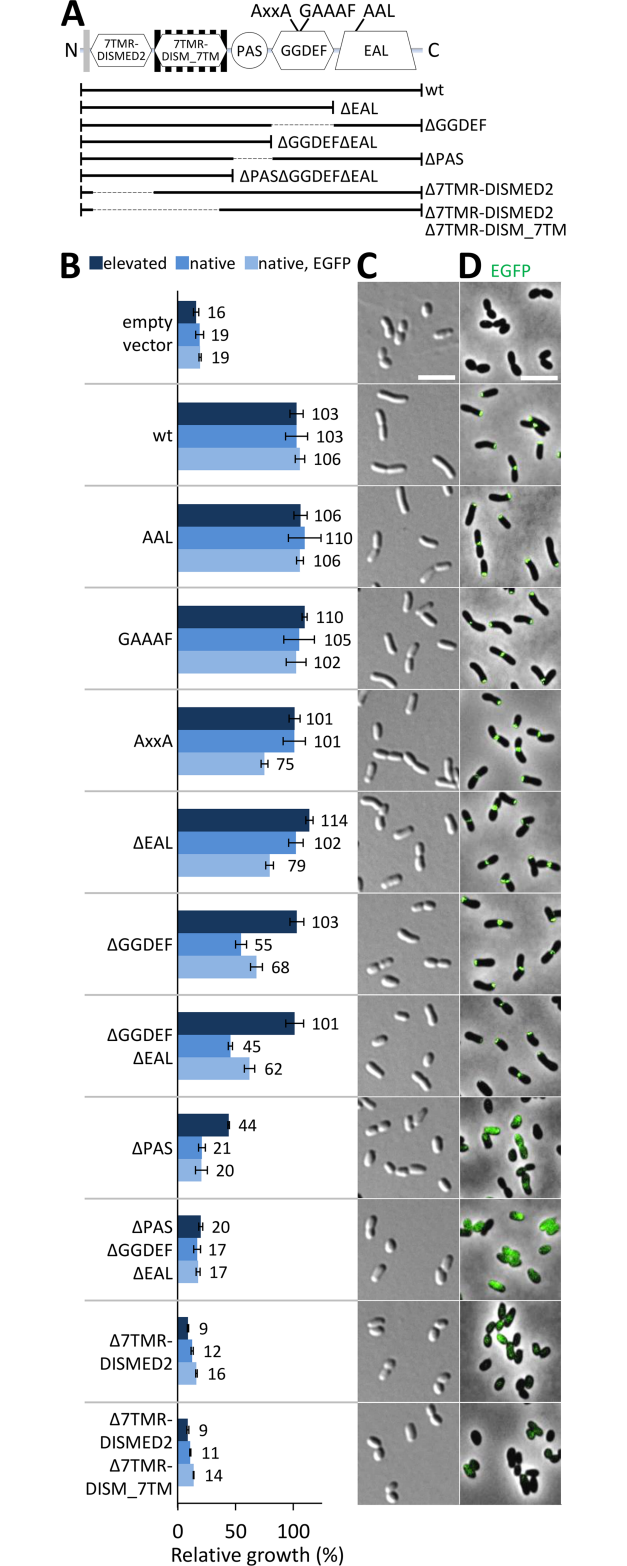
RgsP essentiality resides in its N-terminal part. (A) RgsP domain architecture and generated mutant variants. (B) Complementation of the RgsP depletion strain Rm2011 *rgsP*^*dpl*^ growth defect by *rgsP* variants expressed from *P*_*syn*_ (elevated), *rgsP* variants expressed from P*_rgsP_ (native) or *egfp*-tagged *rgsP* variants expressed from P*_rgsP_ (native, EGFP). Relative growth was calculated as a ratio of OD_600_ of cultures without IPTG (depleted of RgsP produced from the chromosome) and OD_600_ of cultures with IPTG (non-depleted of RgsP produced from the chromosome), 24 h after inoculation. Error bars represent the standard deviation of three biological replicates. The absolute OD_600_ values are shown in S8A Fig. (C,D) Microscopy images of Rm2011 *rgsP*^*dpl*^, ectopically expressing the indicated (C) non-tagged and (D) *egfp*-tagged *rgsP* variants, acquired after 24 h of growth in TY medium without IPTG. Bars, 5 μm.

To dissect the functionality of RgsP relative to its complex domain organization, we generated RgsP variants, each with or without C-terminally fused EGFP (Fig 3A). The gene variants were ectopically expressed either from the native promoter P*_rgsP_ (S7A Fig) on the single-copy plasmid pABCS2-mob (native level of 3×FLAG-tagged RgsP_wt_ (S1C Fig)) or from a constitutive synthetic promoter (*P*_*syn*_) on the low-copy plasmid pR_*egfp* (elevated level of 3×FLAG-tagged RgsP_wt_ (S1D Fig)) in the RgsP-depleted strain Rm2011 *rgsP*^*dpl*^. All RgsP variants were assayed for their ability to complement the cell growth and morphology defects of the RgsP-depleted strain, and for subcellular localization of EGFP fusion proteins. Ectopically expressed *rgsP*_wt_ or *rgsP*_wt_*-egfp* restored growth and cell morphology of RgsP-depleted cells (Fig 3B and 3C), and RgsP_wt_-EGFP localized similarly when expressed from the ectopic and native gene locus (Figs 2 and 3D).

Point mutations targeting the conserved inhibitory site (I site) and the DGC active site motifs in the GGDEF domain (RxxD to AxxA and GGDQF to GAAAF) and the PDE active site in the EAL domain (EAL to AAL), or removal of the EAL domain did not significantly affect protein localization or complementation of the cell growth and morphology defects (Fig 3B, 3C and 3D). RgsP and RgsP-EGFP variants lacking the GGDEF or both GGDEF and EAL domains were unable to fully complement growth and morphology defects unless they were expressed at higher levels from *P*_*syn*_ (Fig 3B and 3C). Nevertheless, the EGFP-tagged versions showed normal cellular localization (Fig 3D). When gene expression was driven by P*_rgsP_ on pABCS2-mob, levels of corresponding 3×FLAG-tagged wild type protein and variants were similar, except for RgsP_ΔGGDEF_, which was detected at 67% of the corresponding wild type protein level (S1C Fig). This implies that RgsP lacking the EAL domain fully supports cell growth, whereas lack of the GGDEF domain impairs protein stability or function.

To investigate the possible role of c-di-GMP in RgsP function, we tested complementation of RgsP depletion by *rgsP* and *rgsP-egfp* variants in the c-di-GMP-deficient strain Rm2011 ΔXVI, which lacks all genes predicted to encode active DGCs and in which the level of c-di-GMP was below the detection limit [17]. The complementation properties of each of these RgsP versions were indistinguishable in strains with or without c-di-GMP, and RgsP-EGFP showed normal localization in Rm2011 ΔXVI (S8 Fig). This suggests that c-di-GMP is not required for RgsP localization and the essential function of this protein in cell growth.

Deletion of the N-terminal domains 7TMR-DISMED2, 7TMR-DISM_7TM or PAS abolished the ability of RgsP to complement RgsP depletion phenotypes, irrespective of the expression vector (Fig 3B and 3C). When gene expression was driven by P*_rgsP_ on pABCS2-mob (or *P*_*syn*_ on pR_*egfp*), 3×FLAG-tagged RgsP_ΔPAS_, RgsP_ΔPASΔGGDEFΔEAL_, RgsP_Δ7TMR-DISMED2_, and RgsP_Δ7TMR-DISMED2Δ7TMR-DISM_7TM_ variants accumulated to 58% (736%), 30% (592%), 6% (236%) and 9% (19%), respectively, of the corresponding wild type protein levels (S1C and S1D Fig). Furthermore, the fluorescence signal of these RgsP variants, tagged with EGFP, did not show the characteristic polar and septal localization (Fig 3D).

Taken together, these data suggest that the N-terminal part of RgsP determines the essentiality of this protein, whereas the EAL domain is dispensable for the growth-promoting function of RgsP.

### RgsP is an active c-di-GMP phosphodiesterase

Since the GGDEF and EAL domains may have a regulatory role, we analyzed the enzymatic activities and ability to bind c-di-GMP *in vitro*. A purified His_6_-tagged variant of RgsP containing the PAS, GGDEF and EAL domains (His_6_-RgsP_PAS-GGDEF-EAL_) hydrolyzed [α-^32^P]-c-di-GMP, whereas no cleavage product was detected with the PDE active site mutant variant His_6_-RgsP_PAS-GGDEF-EAL_-AAL (S9A Fig). In a DGC activity assay with [α-^32^P]-GTP as a substrate, His_6_-RgsP_PAS-GGDEF-EAL_-AAL did not synthesize c-di-GMP, in contrast to *C*. *crescentus* DgcA used as a positive control (S9B Fig). This is in agreement with the degenerate active site GGDQF in the RgsP GGDEF domain and our previous *in vitro* DGC activity assay with a RgsP fragment comprising the PAS, GGDEF and EAL (active site intact) domains [17]. Since an intact I site RxxD is present in the GGDEF domain of RgsP, we assayed for the ability of RgsP to bind c-di-GMP in a differential radial capillary action of ligand assay (DRaCALA) using a His_6_-tagged RgsP variant containing only the PAS and GGDEF domains (His_6_-RgsP_PAS-GGDEF_). In this assay, the positive control His_6_-DmxB from *Myxococcus xanthus* produced the characteristic DRaCALA pattern, whereas His_6_-RgsP_PAS-GGDEF_ was not able to prevent the diffusion of [α-^32^P]-c-di-GMP (S9C Fig). Thus, RgsP_PAS-GGDEF-EAL_ has c-di-GMP PDE activity, presumably catalysed by the EAL domain, but the GGDEF domain does not bind or synthesize c-di-GMP.

To evaluate c-di-GMP PDE activity of RgsP *in vivo*, we determined the c-di-GMP content of RgsP-depleted Rm2011 *rgsP*^*dpl*^ complemented with *rgsP*_wt_, *rgsP*_AAL_ and *rgsP*_GAAAF_ expressed from P*_rgsP_. The c-di-GMP content of cells expressing *rgsP*_wt_ and *rgsP*_GAAAF_ was similar, whereas expression of the PDE active site mutant variant *rgsP*_AAL_ resulted in a two-fold increase in c-di-GMP content (S9D Fig). This data is consistent with the *in vitro* data and provides evidence that RgsP substantially contributes to c-di-GMP degradation *in vivo*.

### RgsP interacts with the putative periplasmic metallopeptidase RgsM

To identify protein interaction partners of RgsP, we performed co-immunoprecipitation (Co-IP) experiments with a C-terminally 3×FLAG-tagged variant of RgsP, encoded by *rgsP*-*3×flag* replacing the native *rgsP* at its chromosomal location. 27 RgsP interaction partner candidates were identified (S1 Table). Among these, the hypothetical transmembrane protein RgsM (SMc02432) was most abundant and identified with the highest number of unique peptides. Co-IP with RgsM-3×FLAG resulted in identification of RgsP, further supporting an interaction between the two proteins (S2 Table).

**Fig 4:**
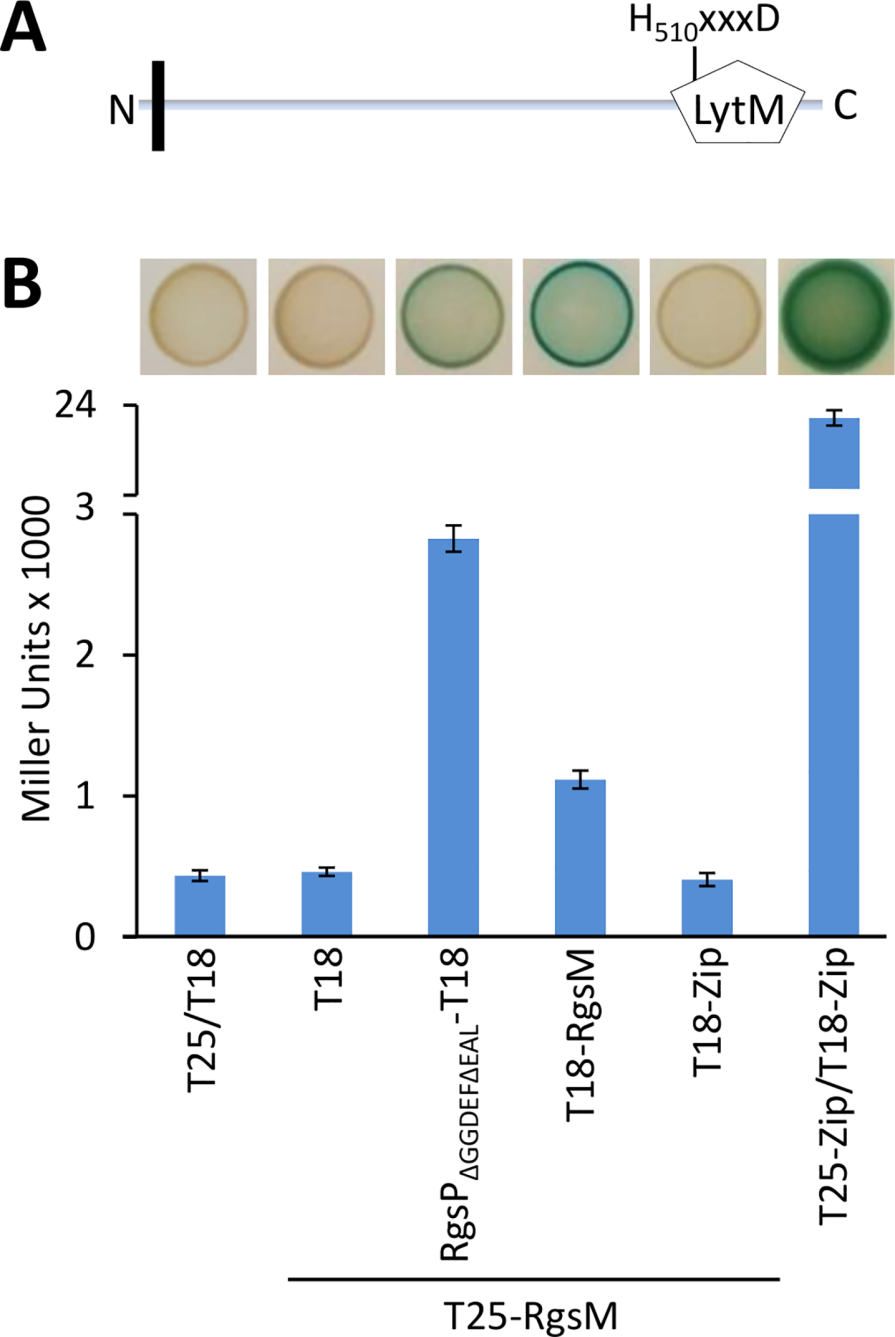
RgsP interacts with RgsM in bacterial two-hybrid system. (A) Schematic representation of the putative periplasmic metallopeptidase RgsM containing a transmembrane segment at the N-terminus and a C-terminal LytM domain. (B) Quantitative analysis of the interaction between RgsP and RgsM. β-galactosidase activity of *E*. *coli* BTH101, co-transformed with plasmids carrying *rgsP*_∆GGDEF∆EAL_ and full-length *rgsM* translationally fused to T25 and T18 fragments of *Bordetella pertussis* adenylate cyclase. The combination of yeast GCN4 leucin-zipper region (Zip) served as positive control. Error bars represent the standard deviation of three biological replicates. Blue-staining of colonies grown on a single LB agar plate containing X-Gal and IPTG is shown above.

Analysis of RgsM amino acid sequence with transmembrane topology and signal peptide prediction tool PHOBIUS [27] suggested cytoplasmic localization of the amino acids 1–31, a short hydrophobic transmembrane α-helical region (amino acids 32–57) and periplasmic localization of the remaining C-terminal portion (Fig 4A). Amino acids 508–606 of RgsM represent a conserved peptidase M23 domain (pfam01551) often referred to as a LytM domain. This domain is predicted to have PG endopeptidase activity and is characteristic for zinc-dependent metallopeptidases. The LytM domain of RgsM contains a conserved HxxxD motif (S3 Table), which is required for zinc ion coordination and hydrolysis of glycine-glycine bonds in the peptides of staphylococcal PG by *Staphylococcus aureus* LytM [28–30]. The remaining RgsM amino acid sequence did not provide any hint about its possible function. To verify the predicted membrane topology of RgsM, we fused this protein to a truncated *E*. *coli* alkaline phosphatase PhoA, which is only active in the periplasm, and is missing its own signal peptide. The phosphatase detection assay revealed that PhoA, following RgsM_1-66_, indeed localized to the periplasm in both *S*. *meliloti* and *E*. *coli*. This strongly suggests the periplasmic localization of RgsM_60-646_ (S10 Fig).

Interaction between RgsP and RgsM was verified by a bacterial two-hybrid assay [31]. In this assay, putative interaction partners, fused to *Bordetella pertussis* adenylate cyclase fragments T18 and T25, are produced in an *E*. *coli* adenylate cyclase-deficient strain. Interaction between the two fusion proteins results in reconstitution of a functional enzyme, which activates expression of the *lacZ* reporter gene. Simultaneous production of T18-RgsM and RgsP_∆GGDEF∆EAL_-T25 fusion proteins, as well as of T18-RgsM and T25-RgsM, resulted in increased β-galactosidase activity, indicating protein-protein interactions (Fig 4B). Thus, RgsM was able to interact with RgsP and to homodimerize in a heterologous host. This is consistent with the Co-IP data that suggested RgsP-RgsM interaction in *S*. *meliloti*.

### RgsM is essential and co-localizes with RgsP

**Fig 5:**
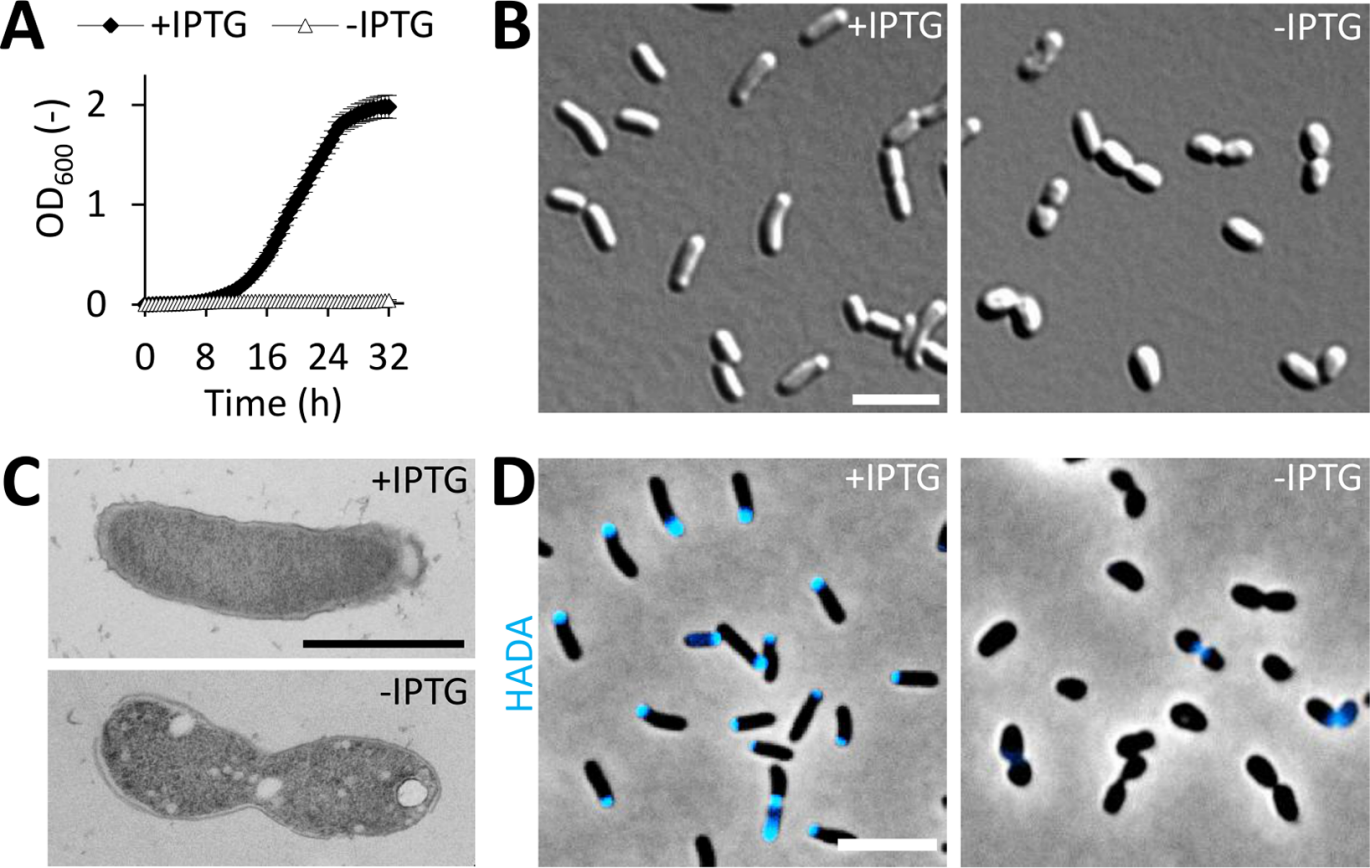
RgsM function is related to cell growth and division. (A) Growth of *S*. *meliloti* RgsM depletion strain Rm2011 *rgsM*^*dpl*^ in TY medium with or without added IPTG. OD_600_ was recorded every 30 min. Error bars represent the standard deviation of six biological replicates. (B-D) Microscopy analysis of Rm2011 *rgsM*^*dpl*^, grown in TY medium in presence or absence of IPTG for 24 h. (B) DIC microscopy images. (C) Transmission electron microscopy images. Bar, 1 μm. (D) Phase contrast and fluorescence microscopy images obtained after 3 min HADA labeling pulse. (B,D) Bar, 5 μm.

Attempts to generate a *rgsM* knockout mutant failed, suggesting that *rgsM* is essential. Similar to the growth phase-dependent accumulation of RgsP, C-terminally 3×FLAG-tagged RgsM was only detected in growing cells but not in stationary phase cells (S1A Fig). Next, we constructed a RgsM depletion strain Rm2011 *rgsM*^*dpl*^ by placing the chromosomal *rgsM* gene under the control of an IPTG-inducible promoter. Depletion of a C-terminally 3×FLAG-tagged RgsM variant was confirmed using the same genetic setup (S1B Fig). The growth of Rm2011 *rgsM*^*dpl*^ was significantly impaired in the absence of IPTG (Fig 5A). Strikingly similar to cells depleted of RgsP, RgsM-depleted cells lost the wild type rod shape (Figs 5B and 1B; S2 Fig). Electron microscopy revealed regions of low electron density in RgsM-depleted cells (Fig 5C; S3B Fig). 7.1% of these cells were stained by propidium iodide, indicating both lethal and bacteriostatic effects of RgsM depletion, similar to RgsP depletion (S4A Fig). Following depletion of RgsM for 12 or 24 h, the number of colony forming units was reduced by 89.4% or 99.5%, respectively, relative to cultures pre-incubated under non-depletion conditions (S4B Fig). We microscopically monitored growth of cells, previously grown under depletion conditions for 24 h. 10.9% of the previously RgsM-depleted cells gave rise to microcolonies on medium supplemented with IPTG, whereas 99.1% of these cells did not divide on medium lacking IPTG during the 12 h observation period (S4C Fig).

Labeling of RgsM-depleted cells with HADA revealed incorporation of new PG in only a minor part of pre-divisional cells at the septal region, whereas Rm2011 *rgsM*^*dpl*^ grown in IPTG-containing medium displayed a wild type-like HADA staining (Fig 5D; S5A Fig). Similar to RgsP depletion, RgsM depletion resulted in an increase in the proportion of pre-divisional cell doublets (with visible septum constriction) to 40% (S5B Fig). Thus, like RgsP, RgsM is required for cell growth and division and PG biosynthesis.

**Fig 6:**
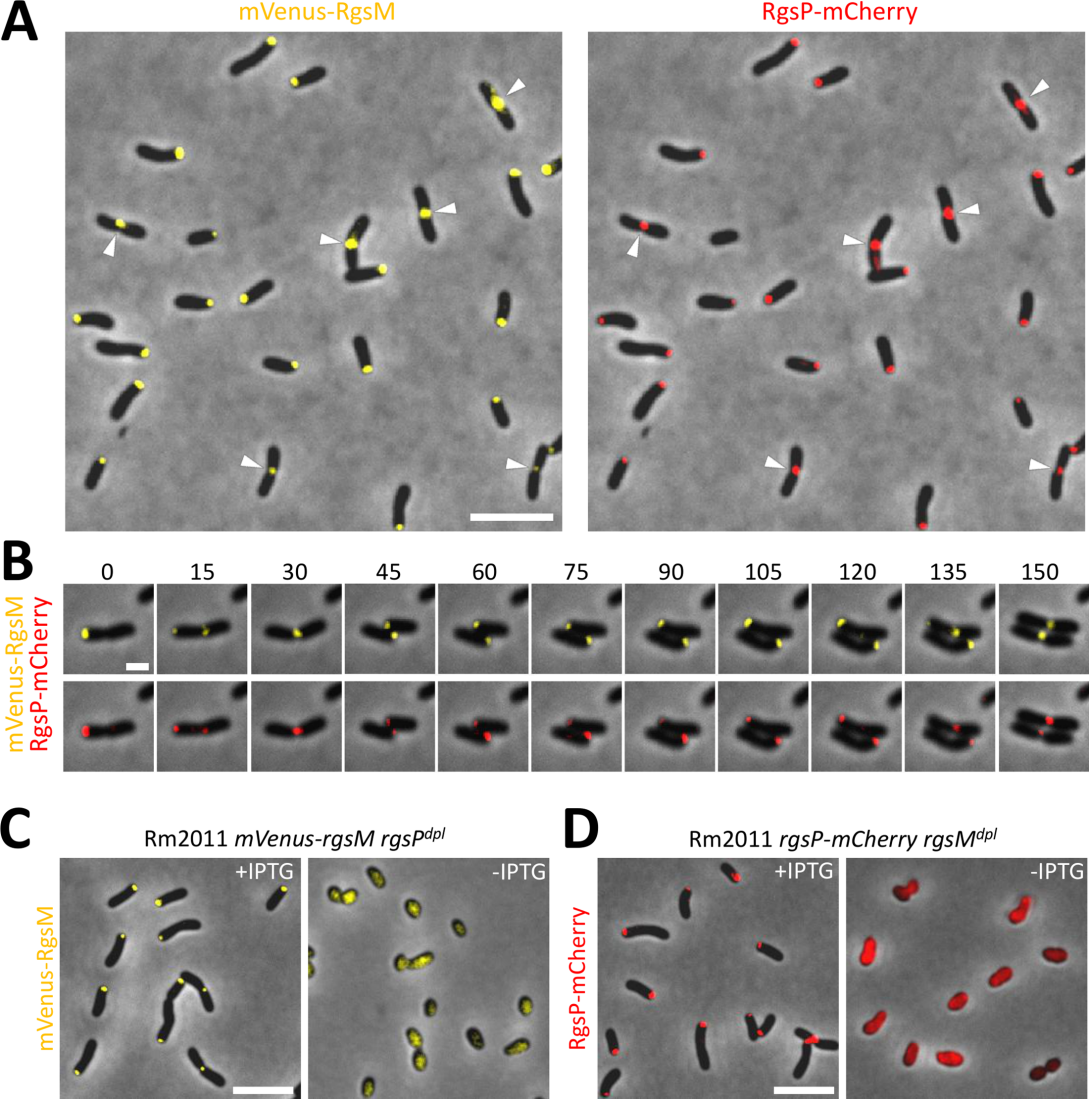
Localization of RgsP and RgsM throughout the cell cycle is concurrent and mutually interdependent. (A) Fluorescence microscopy images of exponentially growing Rm2011 *mVenus-rgsM rgsP-mCherry* (both gene fusions at the native genomic location). Arrowheads indicate the mid-cell region of pre-divisional cells. Bar, 5 μm. (B) Time-lapse microscopy images of cells described in panel A. The doubling time of analyzed bacteria was approximately 150 min. Time is shown in minutes. Bar, 1 μm. (C,D) Fluorescence microscopy images of RgsP depletion strain carrying *mVenus-rgsM* (gene fusion at the native genomic location) (C) and RgsM depletion strain carrying *rgsP-mCherry* (gene fusion at the native genomic location) (D) acquired after 24 h of growth in TY medium with or without IPTG. Bars, 5 μm.

To analyze RgsM localization relative to RgsP, we constructed a strain with *rgsP-mCherry* and *mVenus-rgsM* replacing *rgsP* and *rgsM* at their native genomic locations. mVenus-RgsM accumulated at one cell pole or at mid-cell and showed co-localization with RgsP-mCherry throughout the cell cycle (Fig 6A and 6B). This indicates that RgsM, like RgsP, localizes to sites of zonal cell wall synthesis. Localization of mVenus-RgsM was dependent on RgsP, since RgsP depletion resulted in diffuse mVenus-RgsM fluorescence (Fig 6C). Likewise, in RgsM-depleted cells, only diffuse RgsP-mCherry signal was observed (Fig 6D), indicating that polar and septal localization of RgsP and RgsM is mutually dependent. Taken together, these data imply a functional relation between RgsP and RgsM.

### Overexpression of *rgsM* compromises cell wall integrity

**Fig 7:**
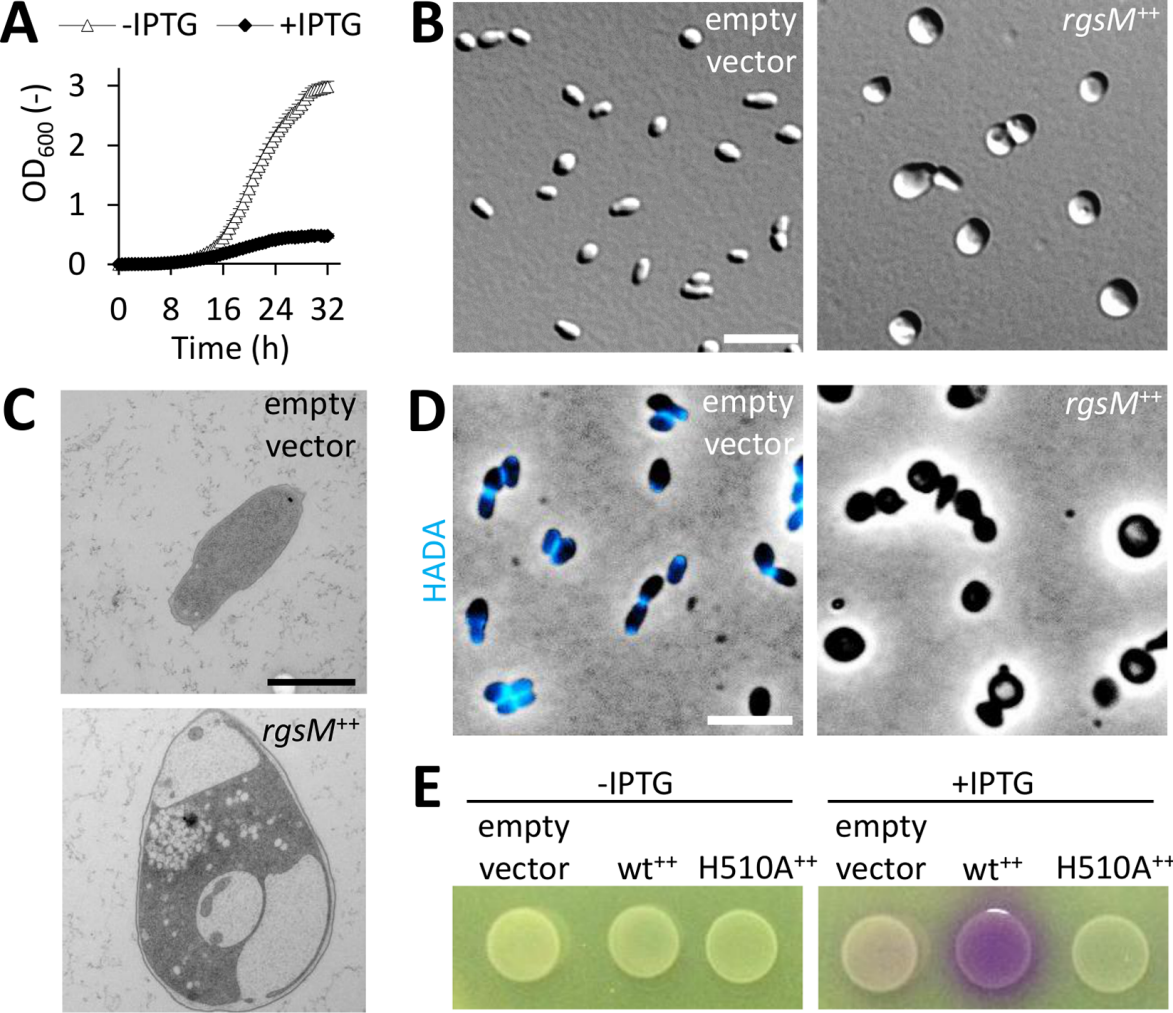
*rgsM* overexpression results in growth inhibition, altered cell morphology and cell envelope defects. (A) Growth of Rm2011 harboring *rgsM* overexpression plasmid pWBT-*rgsM* in LB medium with or without added IPTG. OD_600_ was recorded every 30 min. Error bars represent the standard deviation of six biological replicates. (B-D) Microscopy analysis of Rm2011, harboring either empty vector pWBT or pWBT-*rgsM* (*rgsM*^++^), grown in LB medium in presence of IPTG for 24 h. (B) DIC microscopy images. Bar, 5 μm. (C) Transmission electron microscopy images. Bar, 1 μm. (D) Phase contrast and fluorescence microscopy images of cells, pulse-labeled with HADA for 3 min. Bar, 5 μm. (E) CPRG staining of *E*. *coli* S17-1, harboring the empty vector pWBT, pWBT-*rgsM* (wt^++^) or pWBT-*rgsM*_H510A_ (H510A^++^) on LB agar lacking NaCl, with or without added 0.02 mM IPTG.

To further characterize the role of RgsM, we analyzed effects of *rgsM* overexpression. When grown in TY medium, RgsM-overproducing cells were indistinguishable from the empty vector control (S11 Fig). Contrastingly, in LB medium they grew very poorly and appeared enlarged and spherical (Fig 7A and 7B), with dramatically enlarged periplasm and inner membrane invaginations (Fig 7C; S3C Fig). Overexpression of *rgsM* in LB impaired PG biosynthesis, as judged from a very weak dispersed HADA staining, only visible after adjusting HADA fluorescence signal intensity, in contrast to cells carrying an empty vector, which showed polar and septal HADA signals (Fig 7D; S5A and S11C Figs). Noteworthy, even in the absence of RgsM overexpression, the rod cell shape differed slightly in TY and LB, with broader and shorter cells grown in LB (S11B Fig). Since the most prominent difference between the composition of TY and LB media is the content of NaCl (86 mM in LB, none in TY) and CaCl_2_ (2.7 mM in TY, none in LB), we analyzed the effects of these salts on the growth of RgsM-overproducing cells. Increasing the NaCl concentration to 300 mM in LB alleviated the *rgsM* overexpression-associated morphology and growth defects, and addition of CaCl_2_ to 2.7 mM resulted in wild type-like growth (S12 Fig). Replacement of Zn^2+^ with Ca^2+^ in RgsM may inactivate the protein and thereby mitigate the effect of *rgsM* overexpression. However, this explanation for the effect of CaCl_2_ is unlikely since the presence of Ca^2+^ in the growth medium did not negatively affect growth of *S*. *meliloti*, as RgsM depeletion did. Thus, the growth defect resulting from *rgsM* overexpression in LB may be a combined effect of an artificial increase in RgsM abundance and outer membrane destabilization in the absence of calcium [32]. This was probably counteracted by elevated medium osmolarity, which is supposed to reduce turgor.

To analyze the putative RgsM metallopeptidase active site H_510_xxxD, we constructed the RgsM_H510A_ variant. The H510A mutation mitigated the strong cell morphology defect caused by RgsM overproduction in LB. Notably, both in TY and LB, growth of the *rgsM*_H510A_ overexpressing cells was inhibited and morphology was altered similar to RgsM-depleted cells (S11 Fig), suggesting a possible dominant negative effect of RgsM_H510A_ overproduction on the native RgsM function. Accumulation of overproduced RgsM_wt_ and RgsM_H510A_ in Rm2011 was confirmed by detecting the corresponding 3×FLAG-tagged variants (S1E Fig).

Overexpression of *rgsM*, but not *rgsM*_H510A_, compromised the cell envelope of *E*. *coli* and resulted in cell lysis in LB lacking NaCl. This was detected using the β-galactosidase substrate chlorophenol red-β-D-galactopyranoside (CPRG) as an indicator of cell lysis and increased membrane permeability [33] and by microscopy (Fig 7E; S13A Fig). As in *S*. *meliloti*, overproduction of wild type RgsM and RgsM_H510A_ inhibited growth of *E*. *coli* in LB medium (S13B Fig). Both corresponding 3×FLAG-tagged RgsM variants accumulated in *E*. *coli* (S1E Fig).

### RgsP and RgsM affect PG composition

**Fig 8:**
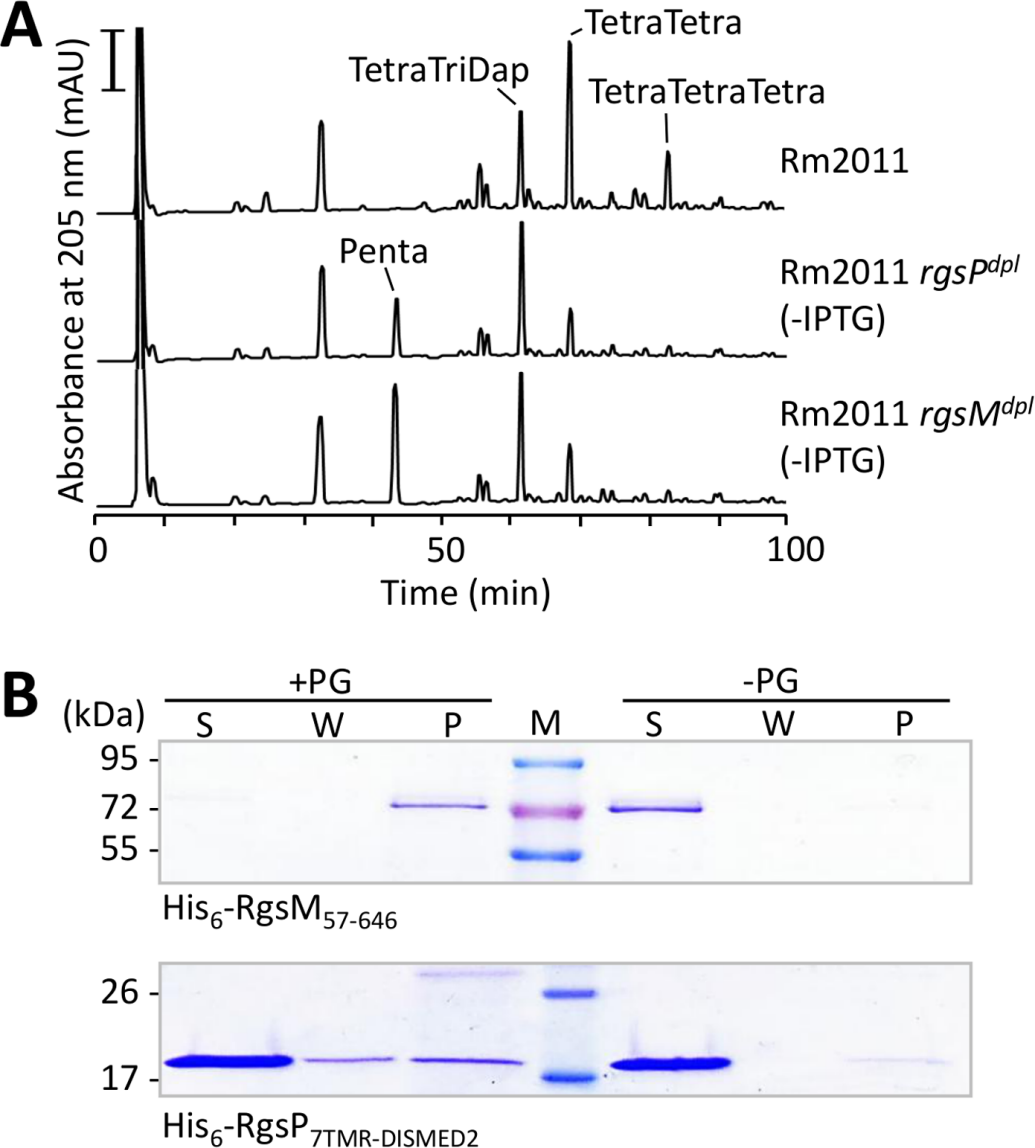
RgsP and RgsM depletion cause alterations in PG muropeptide composition and RgsM is able to bind PG *in vitro*. (A) HPLC profiles of muropeptides of RgsP and RgsM-sufficient cells (Rm2011) and corresponding depletion strains (Rm2011 *rgsP*^*dpl*^ and Rm2011 *rgsM*^*dpl*^) grown without IPTG for 24 h. The most prominent muropeptides that differ relative to wild type are indicated. Representative profiles of two independent biological replicates are shown. Bar, 100 mAU. (B) PG binding ability of His_6_-RgsM_57-646_ or His_6_-RgsP_7TMR-DISMED2_ was assessed in an *in vitro* binding assay with *S*. *meliloti* Rm2011 PG sacculi followed by SDS-PAGE and Coomassie blue staining. S, supernatant from the first centrifugation step. W, supernatant from the washing step. P, pellet. PG, peptidoglycan. M, molecular weight marker. Control reactions were performed in the absence of PG sacculi.

To investigate the role of RgsP and RgsM in cell wall biogenesis, we determined the muropeptide composition of PG isolated from *S*. *meliloti* Rm2011 depleted and non-depleted of RgsP or RgsM. Muropeptide profiles of the depletion strains grown in TY under non-depletion conditions were similar to those obtained for the Rm2011 wild type (S14A Fig). Depletion of RgsP or RgsM resulted in alterations in the relative abundance of specific muropeptides. In both strains, the uncross-linked pentapeptides and the 3-3-cross-linked TetraTri dimers accumulated, whereas the 4-3-cross-linked TetraTetra dimers and TetraTetraTetra trimers were less abundant than in non-depleted cells (Fig 8A; S14 and S15 Figs; S4 Table).

Rm2011 cells, overproducing RgsM in LB, accumulated more Penta and TetraPenta muropeptides compared to cells harboring the empty vector control, whereas overproduction of RgsM_H510A_ resulted in similar but less pronounced changes in the muropeptide profiles (S16A Fig). The accumulation of pentapeptides upon either depletion or overproduction of RgsP and RgsM points to altered incorporation and/or processing of new material into the growing PG sacculus, and the increase in 3–3 cross-links suggests increased LD-transpeptidase activity. Both might be indirect effects in cells with impaired PG growth, in response to stress. Muropeptides of *E*. *coli* S17-1 overproducing RgsM or RgsM_H510A_ and grown in LB remained unchanged suggesting that the phenotypic changes of *E*. *coli* cells were independent of a putative PG hydrolase activity of RgsM (S16B Fig).

To gain further mechanistic insights into RgsP and RgsM functions in *S*. *meliloti*, purified His_6_-tagged variants of both proteins were assayed for PG binding *in vitro*. Whereas His_6_-RgsM_57-646_ was recovered in the insoluble fraction after incubation with PG, His_6_-RgsP_7TMR-DISMED2_ mostly remained in the soluble fraction (Fig 8B). This suggested that RgsM bound tightly to PG and that this was not the case for the periplasmic domain of RgsP (RgsP_7TMR-DISMED2_).

PG hydrolase activity of His_6_-RgsM_57-646_ alone or in combination with His_6_-RgsP_7TMR-DISMED2_ was assayed with *S*. *meliloti* PG as substrate. No evident changes in the muropeptide profiles were observed, indicating that His_6_-RgsM_57-646_ did not hydrolyze PG *in vitro* (S17A Fig). Since we observed a prominent proteolysis product of RgsM in Western blot analysis (S1E Fig), we asked if this might represent the active form of the enzyme. Therefore, N-terminally truncated version of His_6_-RgsM (amino acids 260–646) was analyzed. Although this fragment retained the ability to bind PG, it did not show PG hydrolase activity *in vitro* (S17B and S17C Fig). It cannot be excluded that the assay conditions were not optimal or that an additional factor is required to activate RgsM. Overall, the effects of RgsM and RgsP depletion or overproduction on the muropeptide profiles and strong PG binding by RgsM further support involvement of both proteins in PG biogenesis.

### RgsP and RgsM functions are conserved in α-rhizobial species

Comparative protein sequence analysis using BLASTP revealed conservation of RgsP and RgsM in Rhizobiales, Rhodobacterales, and in a single γ-proteobacterial species, whereas no homologs were detected in the remaining eubacterial phyla (S3 Table). Thus, we asked whether the homologous proteins from other species were also functionally conserved. *Rhizobium etli* and *A*. *tumefaciens rgsP* homologs *RHE_CH00976* (*rgsP*_Re_) and *Atu0784* (*rgsP*_At_) were translationally fused to *egfp* at their native genomic locations. These tagged proteins displayed polar and septal localization corresponding to the HADA-staining zones in *R*. *etli* and *A*. *tumefaciens*, similar to the labeled RgsP variant in *S*. *meliloti* (S18A Fig; Fig 2). We also tested the ability of *A*. *tumefaciens* and *R*. *etli rgsP* and *rgsM* homologs, ectopically expressed from a taurine-inducible promoter, to complement *S*. *meliloti* RgsP and RgsM depletion strains. Without promoter induction ('leaky expression'), *rgsP*_Re_, *rgsP*_At_ and *rgsM*_Re_ fully complemented the growth and morphology defects of the respective *S*. *meliloti* depletion strains, similar to the *S*. *meliloti* homologs, whereas induction with taurine was required for complementation of RgsM depletion with *rgsM*_At_ (S18B and S18C Fig). Similar subcellular localization of RgsP homologs in *S*. *meliloti*, *R*. *etli* and *A*. *tumefaciens*, and cross-complementation of *rgsP* and *rgsM* between these species provide evidence for functional conservation of both proteins in the Rhizobiales.

## Discussion

Members of the Rhizobiales, including *S*. *meliloti*, show unipolar cell growth [3]. However, the molecular mechanisms governing polar growth of the PG sacculus are largely unknown in these bacteria. In this study, we provide evidence suggesting important roles for RgsP (a member of the seven-transmembrane receptor family 7TMR-DISM) and its interaction partner RgsM (a putative PG metallopeptidase) in polar cell wall growth.

Proteins associated with the polar PG growth zones in *A*. *tumefaciens* include the division scaffold proteins FtsZ and FtsA, PG synthase PBP1a and LD-transpeptidase Atu0845 [34,35], which are directly or indirectly involved in PG biosynthesis. RgsP does not show homology to known cell division or PG biosynthesis proteins; however, it localizes to sites of zonal cell wall synthesis in *S*. *meliloti*, *R*. *etli* and *A*. *tumefaciens*. This feature is likely to be conserved in α-rhizobia containing a RgsP homolog. The phenotypes caused by RgsP depletion in *S*. *meliloti* − i.e. growth inhibition, altered cell shape, reduced incorporation of HADA and accumulation of penta-muropeptides in the PG sacculus − imply a regulatory role of RgsP in PG biosynthesis. Although known protein components of the cell elongation and division machineries have not been detected in the RgsP and RgsM pull-down assays, indirect, transient or low affinity interactions of RgsP or RgsM with such proteins may have escaped this analysis.

In *E*. *coli*, pentapeptides are specific for newly synthesized PG and are quickly processed as PG matures [36] by trans-, endo- and carboxypeptidase reactions [7]. In agreement with a previous report [3], we did not detect pronounced pentapeptide peaks in the *S*. *meliloti* wild type muropeptide profile. Perhaps in the wild type these pentapeptides were quickly processed but accumulated in RgsP-depleted cells due to impaired PG maturation. In *E*. *coli*, lack of the PG carboxypeptidase PBP5 results in an increase in the PG pentapeptide content; an effect which is further augmented by eliminating endopeptidases PBP4 and PBP7 [37]. Along with accumulation of monomeric pentapeptides, tetrapeptide dimer and trimer levels decreased upon RgsP depletion, indicating impaired PG cross-linking or enhanced hydrolysis of the peptide bridges. The cell shape can be influenced by the degree of PG cross-linking [38–44]. Hence, the impaired growth and altered cell shape of RgsP-depleted cells might have resulted from dysregulation of PG remodeling.

Cell wall biogenesis by necessity involves incorporation of new PG material into the existing PG sacculus, and this requires local hydrolysis of peptide bridges by PG endopeptidases. These include proteins with a M23 metallopeptidase (or LytM) domain. In *E*. *coli* and *Vibrio cholerae*, single genetic knockdowns of LytM domain proteins were not lethal because of genetic redundancy [45,46]. By contrast, the LytM domain protein RgsM is essential in *S*. *meliloti*, which is in agreement with a previous report [24]. The *A*. *tumefaciens* RgsM and RgsP orthologues were also shown to be essential [47]. Although we were not able to detect RgsM PG hydrolase activity *in vitro*, several findings are in agreement with an enzymatic activity *in vivo*. The RgsM LytM domain contains conserved histidine residues, which have been found to be essential for PG hydrolase activity [28,30,48]. Moreover, *rgsM* overexpression in *S*. *meliloti* destabilized the cell envelope and caused perturbations in the muropeptide composition. However, expression of *rgsM* in *E*. *coli* resulted in cell lysis but did not cause changes in the muropeptide profile. Structural studies of *S*. *aureus* LytM and *Neisseria meningitidi*s LytM domain protein NMB0315 suggested the full-length proteins to adopt conformations interfering with enzymatic function, implying activation by proteolysis or protein-protein interactions *in vivo* [28,49]. Thus, we speculate that *in vivo*, RgsM may hydrolyze PG once activated by a yet-unknown factor. Alternatively, RgsM may have a regulatory role similar to *E*. *coli* and *C*. *crescentus* LytM domain proteins, which activate amidases to cleave septal PG [50–54]. Some LytM domain proteins also contain PG-binding domains [53,55]. PG binding by RgsM, demonstrated *in vitro*, is in agreement with an enzymatic or regulatory role of this protein.

Based on the mutual pull-down of RgsP and RgsM, an interaction in a bacterial two-hydrid assay and similar phenotypes of RgsM- and RgsP-depleted cells, we suggest that both proteins are involved in the same regulatory pathway. We narrowed down the essential part of RgsP to the N-terminal section including the 7TMR-DISM and PAS domains, which both may have a sensory function. In bacteria, 7TMR-DISMs are best understood in *P*. *aeruginosa*. In this bacterium, ligand binding to 7TMR-DISMED2 domains and their homodimerization were described as regulatory cues [21,22,56,57]. We speculate that RgsP either homodimerizes or, once triggered by an unknown cue, interacts with RgsM, to modulate RgsM dimerization and activity. This model is in agreement with the similar phenotypes of RgsP- and RgsM-depleted cells and the dominant effect of RgsM_H510A_ overproduction in *S*. *meliloti*. This enzymatically inactive protein variant may compete with native RgsM for the interaction sites on RgsP or other interacting proteins.

Although abundance of RgsP lacking the PAS domain was only moderately reduced, the localization and growth-promoting function of this protein was dramatically affected. A role of PAS domains or PAS-like motifs in polar protein localization was previously reported for example in *C*. *crescentus*. These include the *C*. *crescentus* single PAS domain protein MopJ, which directly interacts with the polarly localized histidine protein kinases DivJ and CckA, which are both involved in cell cycle regulation [58,59]. We speculate that the PAS domain may be important for recruitment of RgsP to PG biosynthesis sites. The nature of the signal, perceived by the RgsP PAS domain, and its regulatory output remain to be investigated.

RgsP PDE activity substantially contributes to c-di-GMP turnover in *S*. *meliloti*. To our knowledge, RgsP is the only c-di-GMP PDE which is polarly localized in *S*. *meliloti*. Polarly localized c-di-GMP PDEs in *C*. *crescentus* and *P*. *aeruginosa* contribute to heterogeneity in the cellular c-di-GMP content [60–62]. Lack of the RgsP PDE activity did not cause apparent phenotypic defects. Yet, we cannot exclude that RgsP PDE activity has a regulatory function in *S*. *meliloti*, which may be linked to its localization. A regulatory function may have escaped our phenotypic analyses or may not have been detected because of compensation by any of the twelve additional c-di-GMP PDEs encoded by the *S*. *meliloti* genome [17].

Whereas the RgsP_Sm_ GGDEF domain seems to be inactive because of a degenerate active site [17], RgsP_At_ contains an intact GGDEF motif, suggesting enzymatic activity of this protein. Since RgsP_At_ complemented depletion of RgsP_Sm_ in *S*. *meliloti*, the DGC activity of RgsP is unlikely to be relevant for its growth-promoting function. However, the GGDEF domain may modulate PDE activity of the EAL domain, as for example in case of *C*. *crescentus* PdeA or *P*. *aeruginosa* BifA and RmcA [61,63,64].

Overall, our study suggests that compared to well-studied γ-proteobacterial models, α-rhizobia utilize different sets of proteins for PG metabolism during cell elongation and division, and for relocating the PG growth machinery from a pole to the cell division site. Identification of further enzymes involved in PG synthesis and remodeling, and understanding the regulatory roles of RgsP and RgsM will lead to the clarification of specific mechanisms for polar PG biogenesis in α-rhizobia.

## Methods

### Bacterial strains and growth conditions

Bacterial strains and plasmids used in this study are shown in S5 Table. *S*. *meliloti* was grown at 30°C in tryptone-yeast extract (TY) medium [65], LB medium [66], modified MOPS-buffered minimal medium [67], and nutrient-depleted 30% minimal medium (nitrogen, carbon, and phosphate sources reduced to 30%). *R*. *etli* was grown in TY medium and *A*. *tumefaciens* was grown in LB medium at 30°C. *E*. *coli* was grown in LB medium at 37°C. For *S*. *meliloti*, antibiotics were used at the following concentrations (mg/l; liquid/solid medium): kanamycin, 100/200, gentamicin, 15/40, tetracycline, 5/10, spectinomycin, 200/200 and streptomycin, 600/600. For *E*. *coli* the following concentrations were used: kanamycin, 50/50, gentamicin, 5/8, tetracyclin, 5/10, spectinomycin 100/100, and ampicillin 100/100. For *A*. *tumefaciens* kanamycin was used at 100/200 and for *R*. *etli* at 30/30. Unless otherwise specified, the inducers isopropyl β-D-1-thiogalactopyranoside (IPTG) and taurine were added at 0.5 and 20 mM, respectively. Chlorophenol red-β-D-galactopyranoside (CPRG), an almost membrane-impermeable β-galactosidase substrate previously used to detect compromised *E*. *coli* cell envelope and lysis by purple staining of agar cultures [33], was added at 20 μg/ml. Alkaline phosphatase substrate 5-bromo-4-chloro-3-indolylphosphate (BCIP) was used at 50 μg/ml and β-galactosidase substrate 5-bromo-4-chloro-3-indolyl-β-D-galactopyranoside (X-Gal) was used at 40 μg/ml.

Growth assays were performed using 100 μl cultures in flat-bottom 96-well plates (Greiner), grown at 30 ^o^C with shaking at 1,200 rpm. Three to six culture replicates were analyzed per strain. Optical density (OD_600_) was recorded using Infinite M200 PRO fluorescence reader (Tecan). For growth assays involving protein depletion, cultures with or without IPTG were inoculated with 0.15 μl of stationary TY preculture with IPTG and grown for 24 h. Relative growth was calculated as a ratio of OD_600_ of cells grown without IPTG and OD_600_ of cultures supplemented with 0.5 mM IPTG. For growth assays, involving taurine- or IPTG-induced gene overexpression, the cultures containing or not containing taurine or IPTG were inoculated with 0.15 μl of stationary TY preculture and the growth was recorded at the indicated time points.

### Media

TY medium (5 g/l tryptone, 3 g/l yeast extract, 0.4 g CaCl_2_×2H_2_O). LB medium (10 g/l tryptone, 5 g/l yeast extract, 5 g/l NaCl). MOPS-buffered minimal medium (MM) (10 g/l MOPS, 10 g/l mannitol, 3.55 g/l sodium glutamate, 0.246 g/l MgSO_4_×7H_2_O, 0.25 mM CaCl_2_, 2 mM K_2_HPO_4_, 10 mg/l FeCl_3_×6H_2_O, 1 mg/l biotin, 3 mg/l H_3_BO_3_, 2.23 mg/l MnSO_4_×H_2_O, 0.287 mg/l ZnSO_4_×7H_2_O, 0.125 mg/l CuSO_4_×5H_2_O, 0.065 mg/l CoCl_2_×6H_2_O, 0.12 mg/l NaMoO_4_×2H_2_O, pH 7.2).

### Construction of strains and plasmids

Constructs used in this work were generated using standard cloning techniques and are listed in S5 Table. The primers used are listed in S6 Table. All constructs were verified by sequencing. Plasmids were transferred to *S*. *meliloti* by *E*. *coli* S17-1-mediated conjugation as previously described [68]. Electroporation was used to introduce plasmids to *R*. *etli* and *A*. *tumefaciens* following the protocol previously described [69].

To generate chromosomally integrated constructs encoding RgsP or RgsM with C-terminally fused enhanced green fluorescent protein (EGFP) or triple FLAG-tag (3×FLAG), the 700 to 800 bp 3' portion of the gene excluding the stop codon was cloned into suicide plasmids pK18mob2-*egfp* or pG18mob-*3×flag* yielding translational fusions of the C-terminal portion of the protein coding sequence to the corresponding tag. Integration of these gene fusion constructs into the *S*. *meliloti* genome by homologous recombination resulted in a replacement of the native gene copy with *egfp*- or *3xflag*-tagged gene copy at the corresponding native chromosomal location.

To construct markerless translational fusions of *rgsP* and *rgsM* to *egfp* or *mCherry* at the native chromosomal location, the 700–800 bp 3' portion of the gene fused to *egfp* or *mCherry* was cloned into suicide plasmid pK18mobsacB [70] together with 700–800 bp of adjacent downstream genomic region. The resulting constructs were introduced into *S*. *meliloti* and transconjugants were subjected to sucrose selection to obtain double recombinants that have lost the integrated vector [70]. Correct positions of chromosomally encoded gene fusions were verified by PCR.

To obtain RgsP and RgsM depletion strains, plasmids designed to uncouple the native promoter from the coding sequence and to place the coding sequence under the control of IPTG-inducible promoters were constructed. The *rgsP* gene is most likely co-transcribed with the preceding *rimJ* gene, encoding a probable ribosomal-protein-alanine acetyltransferase (S7A Fig). To uncouple transcription of *rimJ* and *rgsP*, and to place *rgsP* under the control of an IPTG-inducible promoter, the *lac-*T5 tandem promoter sequence was inserted between *rgsP* and *rimJ* without altering the *rimJ* open reading frame. To this end, a DNA fragment containing the IPTG-inducible T5 promoter and a Shine-Dalgarno sequence followed by the partial 5' *rgsP* coding sequence starting from the start codon (586 bp) was PCR-amplified from *rgsP* expression plasmid pWBT-*SMc00074* [17]. This fragment was inserted into pK18mob2 [70] downstream of the IPTG-inducible *lac* promoter, thus generating a *lac*-T5 tandem promoter. In the case of RgsM depletion, the partial 5' *rgsM* coding sequence starting from the start codon (406 bp) was PCR-amplified from *S*. *meliloti* genomic DNA and inserted into pK18mob2 downstream of the IPTG-inducible *lac* promoter. Integration of these constructs into the genome placed the full-length protein coding sequence under the control of the corresponding IPTG-inducible promoter. Adjacent open reading frames were not affected by integration of these constructs. Conditional RgsP and RgsM depletion was then achieved by constitutively expressing the *lacI* repressor gene from vector pSRKGm in the strains Rm2011 *rgsP*^*dpl*^ and Rm2011 *rgsM*^*dpl*^, respectively.

Gene overexpression constructs were generated by insertion of the corresponding coding sequences downstream of either the IPTG-inducible *lac* promoter in medium-copy vectors pSRKKm and pSRKGm [71], the IPTG-inducible T5 promoter in medium-copy vector pWBT, the taurine-inducible *tauA* promoter in low-copy vector pR-P_*tau*_, or a constitutive synthetic promoter (*P*_*syn*_) in low-copy vector pR_*egfp* [72]. In the case of pR_*egfp*, a 114 bp region upstream of the *rgsP* coding region was included.

To generate single-copy ectopic *rgsP* or *rgsP-egfp* expression constructs, we first determined the native *rgsP* promoter and its activity levels. At its native chromosomal locus, *rgsP* is most likely in an operon with *rimJ*. Therefore, a 300 bp genomic region upstream of *rgsP* as well as a 944 bp region including the putative *rimJ* promoter and the whole *rimJ* coding sequence (S7A Fig) were tested for promoter activity using fusions to *egfp* (S7B Fig). Since higher levels of *egfp* expression were observed in the case of the 944 bp fragment, we used this DNA region as native *rgsP* promoter. To exclude possible non-desirable effects of an additional *rimJ* copy, a nonsense mutation was introduced 64 nucleotides downstream of the *rimJ* start codon yielding promoter construct P*_rgsP_ (S7A Fig). The *rgsP*, *rgsP-egfp* or *rgsP-3*×*flag* coding sequences were inserted downstream of the P*_rgsP_ sequence in single-copy plasmid pABC2S-mob [73]. For generation of amino acid substitutions or protein variants lacking specific domains splicing by overlap extension PCR was applied.

The *rgsP* promoter-*egfp* fusions were generated by insertion of the upstream non-coding region (either long (944 bp) or short (300 bp)) and the three first codons of *rgsP* into replicative medium-copy number plasmid pSRKKm-*egfp* [17]. Constructs for purification of His_6_-tagged proteins were generated by insertion of the coding sequence excluding the start codon into expression vector pWH844 [74]. Fusions of *rgsM* to *phoA* were assembled from the full-length or partial *rgsM* coding sequence and the *E*. *coli phoA* coding sequence missing the first 26 codons.

### Fluorescence measurements

For promoter-*egfp* activity assays, TY overnight cultures were diluted 1:500 in 100 μl of TY medium or 30% MM and grown in 96-well plates at 30°C with shaking at 1,200 rpm. EGFP fluorescence (excitation 488 ± 9 nm; emission 522 ± 20 nm, gain 82) and OD_600_ were determined using the Infinite 200 Pro multimode reader (Tecan) and calculated as relative fluorescence units (RFU), which represent fluorescence values divided by OD_600_. Background EGFP fluorescence was determined using a control strain harboring pSRKKm-*egfp*. Fluorescence of three independent transconjugants was measured as biological replicates.

### Fluorescence microscopy

Microscopy of bacteria on 1% agarose pads was performed using the Nikon microscope Eclipse Ti-E equipped with a differential interference contrast (DIC) CFI Apochromat TIRF oil objective (100x; numerical aperture of 1.49) and a phase-contrast Plan Apo l oil objective (100x; numerical aperture, 1.45) with the AHF HC filter sets F36-513 DAPI (excitation band pass [ex bp] 387/11 nm, beam splitter [bs] 409 nm, and emission [em] bp 447/60 nm), F36-504 mCherry (ex bp 562/40 nm, bs 593 nm, and em bp 624/40 nm), F36-525 EGFP (ex bp 472/30 nm, bs 495 nm, and em bp 520/35 nm) and F36-528 YFP (ex bp 500/24 nm, bs 520 nm, and em bp 542/27 nm). Images were acquired with an Andor iXon3 885 electron-multiplying charge-coupled device (EMCCD) camera. For time-lapse analysis, MM 1% agarose pads were used, and images were acquired every 2, 4 or 15 min at 30°C. IPTG was added to the medium at 0.2 or 0.5 mM for microscopy of cells harboring pSRKGm-*parB-mCherry* or pSRKGm-*mCherry-ftsZ*, respectively.

Treatment of *S*. *meliloti*, *R*. *etli* and *A*. *tumefaciens* cells with fluorescently-labeled D-amino acid HADA was performed as previously described [75]. Briefly, cells were grown for 24 h in liquid medium in glass tubes to an OD_600_ of 0.4–0.6. 80 μl of the cultures were then mixed with 0.25 μl 100 mM HADA solution and incubated for 2.5 min (*A*. *tumefaciens*), 3 min (*S*. *meliloti*) or 3.5 min (*R*. *etli*) at 30 ^o^C with shaking at 800 rpm. After addition of 186 μl 100% ethanol and 5–20 min incubation at room temperature (RT), cells were washed three times with 0.9% NaCl and subsequently placed onto 1% agarose pads.

Cell viability was assessed by DNA staining with the fluorescent intercalating agent propidium iodide. 100 μl of *S*. *meliloti* liquid cultures (OD_600_ 0.4–0.8) were mixed with 1 μl of 2 mg/ml propidium iodide stock solution and incubated for 5 min at room temperature. Cells were washed three times with 0.9% NaCl and subsequently placed onto 1% agarose pads.

### Transmission electron microscopy

Concentrated *S*. *meliloti* cell suspensions were high pressure frozen (HPF Compact 02, Wohlwend, CH) and freeze-substituted (AFS2, Leica, Wetzlar, Germany) in a medium based on acetone, containing 0.25% osmium tetroxide, 0.2% uranyl acetate and 5% ddH_2_O according to the following protocol: -90°C for 20 h, from -90°C to -60°C in 1 h, -60°C for 8 h, -60°C to -30°C in 1 h, -30°C for 8 h, -30°C to 0°C in 1 h, 0°C for 3 h. Still at 0°C, samples were washed three times with acetone before a 1:1 mixture of Epon 812 substitute resin (Fluka, Buchs, CH) and acetone was applied at room temperature for 2 h. The 1:1 mixture was substituted with pure resin to impregnate the samples overnight. After another substitution with fresh Epon, samples were polymerized at 60°C for 2 days. The sample containing polymerized Epon blocks were then trimmed with razor blades and cut to 50 nm ultrathin sections using an ultramicrotome (UC7, Leica, Wetzlar, Germany) and a diamond knife (Diatome, Biel, Switzerland). Sections were applied onto 100 mesh copper grids coated with pioloform. For additional contrast, mounted sections were post-stained with 2% uranyl acetate for 20–30 min and subsequently with lead citrate for another 1–2 min. The sections were finally analyzed and imaged using a JEM-2100 transmission electron microscope (JEOL, Tokyo, Japan) equipped with a 2k x 2k F214 fast-scan CCD camera (TVIPS, Gauting, Germany).

### Quantification of intracellular c-di-GMP

Strain Rm2011 *rgsP*^*dpl*^ ectopically expressing *rgsP* variants from P*_rgsP_ was grown in triplicates in liquid TY medium without IPTG and harvested in the exponential growth phase 24 h after inoculation. Quantification of intracellular c-di-GMP was performed as previously described [76]. Briefly, cells were collected by centrifugation and nucleotides were extracted three times with acetonitrile/methanol/water (2:2:1), dried and subjected to liquid chromatography-tandem mass spectrometry. c-di-GMP was normalized to total protein, determined using Bradford reagent (Bio-Rad).

### Protein purification

Heterologous protein expression and purification was performed as previously described [17]. *E*. *coli* BL21(DE3) harboring expression plasmids were grown in LB medium in flasks to OD_600_ of 0.5–0.6 and protein expression was induced with 0.4 mM IPTG overnight at RT. Cells were lysed using French press (pressure 1,000 lb/in^2^) and the lysates were centrifuged for 60 min at 24,000 ×*g* and 4°C. Cleared lysates were applied to His SpinTrap columns (GE Healthcare) following the manufacturer’s instructions and eluted with 0.5 M imidazole. Purity of isolated proteins was assessed by SDS-PAGE and Coomassie brilliant blue staining. Protein concentration was determined using Bradford reagent (Bio-Rad).

### Preparation of [α-^32^P]-labeled c-di-GMP

[α-^32^P]-labeled c-di-GMP was synthesized using purified *Caulobacter crescentus* His_6_-DgcA at 10 μM from GTP and [α-^32^P]-GTP (0.1 μCi/μl) at 1 mM in the reaction buffer (50 mM Tris-HCl, 300 mM NaCl, 10 mM MgCl_2,_ pH 8.0), overnight at 30°C. The reaction was then treated with 5 units of calf intestine alkaline phosphatase (Fermentas) for 1 h at 22°C to hydrolyze unreacted GTP and stopped by incubation for 10 min at 95°C. The precipitated proteins were removed by centrifugation (10 min, 20,000 ×*g*, 22°C) and the supernatant was used for the PDE activity and the c-di-GMP binding assays.

### *In vitro* c-di-GMP binding assay

c-di-GMP binding was determined using a differential radial capillary action of ligand assay (DRaCALA) with [α-^32^P]-labeled c-di-GMP, as previously described [77]. This assay is based on the ability of dry nitrocellulose to prevent diffusion of bound protein-ligand complexes and thereby separate them from free ligand. Reaction mixtures (50 μl) containing [α-^32^P]-labeled c-di-GMP and 20 μM of indicated protein in the binding buffer (10 mM Tris, 100 mM NaCl, 5 mM MgCl_2,_ pH 8.0) were incubated for 10 min at RT. 10 μl of this reaction mixture was spotted onto nitrocellulose membrane and allowed to dry prior to exposing a phosphor-imaging screen (Molecular Dynamics). Data were collected using a STORM 840 scanner.

### *In vitro* DGC and PDE activity assay

DGC and PDE activities were determined as previously described [78,79]. Reaction mixtures (40 μl) containing purified proteins at 10 μM, in reaction buffer (50 mM Tris-HCl, 300 mM NaCl, 10 mM MgCl_2_, pH 8.0) were first pre-incubated for 5 min at 30°C. DGC reactions were initiated by adding GTP/[α-^32^P]-GTP (0.1 μCi/μl) to 1 mM, incubated at 30°C for the indicated periods of time and stopped by adding an equal volume of 0.5 M EDTA. PDE reactions were initiated by adding [α-^32^P]-labeled c-di-GMP and stopped by adding an equal volume of 0.5 M EDTA after indicated time periods. 2 μl of the PDE or DGC reaction mixtures were spotted on polyethyleneimine-cellulose TLC chromatography plates, developed in 2:3 (v/v) 4 M (NH_4_)_2_SO_4_/1.5 M KH_2_PO_4_ (pH 3.65). Plates were dried prior to exposing a phosphor-imaging screen (Molecular Dynamics). Data were collected and analyzed using a STORM 840 scanner (Amersham Biosciences).

### Co-immunoprecipitation (Co-IP) and protein identification by mass spectrometry

Co-IP and protein identification by mass spectrometry was performed as previously described including small modifications [80]. Cultures of Rm2011 *rgsP-3*×*flag*, Rm2011 *rgsM-3*×*flag* and control strain Rm2011 harboring the empty vector pWBT were grown in TY medium in flasks to an OD_600_ of 0.6 and cross-linked with 0.1% formaldehyde for 15 min at RT. Reaction was quenched by adding glycine at a final concentration of 0.35 M. Cells were washed, resuspended in lysis buffer (50 mM Tris-HCl, 150 mM NaCl, 1 mM EDTA, 1% Triton X-100, 2 mM phenylmethylsulfonyl-fluorid [PMSF], pH 7.4) and lysed using a French press (pressure 1,000 lb/in^2^). Cleared lysates obtained after ultracentrifugation (100,000 ×*g*, 1 h, 4°C) were incubated with anti-FLAG M2 affinity gel (FLAG Immunoprecipitation Kit, Sigma) overnight at 4°C on a rolling shaker. Bound proteins were eluted with 3×FLAG peptide solution.

For mass-spectrometry analysis, proteins were digested by Sequencing Grade Modified Trypsin (Promega) at 37°C overnight. The mass spectrometric analysis was performed using an Orbitrap Velos Pro mass spectrometer (Thermo Fisher Scientific). An Ultimate nanoRSLC-HPLC system (Dionex), equipped with a custom 20 cm x 75 μm C18 RP column filled with 1.7 μm beads was connected online to the mass spectrometer through a Proxeon nanospray source. 1-15 μl of the tryptic digest were injected onto a C18 pre-concentration column. Automated trapping and desalting of the sample was performed at a flow rate of 6 μl/min using water/0.05% formic acid as solvent. Separation of the tryptic peptides was achieved with the following gradient of water/0.05% formic acid (solvent A) and 80% acetonitrile/0.045% formic acid (solvent B) at a flow rate of 300 nl/min: holding 4% B for 5 min, followed by a linear gradient to 45% B within 30 min and linear increase to 95% solvent B in additional 5 min. The column was connected to a stainless steel nanoemitter (Proxeon, Denmark) and the eluent was sprayed directly towards the heated capillary of the mass spectrometer using a potential of 2,300 V. A survey scan with a resolution of 60,000 within the Orbitrap mass analyzer was combined with at least three data-dependent MS/MS scans with dynamic exclusion for 30 s either using CID with the linear ion-trap or using HCD combined with orbitrap detection at a resolution of 7,500. Data analysis was performed using Proteome Discoverer (Thermo Fisher Scientific) with SEQUEST and MASCOT (version 2.2; Matrix science) search engines using either SwissProt or NCBI databases. The combined transmembrane topology and signal peptide prediction online tool PHOBIUS [27] was used to predict regions with high hydrophobicity within candidate protein interactions partners of RgsP and RgsM.

### Bacterial two-hybrid analysis and β-galactosidase activity assay

Bacterial two-hybrid analysis was performed as previously described [31]. The adenylate cyclase-deficient strain *E*. *coli* BTH101 was co-transformed with plasmids carrying *rgsP*_∆GGDEF∆EAL_ or *rgsM* translationally fused to T25 and T18 fragments of *Bordetella pertussis* adenylate cyclase. Transformant colonies were grown in 100 μl LB supplemented with antibiotics at 30°C for 6 hours with shaking 1,200 rpm. 10 μl of each culture was spotted onto LB agar plates containing kanamycin, ampicillin, X-Gal and IPTG. Plates were imaged after 30 h of incubation at 30°C.

β-galactosidase activity was determined as previously described with small modifications [52]. Cells grown on LB agar containing IPTG were resuspended in 1 ml of Z-buffer (60 mM Na_2_HPO_4_, 40 mM NaH_2_PO_4_, 10 mM KCl, 1 mM MgSO_4_, pH 7.0), in triplicates and the OD_600_ was recorded. Cell permeabilization was facilitated by addition of 50 μl of chlorophorm and 50 μl of 0.05% SDS. The aqueous phase was mixed with an equal volume of Z-buffer containing 50 mM β-mercaptoethanol, and *ortho*-nitrophenyl-β-D-galactopyranoside (ONPG) was added to the final concentration of 0.5 mg/ml. A_420_ was recorded using the Infinite M200 PRO fluorescence reader (Tecan). Miller Units (MU) were calculated as follows: MU = 1000*A_420_/(t*V*OD_600_). t represents the time in min and V the volume in ml.

### Western blot

Western blot analysis was performed as previously described [68]. Briefly, *S*. *meliloti* and *E*. *coli* strains expressing *3×flag*-tagged *rgsP* or *rgsM* variants were grown in glass tubes supplemented with corresponding antibiotics. Unless otherwise specified, cells were grown in TY medium without IPTG and collected at an OD_600_ of 0.4–0.8 24 h after inoculation. Cells were adjusted to an OD_600_ of 1, 10 μl of lysed cells were loaded to SDS-PAGE gel and separated proteins were transferred to PVDF membrane (Thermo Fisher Scientific). RgsP protein variants were detected using anti-FLAG M2-Peroxidase (HRP) antibody (Sigma-Aldrich). Membranes were incubated with Pierce ECL Western Blotting Substrate (Thermo Fisher Scientific) and imaged using the luminescence image analyzer LAS-4000 (Fujifilm).

### Peptidoglycan purification and muropeptide analysis

Isolation of PG sacculi was achieved following a published protocol [81]. Briefly, *S*. *meliloti* strains were grown in 400 ml TY and LB media in flasks for 24 h at 30°C until OD_600_ reached 0.4–0.8. *E*. *coli* strains were grown in 400 ml LB for 4 h at 37°C until OD_600_ reached 0.5–0.9. Cells were harvested by centrifugation, resuspended in PBS buffer, added dropwise to boiling 8% SDS solution and stirred for 30 min. PG sacculi were pelleted by ultracentrifugation in a Beckman Coulter Optima at 440,000 ×*g*, at ambient temperature for 1 h and resuspended in water. Centrifugation and resuspension steps were repeated until the sacculi were free of SDS as verified by a published assay [82]. The sacculi were then incubated with 100 μg/ml of α-amylase in reaction buffer (10 mM Tris-HCl, 10 mM NaCl, pH 7.0) for 2 h at 37°C, followed by incubation with 200 μg/ml pronase E for 1 h at 60°C, to remove high molecular weight glycogen and proteins, respectively. The enzymes were removed by incubation in 4% SDS solution for 15 min at 80°C. Sacculi were washed free of SDS as described above and resuspended in 0.02% sodium azide.

Purified sacculi were digested with the muramidase cellosyl (Hoechst, Frankfurt, Germany) at 37°C overnight. The reaction was terminated by boiling the sample at 100°C for 10 min. The samples were centrifuged (15,000 ×*g* for 10 min) and the soluble muropeptides were recovered from the supernatant, reduced with NaBH_4_ and separated by HPLC as described for *E*. *coli* [81]. Muropeptides were then separated on a C18 reversed-phase column (ProntoSIL) using an Agilent 1220 infinity HPLC system. Separation was carried out over a 180 min linear gradient from buffer A (50 mM sodium phosphate, pH 4.31) to buffer B (75 mM sodium phosphate, pH 4.95, 15% methanol). Muropetides were detected by their absorbance at 205 nm. Muropeptides of interest corresponding to major peaks were manually collected and analyzed by tandem mass spectrometry (MS/MS) as previously described [83].

### Peptidoglycan binding assay

Purified PG (~100 μg) from *S*. *meliloti* strain Rm2011 was centrifuged at 15,000 ×*g*, 4°C for 14 min and resuspended in binding buffer (10 mM Tris-maleate, 10 mM MgCl_2_, 50 mM NaCl, pH 6.8). 10 μg of protein of interest was incubated with or without PG in a final volume of 100 μl, and incubated at 4°C for 30 min. Samples were centrifuged as described above and the supernatant was collected (supernatant fraction) whilst the pellet was resuspended in 200 μl of binding buffer. Another centrifugation step as described above was carried out (wash fraction). Bound proteins were released from PG by incubation with 100 μl of 2% SDS, at 4°C for 1 h before being collected by a final centrifugation step as done at earlier steps. The proteins present in the different fractions were analyzed by SDS-PAGE.

### Peptidoglycan hydrolase activity assay

Proteins (2 μM final concentration) were incubated with 10 μg of sacculi from *S*. *meliloti*, overnight at 37°C, in a volume of 100 μl (containing 10 mM HEPES-NaOH, 1 mM ZnCl_2,_ 150 mM NaCl, 0.05% Triton X-100, pH 7.5). Proteins were inactivated by boiling for 10 min. Cellosyl was added (1 μM final concentration) and the samples were incubated overnight again at 37°C. Samples were boiled for 10 min and the soluble muropeptides were collected by centrifugation at 15,000 ×*g* for 10 min, and taking the supernatant fraction. Muropeptides were reduced with NaBH_4_ and analyzed by HPLC as described above.

## Supporting information

S1 Fig RgsP-3×FLAG and RgsM-3×FLAG protein abundance determined by immunoblotting with α-FLAG antibody. 3×FLAG-MucR produced from the native genomic location was used as a loading control. (A) Growth stage-dependent abundance of RgsP and RgsM. Cells producing RgsP-3×FLAG or RgsM-3×FLAG from the native genomic location were harvested at indicated culture OD_600_. (B) Conditional depletion of RgsP and RgsM. Analyzed cells produced RgsP-3×FLAG or RgsM-3×FLAG from the native genomic location. In depletion strains (‘dpl’), the corresponding gene fusions were placed under control of IPTG-inducible promoters. These strains were grown in presence and absence of added IPTG. (C,D) Accumulation of indicated RgsP variants, produced from P*_rgsP_ on a single-copy plasmid (C) or from *P*_*syn*_ on a low-copy plasmid (D). Mean gray values of bands corresponding to 3×FLAG-tagged RgsP variants are shown relative to RgsP_wt_-3×FLAG produced from P*_rgsP_ (C) or relative to RgsP_wt_-3×FLAG produced from the native genomic location (D). ‘x’ denotes the respective RgsP variant. (E) Accumulation of RgsM variants in *S*. *meliloti* and *E*. *coli*. Indicated strains were grown in presence of IPTG either in LB for 24 h (*S*. *meliloti*) or in LB lacking NaCl for 3 h (*E*. *coli*).
(TIF)

S2 Fig Time course of morphological changes upon RgsP and RgsM depletion in *S*. *meliloti*. (A) DIC microscopy images of RgsP and RgsM depletion strains grown in liquid TY medium with and without added IPTG at indicated time points. Time is given in hours. (B) Time-lapse DIC microscopy of RgsP and RgsM depletion strains, previously grown in TY medium without IPTG for 6 h and 4 h, respectively, and placed on TY agarose pads with and without added IPTG. Arrow heads indicate cells showing no substantial cell elongation prior to visible septum constriction. Time is given in hours. Bars, 5 μm.
(TIF)

S3 Fig Transmission electron microscopy of *S*. *meliloti* RgsP and RgsM depletion and overexpression strains revealed altered cell morphologies. (A,B) Depletion strains were grown in TY with and without added IPTG. (C) Rm2011, harboring either empty vector pWBT or *rgsM* overexpression plasmid pWBT-*rgsM* (*rgsM*^++^), grown in LB with IPTG. Bars, 1 μm.
(TIF)

S4 Fig RgsP and RgsM affect viability of *S*. *meliloti* cells. (A) RgsP and RgsM depletion strains were grown in TY medium with and without added IPTG for 24 h. Cells were stained with propidium iodide and analyzed by fluorescence microscopy. The proportion of red fluorescent cells is indicated. *n*, total number of cells considered for statistical analysis. Bars, 5 μm. (B) After cultivation of cells under depletion (TY medium without IPTG) or non-depletion conditions (TY medium with IPTG) for 0, 12 and 24 h, the number of colony forming units (CFU) was determined by plating cells on TY medium with IPTG, followed by 72 h incubation. Error bars represent the standard deviation of three biological replicates. (C) Time-lapse DIC microscopy of RgsP- and RgsM-depleted cells, previously grown in TY medium without IPTG for 24 h, followed by incubation on TY agarose pads with or without IPTG. Time is given in hours. The proportion of dividing cells is indicated. *n*, total number of cells considered for statistical analysis. Bars, 5 μm.
(TIF)

S5 Fig Quantitative analysis of HADA staining and septum constriction of *S*. *meliloti* RgsP and RgsM depletion and overexpression strains. Depletion strains were grown in TY medium with and without added IPTG and Rm2011, harboring either empty vector pWBT or *rgsM* overexpression plasmid pWBT-*rgsM* (*rgsM*^++^), was grown in LB medium with IPTG for 24 h. (A) Relative proportion of cells analyzed by fluorescence microscopy and displaying HADA signal at one cell pole (pole), at mid-cell (mid-cell), or no HADA focus (no focus). (B) Bacteria described in panel A were classified into cells with and without visible septum constriction. *n*, number of cells considered for statistical analysis.
(TIF)

S6 Fig Spatiotemporal co-localization of RgsP-mCherry and PleD-EGFP. (A) Time-lapse microscopy of Rm2011 *rgsP-mCherry pleD-egfp* strain, carrying the gene fusions at native genomic locations. Time is shown in minutes. Bar, 1 μm. (B) Accumulation of PleD-EGFP at the new pole relative to relocation of RgsP-mCherry signal from pole to the mid-cell was monitored by quantification of fluorescence signals in the respective cell areas. Error bars represent the standard deviation of three biological replicates which included the time-lapse microscopy images shown in panel A and two additional biological replicates.
(TIF)

S7 Fig Analysis of *rgsP* promoter region and design of P*_rgsP_ used for ectopic *rgsP* expression from pABC2S-mob. P*_rgsP_ consists of the *rimJ* promoter region followed by *rimJ* coding sequence with TGA stop codon introduced at nucleotide position 64. (A) *rgsP* upstream regions including the *rimJ* promoter and the whole *rimJ* coding sequence (upstream region 1), or only partial *rimJ* coding sequence (upstream region 2), followed by the first three codons of *rgsP*, translationally fused to *egfp* to estimate the promoter activity of these regions. (B) Normalized EGFP fluorescence of Rm2011, harboring medium-copy plasmids carrying the translational fusions depicted in panel A, grown in TY and minimal media. Error bars represent the standard deviation of three biological replicates.
(TIF)

S8 Fig Complementation of RgsP-depleted *S*. *meliloti* by ectopic expression of *rgsP* variants. (A) Recorded growth of Rm2011 *rgsP*^*dpl*^ and c-di-GMP^0^ strain Rm2011 ΔXVI *rgsP*^*dpl*^, ectopically expressing non-tagged and *egfp*-tagged *rgsP* variants from either P*_rgsP_ (vector pABC2S-mob, transcription strength similar to the native level) or *P*_*syn*_ (vector pR_*egfp*, elevated transcription strength compared to the native levels), grown in absence or presence of IPTG. OD_600_ values were recorded after 24 h of growth. Error bars represent the standard deviation of three biological replicates. (B) Subcellular localization of RgsP-EGFP in Rm2011 and Rm2011 ΔXVI. Exponentially growing cells were analyzed by fluorescence microscopy. Bars, 5 μm.
(TIF)

S9 Fig The EAL domain of RgsP shows PDE activity, whereas the GGDEF domain of RgsP neither shows DGC activity nor binds c-di-GMP *in vitro*. (A,B) Thin-layer chromatograms of PDE reactions with purified His_6_-RgsP_PAS-GGDEF-EAL_ or His_6_-RgsP_PAS-GGDEF-EAL_-AAL and [α-^32^P]-c-di-GMP (A) and DGC reactions with purified His_6_-RgsP_PAS-GGDEF-EAL_-AAL or positive control His_6_-DgcA and [α-^32^P]-GTP (B). Incubation time is given in minutes. Arrows indicate the direction of mobile phase migration. P_i_, inorganic phosphate; pGpG, linear di-GMP. (C) DRaCALA with purified His_6_-RgsP_PAS-GGDEF_ and His_6_-DmxB as positive control. (D) c-di-GMP content of Rm2011 *rgsP*^*dpl*^, expressing the indicated *rgsP* variants from P*_rgsP_, grown in TY medium without IPTG for 24 h and harvested in the exponential growth phase. The values are the mean ± SD of three biological replicates. (E) Coomassie blue-stained SDS-PAGE gels with indicated purified proteins used for the *in vitro* enzyme activity and c-di-GMP binding assays shown in panels A, B and C.
(TIF)

S10 Fig Putative metallopeptidase RgsM is localized in the periplasm dependent on the N-terminal transmembrane segment. Detection of phosphatase activity is indicative for periplasmic localization. Protein fusions of full-length RgsM, its N-terminal portion (RgsM_1-66_) or periplasmic portion lacking the transmembrane α-helix (RgsM_60-646_) to PhoA_27-471_ were produced in Rm2011 and *E*. *coli* S17-1 grown on medium supplemented with PhoA substrate BCIP. Blue-staining of the agar cultures indicated periplasmic localization of PhoA mediated by the transmembrane α-helix of RgsM.
(TIF)

S11 Fig Overexpression of wild type and mutant *rgsM* in *S*. *meliloti* results in growth inhibition and cell morphology defects. (A) Growth of Rm2011, harboring the empty vector pWBT, pWBT-*rgsM* (wt^++^) or pWBT-*rgsM*_H510A_ (H510A^++^), and RgsM depletion strain Rm2011 *rgsM*^*dpl*^ in liquid TY or LB media in presence or absence of IPTG. OD_600_ is shown in logarithmic scale and error bars represent the standard deviation of three biological replicates. (B) DIC microscopy of cells from cultures shown in panel A after 24 h of growth in the indicated media. IPTG was added to all the cultures except for the Rm2011 *rgsM*^*dpl*^ culture. (C) Phase contrast and fluorescence microscopy images of cells, pulse-labeled with HADA for 3 min. *, compared to the two panels on the left, HADA fluorescence channel was intensity-adjusted to visualize the weak and dispersed signal in the RgsM-overproducing cells. Bars, 5 μm.
(TIF)

S12 Fig Growth inhibition and cell morphology defects upon *rgsM* overexpression in *S*. *meliloti* are dependent on the medium composition. (A) Rm2011, harboring either empty vector pWBT or pWBT-*rgsM* (*rgsM*^++^), grown on TY or LB agar with different NaCl and CaCl_2_ concentrations with or without added IPTG. (B) Growth of Rm2011 harboring either empty vector pWBT or pWBT-*rgsM* (*rgsM*^++^) in LB or in LB with the NaCl content increased to 300 mM. OD_600_ is shown in logarithmic scale and error bars represent the standard deviation of three biological replicates. (C) DIC microscopy of cells from cultures shown in panel B after 24 h of growth in the indicated media. Bars, 5 μm.
(TIF)

S13 Fig Overexpression of wild type and mutant *rgsM* in *E*. *coli* results in growth inhibition and cell morphology defects. (A) Phase contrast microscopy images of *E*. *coli* S17-1 harboring the empty vector pWBT, pWBT-*rgsM* (wt^++^) or pWBT-*rgsM*_H510A_ (H510A^++^). RgsM expression was induced with IPTG for 3 h in cultures grown in LB medium with or without NaCl. Lysed cells are indicated by arrowheads. Bars, 5 μm. (B) Growth of *E*. *coli* strains described in panel A on LB agar or LB agar without NaCl in presence or absence of IPTG. Serial dilutions of cell suspensions adjusted to OD_600_ of 1 are indicated.
(TIF)

S14 Fig HPLC elution profiles of reduced *S*. *meliloti* muropeptides obtained from wild type and RgsP and RgsM depletion strains. (A) Muropeptide profiles of RgsP and RgsM-sufficient cells (Rm2011, IPTG-supplemented Rm2011 *rgsP*^*dpl*^ and Rm2011 *rgsM*^*dpl*^), or RgsP and RgsM-depleted cells (Rm2011 *rgsP*^*dpl*^ and Rm2011 *rgsM*^*dpl*^, no IPTG added) grown in TY medium for 24 h. Most prominent alterations in muropeptide profiles are indicated. Representative muropeptide profiles of two independent biological replicates are shown. Bar, 100 mAU. (B) Quantification of muropeptides analyzed in panel A. Error bars represent the range of obtained mean values.
(TIF)

S15 Fig HPLC elution profile and mass spectrometric analysis of reduced *S*. *meliloti* muropeptides. (A) Muropeptide profiles of Rm2011 *rgsP*^*dpl*^ grown in TY medium without IPTG for 24 h. Peak numbers refer to muropeptides listed in S4 Table. Bar, 100 mAU. (B) MS/MS fragmentation spectra of 3–3 cross-linked TetraTri(Dap) and 3–4 cross-linked TetraTri. The mass difference of 89 amu indicates the loss of a terminal alanine residue, which is only possible with the 3–3 cross-linked TetraTri(Dap) where the terminal alanine is not part of the peptide cross-link.
(TIF)

S16 Fig HPLC elution profiles of reduced muropeptides obtained from *S*. *meliloti* and *E*. *coli* overexpressing *rgsM*. (A,B) Muropeptide profiles of Rm2011 (A) and *E*. *coli* S17-1 (B), harboring empty vector pWBT, pWBT-*rgsM* (wt^++^) or pWBT-*rgsM*_H510A_ (H510A^++^), grown in LB medium with added IPTG for 24 h (A) and 4 h (B). Representative muropeptide profiles of two independent biological replicates are shown. Bars, 100 mAU.
(TIF)

S17 Fig RgsM shows no PG hydrolase activity *in vitro*. (A,B) Muropeptide profiles of PG sacculi, isolated from strains described in S16A Fig and incubated with His_6_-RgsM_57-646_ in presence or absence of His_6_-RgsP_7TMR-DISMED2_ (A) and with His_6_-RgsM_260-646_ (B), to assay for RgsM PG endopeptidase activity. The control reactions contained no added protein. (C) PG binding ability of His_6_-RgsM_260-646_ was assessed in an *in vitro* binding assay with *S*. *meliloti* Rm2011 PG sacculi followed by SDS-PAGE and Coomassie blue staining. S, supernatant from the first centrifugation step. W, supernatant from the washing step. P, pellet. PG, peptidoglycan. M, molecular weight marker. Control reactions were performed in the absence of PG sacculi.
(TIF)

S18 Fig RgsP and RgsM homologs from related species are functional in *S*. *meliloti*. (A) Microscopy images of *R*. *etli* CFN42 *RHE_CH00976-egfp* and *A*. *tumefaciens* C58 *Atu0784*-*egfp* cells (carrying gene fusions at the native chromosomal location) pulse-labeled with HADA for 3.5 and 2.5 min, respectively. Arrowheads indicate the mid-cell region of pre-divisional cells. Bars, 5 μm. (B) Complementation of the growth defect of RgsP depletion strain Rm2011 *rgsP*^*dpl*^ and RgsM depletion strain Rm2011 *rgsM*^*dpl*^ by ectopic expression of *rgsP*_Sm_, *rgsP*_Re_ or *rgsP*_At_, and *rgsM*_Sm_, *rgsM*_Re_ or *rgsM*_At_, respectively, from vector pR-P_*tau*_ in presence or absence of taurine. Relative growth was calculated as a ratio of OD_600_ values obtained for cultures induced for expression of the chromosomally encoded *rgsP* and *rgsM* (grown with IPTG) to the OD_600_ values of cultures non-induced for expression of the wild type *rgsP* and *rgsM* alleles (grown without IPTG), respectively, 42 h after inoculation. Error bars represent the standard deviation of three biological replicates. (C) Microscopy images of *S*. *meliloti* strains indicated in panel B grown for 24 h with or without taurine and in absence of IPTG. Bars, 5 μm.
(TIF)

S1 Table Co-immunoprecipitation revealed putative interaction partners of RgsP (SMc00074). Proteins were identified by mass spectrometry. Identified proteins for Rm2011 harboring the empty vector pWBT were removed from the obtained list of candidate interaction partners. MW, molecular weight; AAs, number of amino acids; PSMs, peptide-spectrum matches.
(PDF)

S2 Table Co-immunoprecipitation revealed putative interaction partners of RgsM (SMc02432). Proteins were identified by mass spectrometry. Identified proteins for Rm2011 harboring the empty vector pWBT were removed from the obtained list of candidate interaction partners. MW, molecular weight; AAs, number of amino acids; PSMs, peptide-spectrum matches.
(PDF)

S3 Table RgsP and RgsM are mainly conserved in related species from the order Rhizobiales. BlastP search for RgsP and RgsM sequences revealed homologous proteins in the alphaproteobacterial order Rhizobiales (examples are listed) as well as in one species from the Gammaproteobacteria. Multiple sequence alignment of RgsP homologs revealed conserved GGDEF and EAL domain motifs [12,16] required for DGC and PDE activity, respectively. Variations of the consensus sequence of GGDEF and EAL active sites are colored in red. Multiple sequence alignment of RgsM homologs revealed conserved zinc ion coordination motifs previously identified for *Staphylococcus aureus* LytM [28–30]. *, 90% or more of the query sequence annealed.
(PDF)

S4 Table Names and mass spectrometry data for the muropeptide fractions shown in S15A Fig. (PDF)

S5 Table Strains and plasmids used in this study. (PDF)

S6 Table Primers used in this study. (PDF)

S7 Table Numerical data. (XLSX)
