# Supplementary figures and images for: Seven-transmembrane receptor protein RgsP and cell wall-binding protein RgsM promote unipolar growth in Rhizobiales
Source: PLoS Genet. 2018 Aug 13;14(8):e1007594. doi: 10.1371/journal.pgen.1007594 (PMC6107284; doi:10.1371/journal.pgen.1007594)

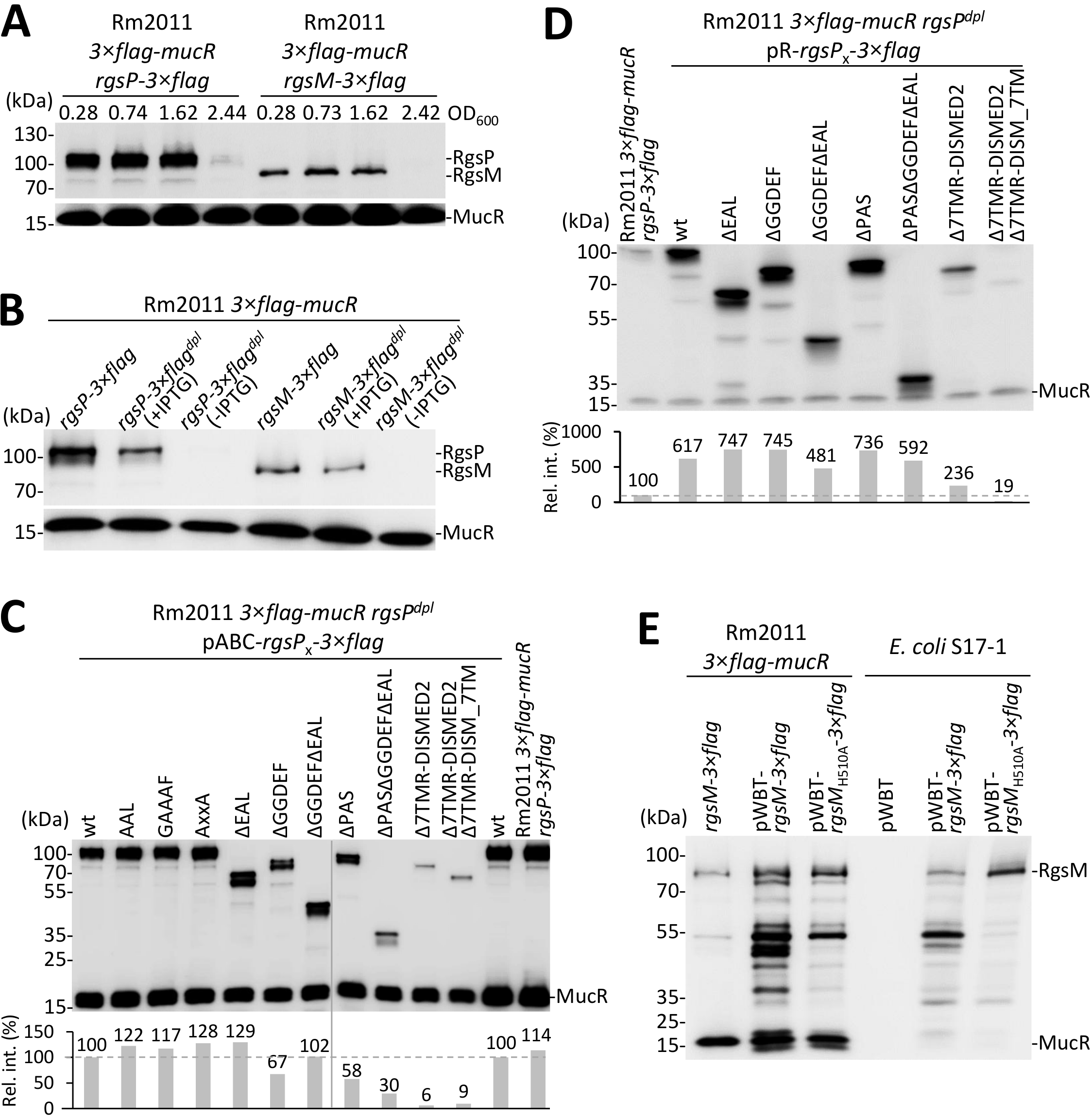

Supplement: S1 Fig — 3×FLAG-MucR produced from the native genomic location was used as a loading control. (A) Growth stage-dependent abundance of RgsP and RgsM. Cells producing RgsP-3×FLAG or RgsM-3×FLAG from the native genomic location were harvested at indicated culture OD600. (B) Conditional depletion of RgsP and RgsM. Analyzed cells produced RgsP-3×FLAG or RgsM-3×FLAG from the native genomic location. In depletion strains (‘dpl’), the corresponding gene fusions were placed under control of IPTG-inducible promoters. These strains were grown in presence and absence of added IPTG. (C,D) Accumulation of indicated RgsP variants, produced from P*rgsP on a single-copy plasmid (C) or from Psyn on a low-copy plasmid (D). Mean gray values of bands corresponding to 3×FLAG-tagged RgsP variants are shown relative to RgsPwt-3×FLAG produced from P*rgsP (C) or relative to RgsPwt-3×FLAG produced from the native genomic location (D). ‘x’ denotes the respective RgsP variant. (E) Accumulation of RgsM variants in S. meliloti and E. coli. Indicated strains were grown in presence of IPTG either in LB for 24 h (S. meliloti) or in LB lacking NaCl for 3 h (E. coli). (TIF) [file pgen.1007594.s001.tif]

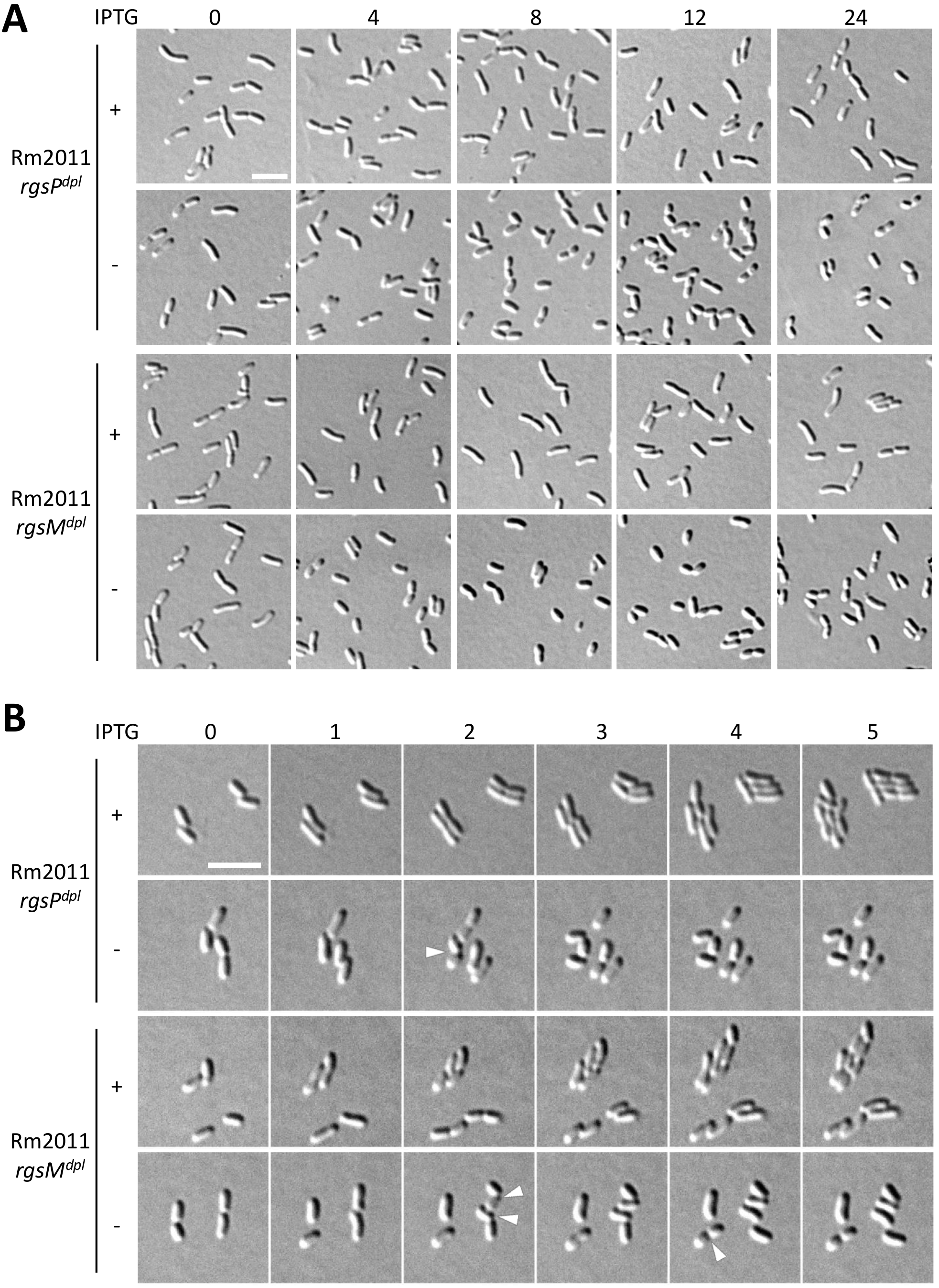

Supplement: S2 Fig — (A) DIC microscopy images of RgsP and RgsM depletion strains grown in liquid TY medium with and without added IPTG at indicated time points. Time is given in hours. (B) Time-lapse DIC microscopy of RgsP and RgsM depletion strains, previously grown in TY medium without IPTG for 6 h and 4 h, respectively, and placed on TY agarose pads with and without added IPTG. Arrow heads indicate cells showing no substantial cell elongation prior to visible septum constriction. Time is given in hours. Bars, 5 μm. (TIF) [file pgen.1007594.s002.tif]

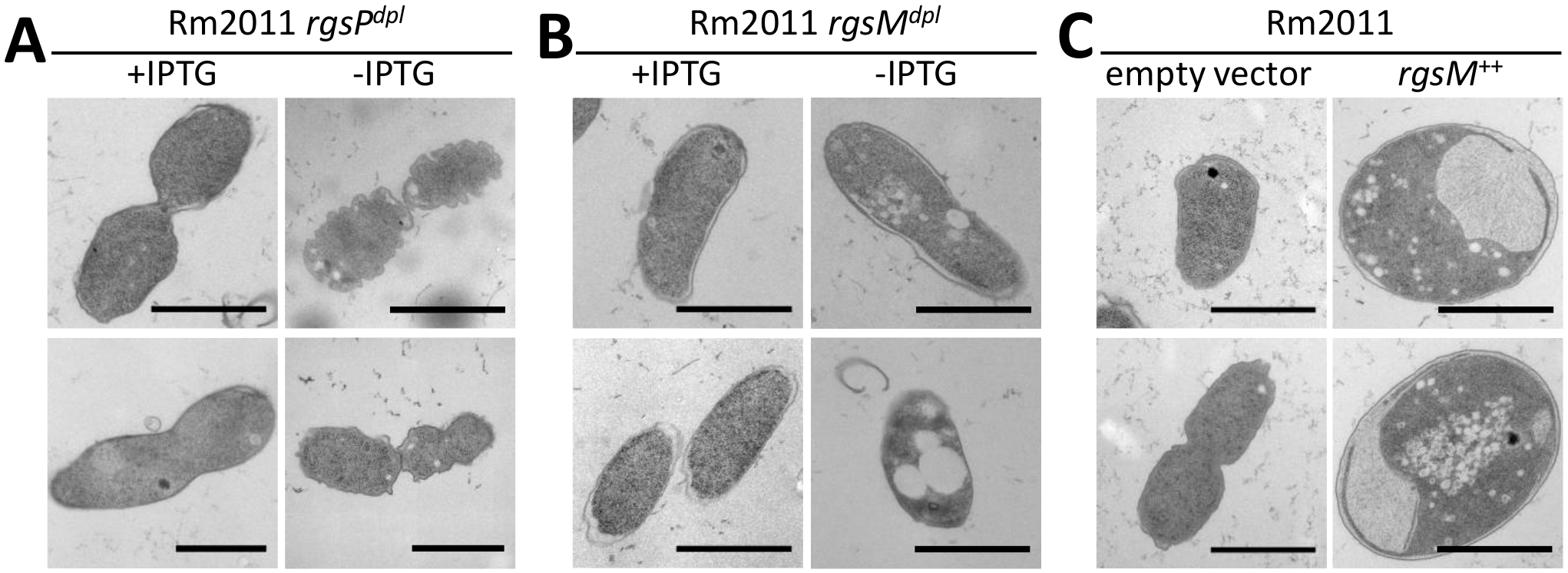

Supplement: S3 Fig — (A,B) Depletion strains were grown in TY with and without added IPTG. (C) Rm2011, harboring either empty vector pWBT or rgsM overexpression plasmid pWBT-rgsM (rgsM++), grown in LB with IPTG. Bars, 1 μm. (TIF) [file pgen.1007594.s003.tif]

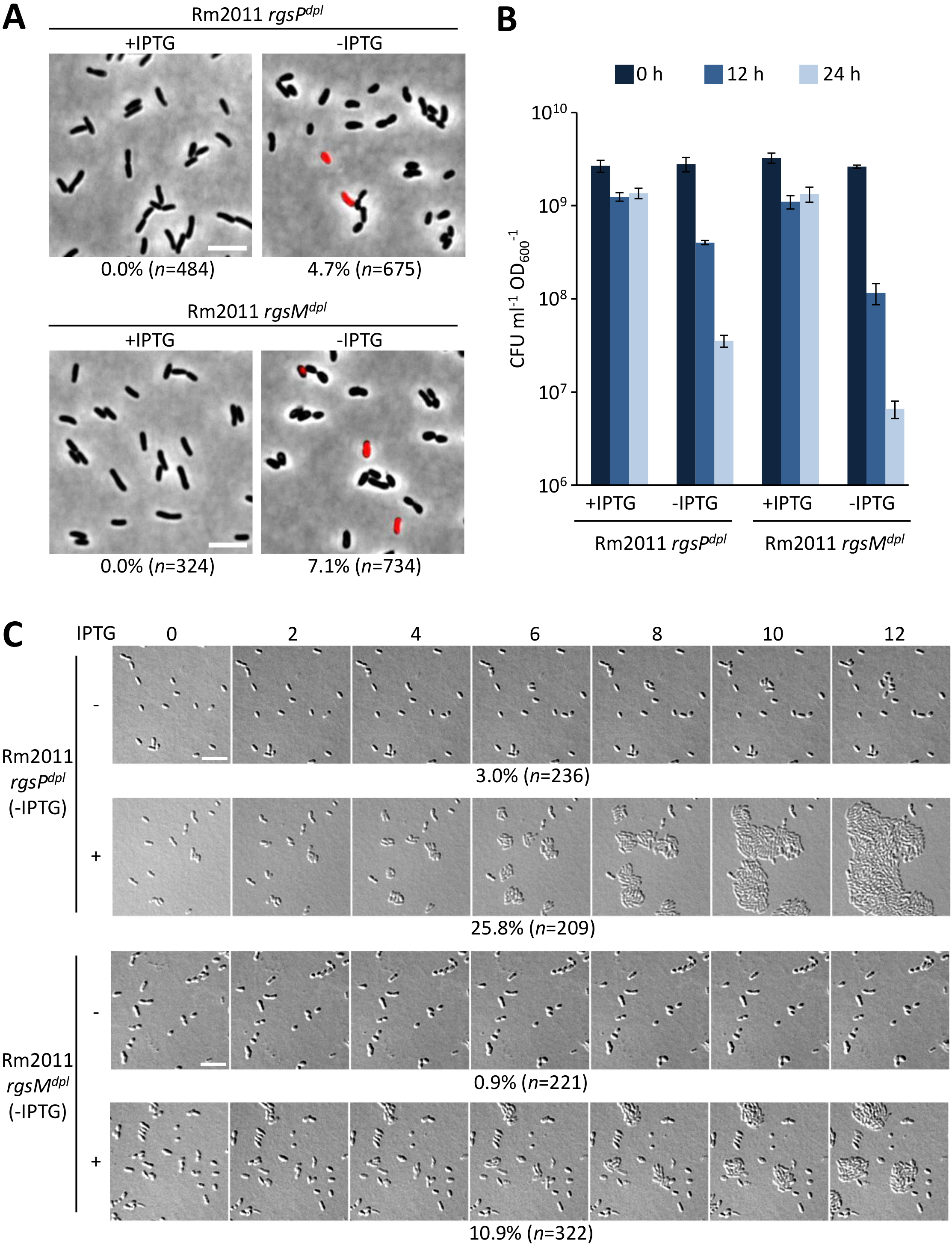

Supplement: S4 Fig — (A) RgsP and RgsM depletion strains were grown in TY medium with and without added IPTG for 24 h. Cells were stained with propidium iodide and analyzed by fluorescence microscopy. The proportion of red fluorescent cells is indicated. n, total number of cells considered for statistical analysis. Bars, 5 μm. (B) After cultivation of cells under depletion (TY medium without IPTG) or non-depletion conditions (TY medium with IPTG) for 0, 12 and 24 h, the number of colony forming units (CFU) was determined by plating cells on TY medium with IPTG, followed by 72 h incubation. Error bars represent the standard deviation of three biological replicates. (C) Time-lapse DIC microscopy of RgsP- and RgsM-depleted cells, previously grown in TY medium without IPTG for 24 h, followed by incubation on TY agarose pads with or without IPTG. Time is given in hours. The proportion of dividing cells is indicated. n, total number of cells considered for statistical analysis. Bars, 5 μm. (TIF) [file pgen.1007594.s004.tif]

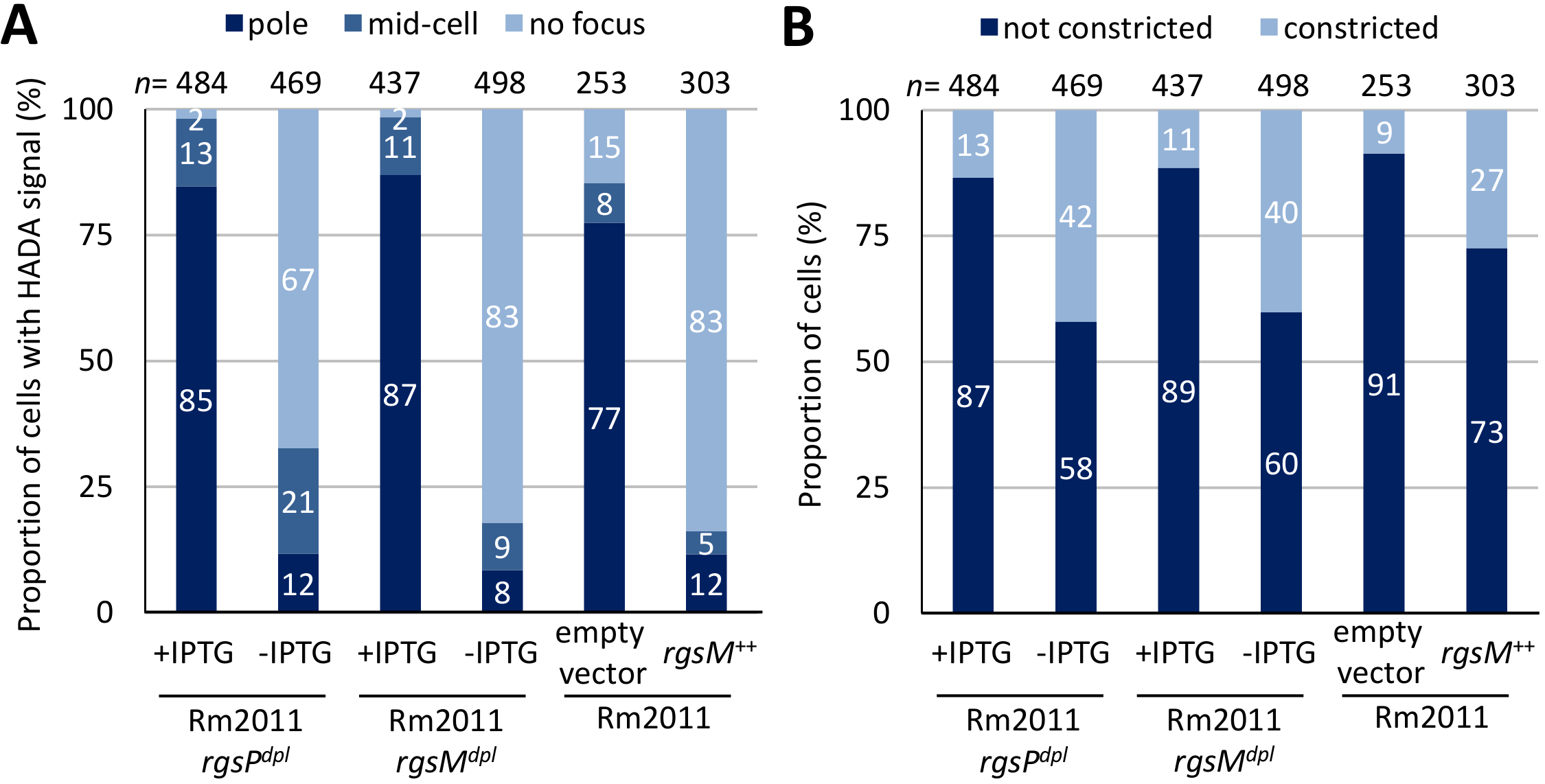

Supplement: S5 Fig — Depletion strains were grown in TY medium with and without added IPTG and Rm2011, harboring either empty vector pWBT or rgsM overexpression plasmid pWBT-rgsM (rgsM++), was grown in LB medium with IPTG for 24 h. (A) Relative proportion of cells analyzed by fluorescence microscopy and displaying HADA signal at one cell pole (pole), at mid-cell (mid-cell), or no HADA focus (no focus). (B) Bacteria described in panel A were classified into cells with and without visible septum constriction. n, number of cells considered for statistical analysis. (TIF) [file pgen.1007594.s005.tif]

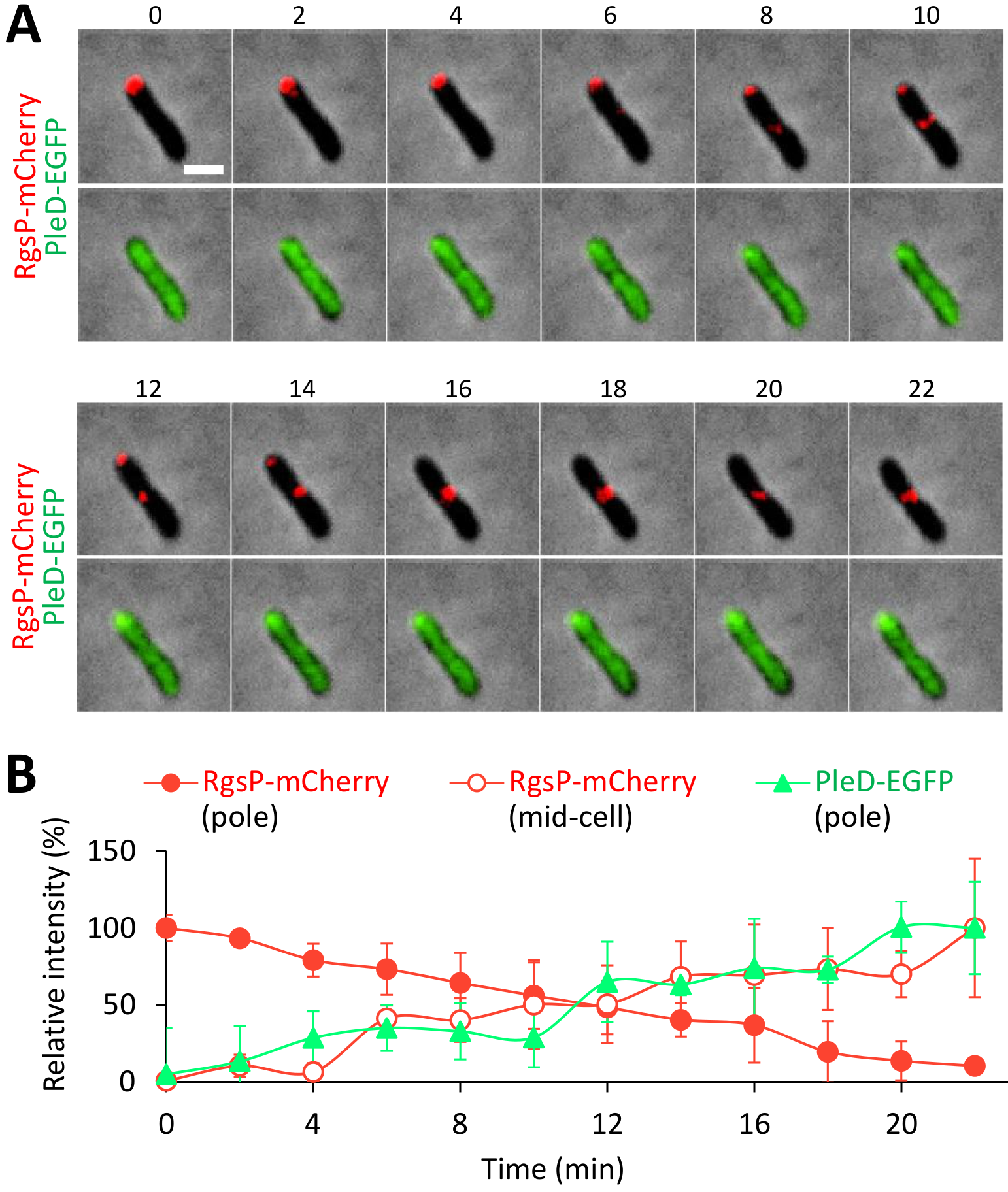

Supplement: S6 Fig — (A) Time-lapse microscopy of Rm2011 rgsP-mCherry pleD-egfp strain, carrying the gene fusions at native genomic locations. Time is shown in minutes. Bar, 1 μm. (B) Accumulation of PleD-EGFP at the new pole relative to relocation of RgsP-mCherry signal from pole to the mid-cell was monitored by quantification of fluorescence signals in the respective cell areas. Error bars represent the standard deviation of three biological replicates which included the time-lapse microscopy images shown in panel A and two additional biological replicates. (TIF) [file pgen.1007594.s006.tif]

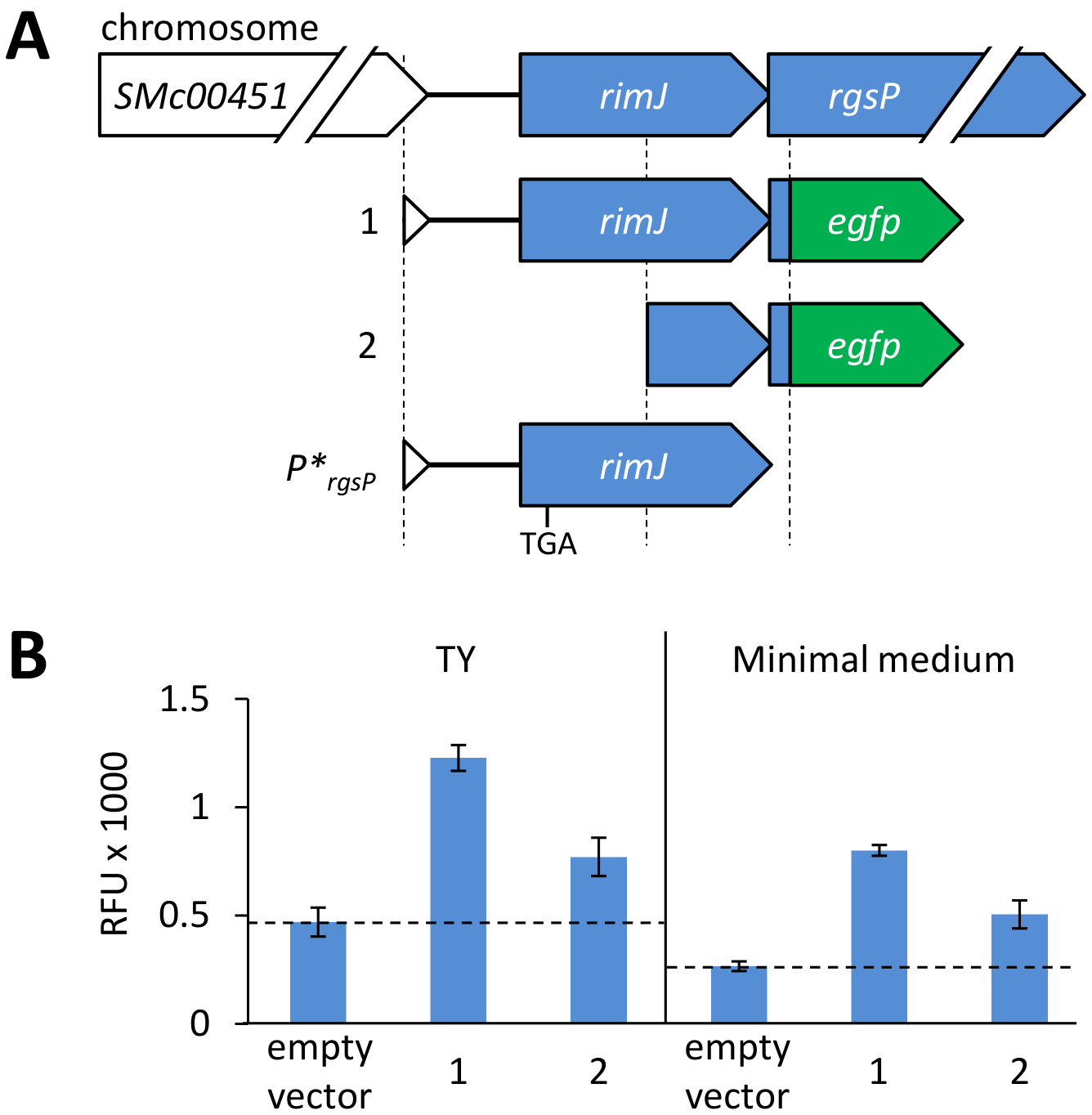

Supplement: S7 Fig — P*rgsP consists of the rimJ promoter region followed by rimJ coding sequence with TGA stop codon introduced at nucleotide position 64. (A) rgsP upstream regions including the rimJ promoter and the whole rimJ coding sequence (upstream region 1), or only partial rimJ coding sequence (upstream region 2), followed by the first three codons of rgsP, translationally fused to egfp to estimate the promoter activity of these regions. (B) Normalized EGFP fluorescence of Rm2011, harboring medium-copy plasmids carrying the translational fusions depicted in panel A, grown in TY and minimal media. Error bars represent the standard deviation of three biological replicates. (TIF) [file pgen.1007594.s007.tif]

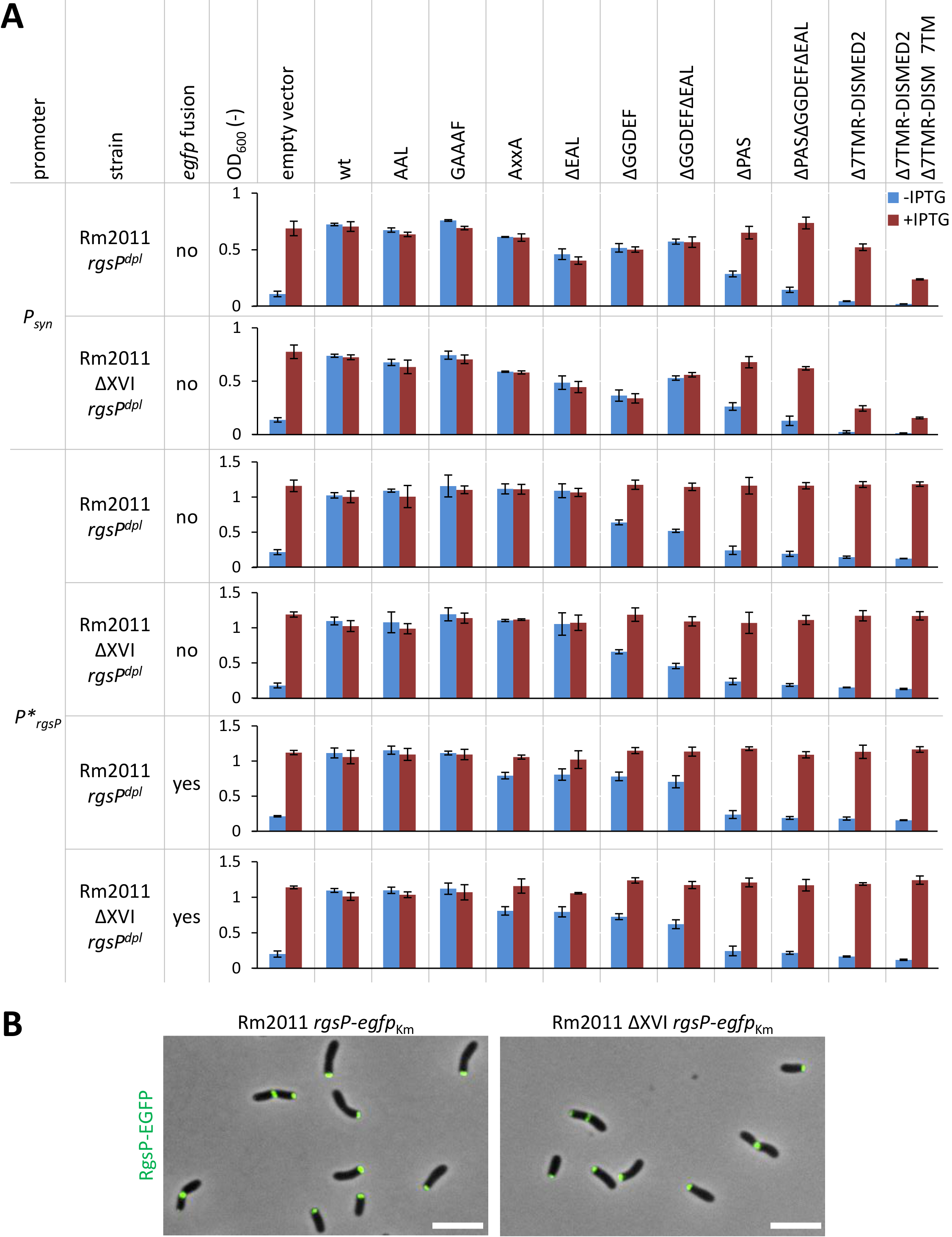

Supplement: S8 Fig — (A) Recorded growth of Rm2011 rgsPdpl and c-di-GMP0 strain Rm2011 ΔXVI rgsPdpl, ectopically expressing non-tagged and egfp-tagged rgsP variants from either P*rgsP (vector pABC2S-mob, transcription strength similar to the native level) or Psyn (vector pR_egfp, elevated transcription strength compared to the native levels), grown in absence or presence of IPTG. OD600 values were recorded after 24 h of growth. Error bars represent the standard deviation of three biological replicates. (B) Subcellular localization of RgsP-EGFP in Rm2011 and Rm2011 ΔXVI. Exponentially growing cells were analyzed by fluorescence microscopy. Bars, 5 μm. (TIF) [file pgen.1007594.s008.tif]

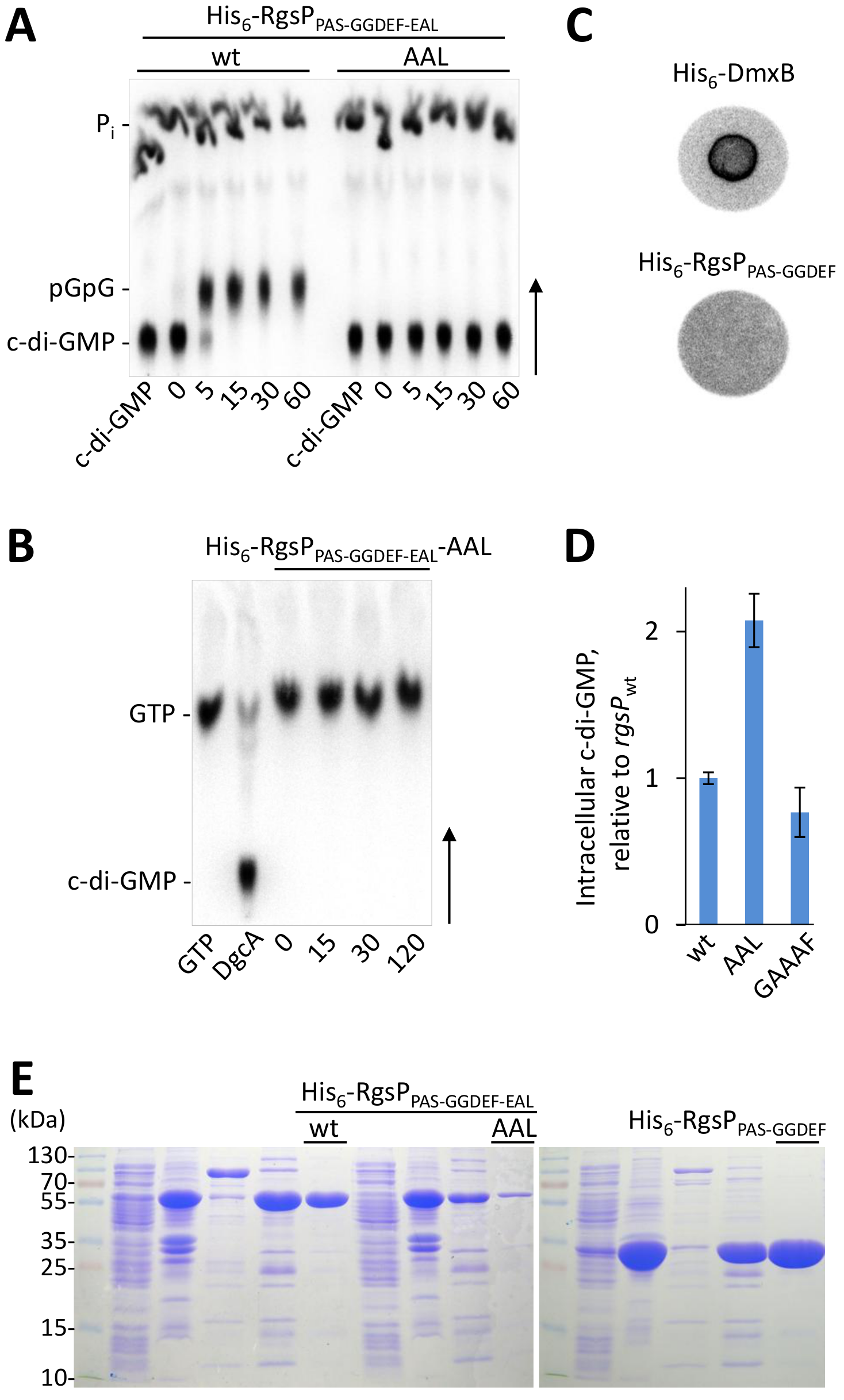

Supplement: S9 Fig — (A,B) Thin-layer chromatograms of PDE reactions with purified His6-RgsPPAS-GGDEF-EAL or His6-RgsPPAS-GGDEF-EAL-AAL and [α-32P]-c-di-GMP (A) and DGC reactions with purified His6-RgsPPAS-GGDEF-EAL-AAL or positive control His6-DgcA and [α-32P]-GTP (B). Incubation time is given in minutes. Arrows indicate the direction of mobile phase migration. Pi, inorganic phosphate; pGpG, linear di-GMP. (C) DRaCALA with purified His6-RgsPPAS-GGDEF and His6-DmxB as positive control. (D) c-di-GMP content of Rm2011 rgsPdpl, expressing the indicated rgsP variants from P*rgsP, grown in TY medium without IPTG for 24 h and harvested in the exponential growth phase. The values are the mean ± SD of three biological replicates. (E) Coomassie blue-stained SDS-PAGE gels with indicated purified proteins used for the in vitro enzyme activity and c-di-GMP binding assays shown in panels A, B and C. (TIF) [file pgen.1007594.s009.tif]

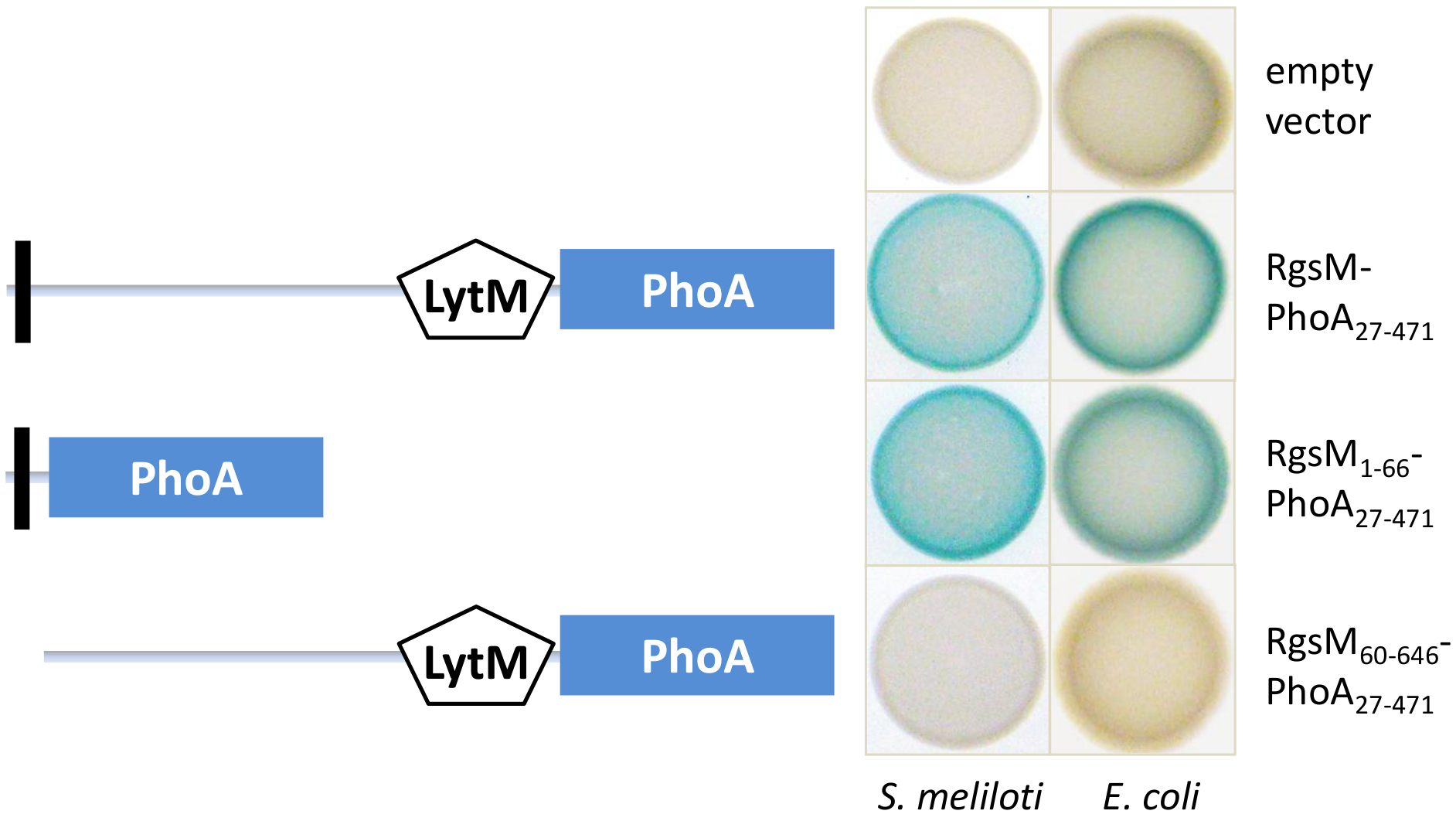

Supplement: S10 Fig — Detection of phosphatase activity is indicative for periplasmic localization. Protein fusions of full-length RgsM, its N-terminal portion (RgsM1-66) or periplasmic portion lacking the transmembrane α-helix (RgsM60-646) to PhoA27-471 were produced in Rm2011 and E. coli S17-1 grown on medium supplemented with PhoA substrate BCIP. Blue-staining of the agar cultures indicated periplasmic localization of PhoA mediated by the transmembrane α-helix of RgsM. (TIF) [file pgen.1007594.s010.tif]

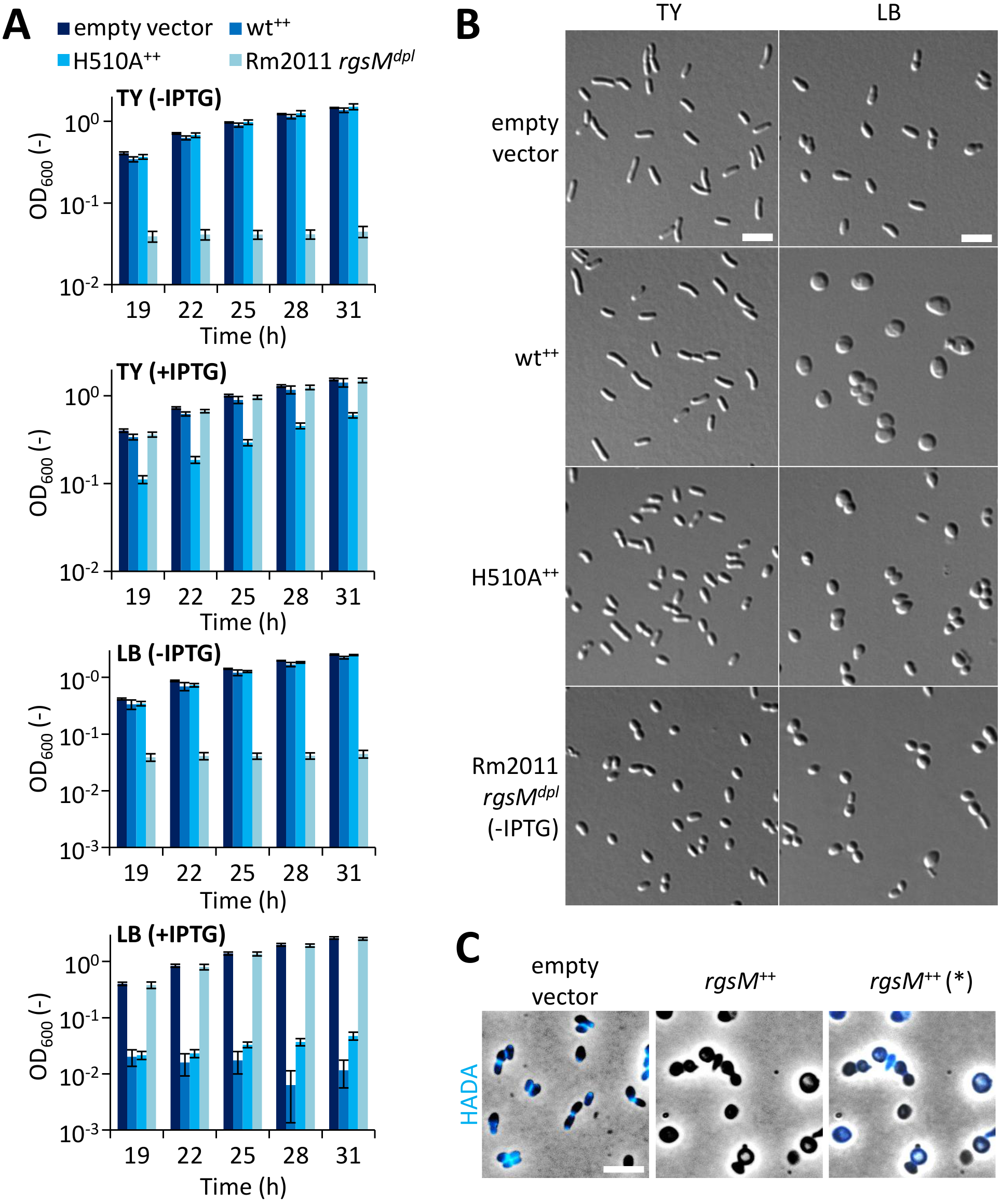

Supplement: S11 Fig — (A) Growth of Rm2011, harboring the empty vector pWBT, pWBT-rgsM (wt++) or pWBT-rgsMH510A (H510A++), and RgsM depletion strain Rm2011 rgsMdpl in liquid TY or LB media in presence or absence of IPTG. OD600 is shown in logarithmic scale and error bars represent the standard deviation of three biological replicates. (B) DIC microscopy of cells from cultures shown in panel A after 24 h of growth in the indicated media. IPTG was added to all the cultures except for the Rm2011 rgsMdpl culture. (C) Phase contrast and fluorescence microscopy images of cells, pulse-labeled with HADA for 3 min. *, compared to the two panels on the left, HADA fluorescence channel was intensity-adjusted to visualize the weak and dispersed signal in the RgsM-overproducing cells. Bars, 5 μm. (TIF) [file pgen.1007594.s011.tif]

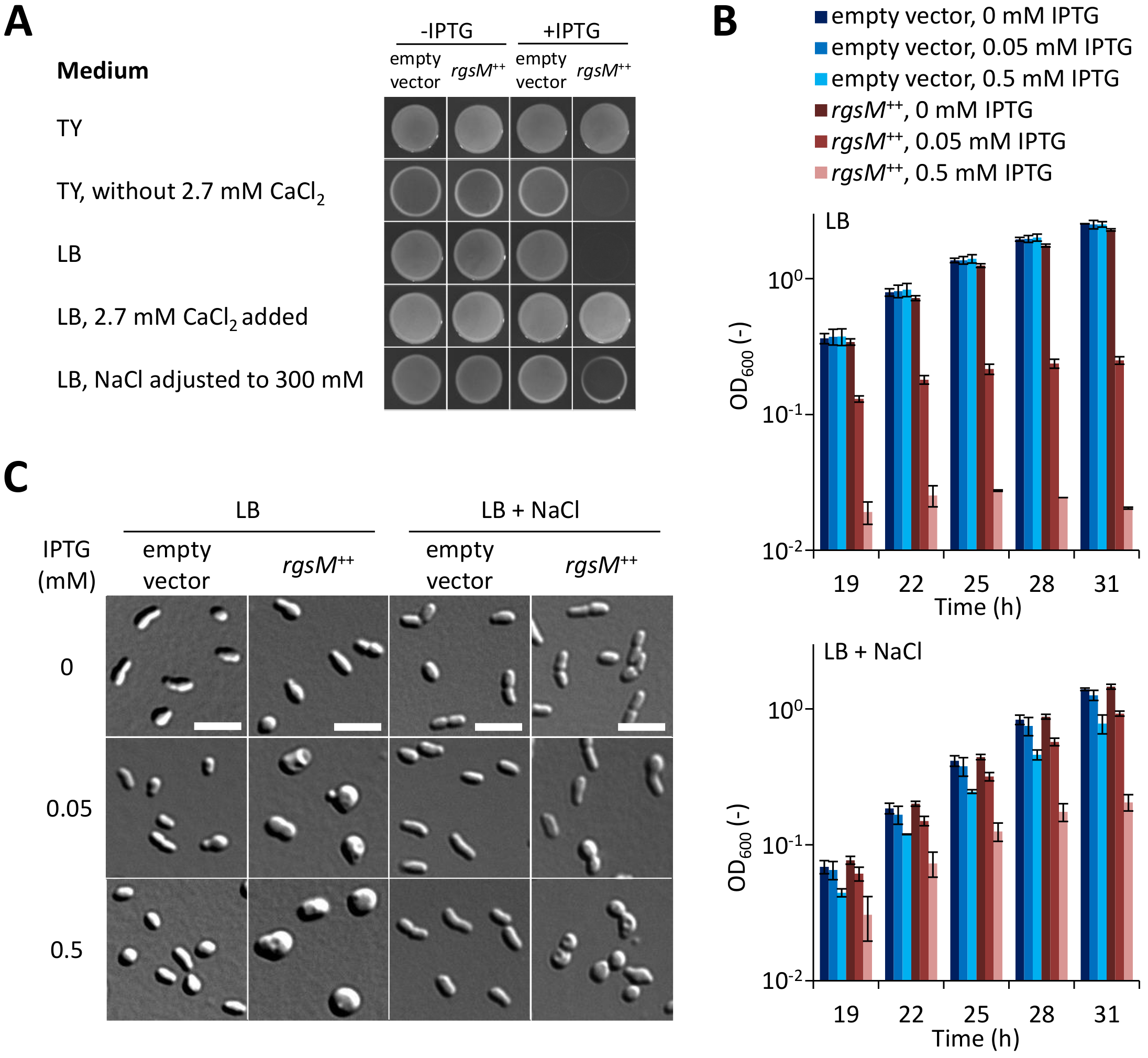

Supplement: S12 Fig — (A) Rm2011, harboring either empty vector pWBT or pWBT-rgsM (rgsM++), grown on TY or LB agar with different NaCl and CaCl2 concentrations with or without added IPTG. (B) Growth of Rm2011 harboring either empty vector pWBT or pWBT-rgsM (rgsM++) in LB or in LB with the NaCl content increased to 300 mM. OD600 is shown in logarithmic scale and error bars represent the standard deviation of three biological replicates. (C) DIC microscopy of cells from cultures shown in panel B after 24 h of growth in the indicated media. Bars, 5 μm. (TIF) [file pgen.1007594.s012.tif]

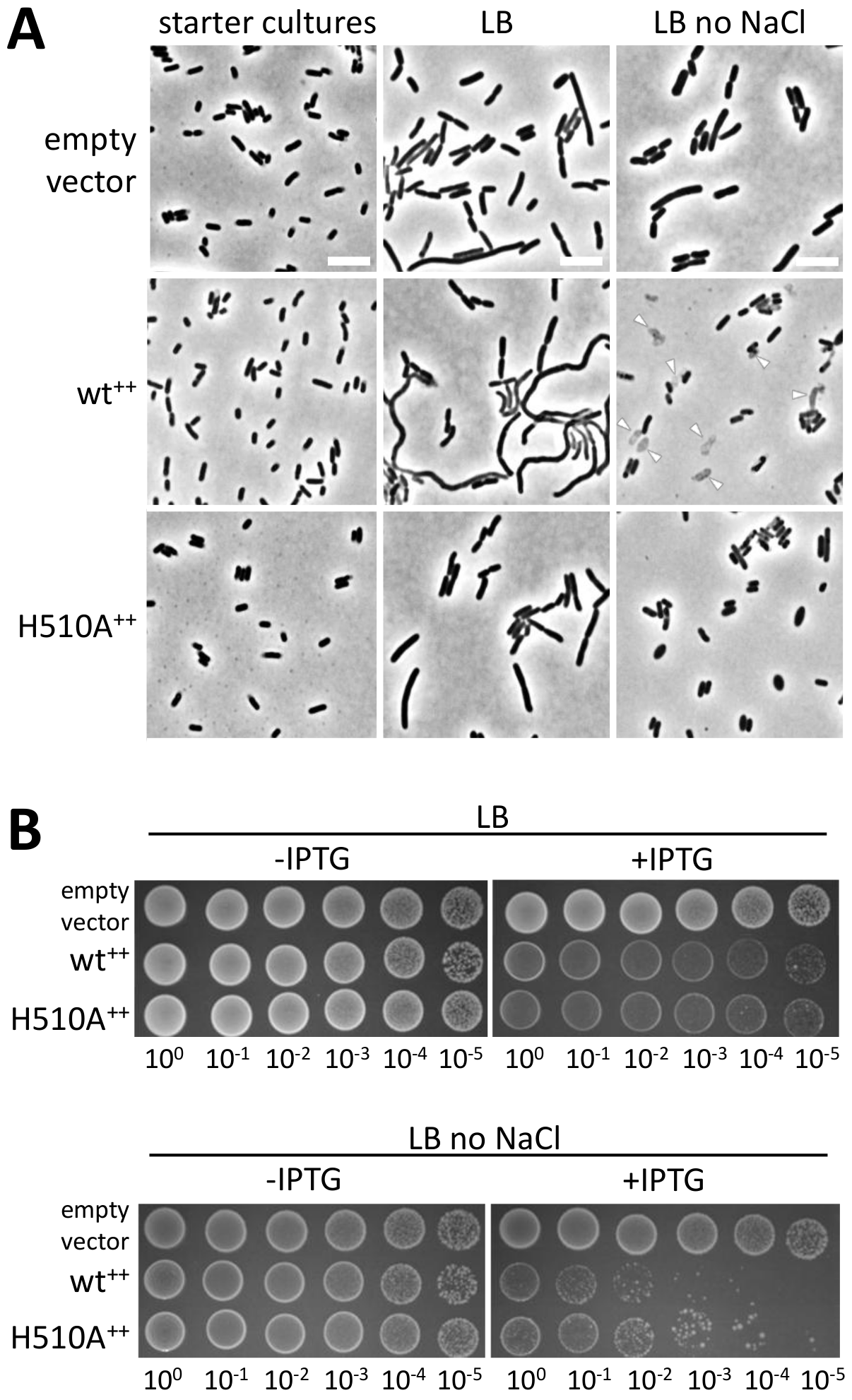

Supplement: S13 Fig — (A) Phase contrast microscopy images of E. coli S17-1 harboring the empty vector pWBT, pWBT-rgsM (wt++) or pWBT-rgsMH510A (H510A++). RgsM expression was induced with IPTG for 3 h in cultures grown in LB medium with or without NaCl. Lysed cells are indicated by arrowheads. Bars, 5 μm. (B) Growth of E. coli strains described in panel A on LB agar or LB agar without NaCl in presence or absence of IPTG. Serial dilutions of cell suspensions adjusted to OD600 of 1 are indicated. (TIF) [file pgen.1007594.s013.tif]

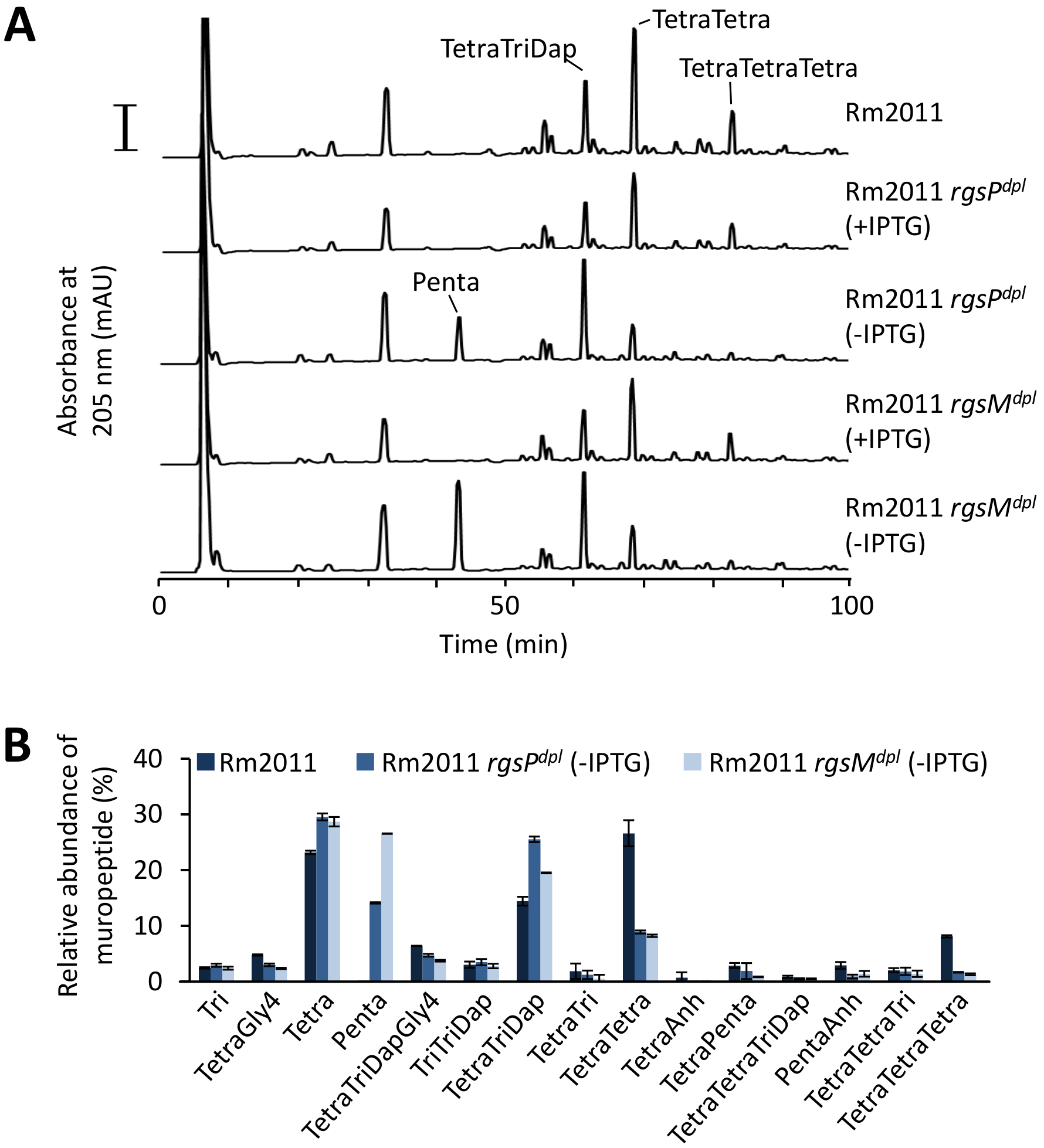

Supplement: S14 Fig — (A) Muropeptide profiles of RgsP and RgsM-sufficient cells (Rm2011, IPTG-supplemented Rm2011 rgsPdpl and Rm2011 rgsMdpl), or RgsP and RgsM-depleted cells (Rm2011 rgsPdpl and Rm2011 rgsMdpl, no IPTG added) grown in TY medium for 24 h. Most prominent alterations in muropeptide profiles are indicated. Representative muropeptide profiles of two independent biological replicates are shown. Bar, 100 mAU. (B) Quantification of muropeptides analyzed in panel A. Error bars represent the range of obtained mean values. (TIF) [file pgen.1007594.s014.tif]

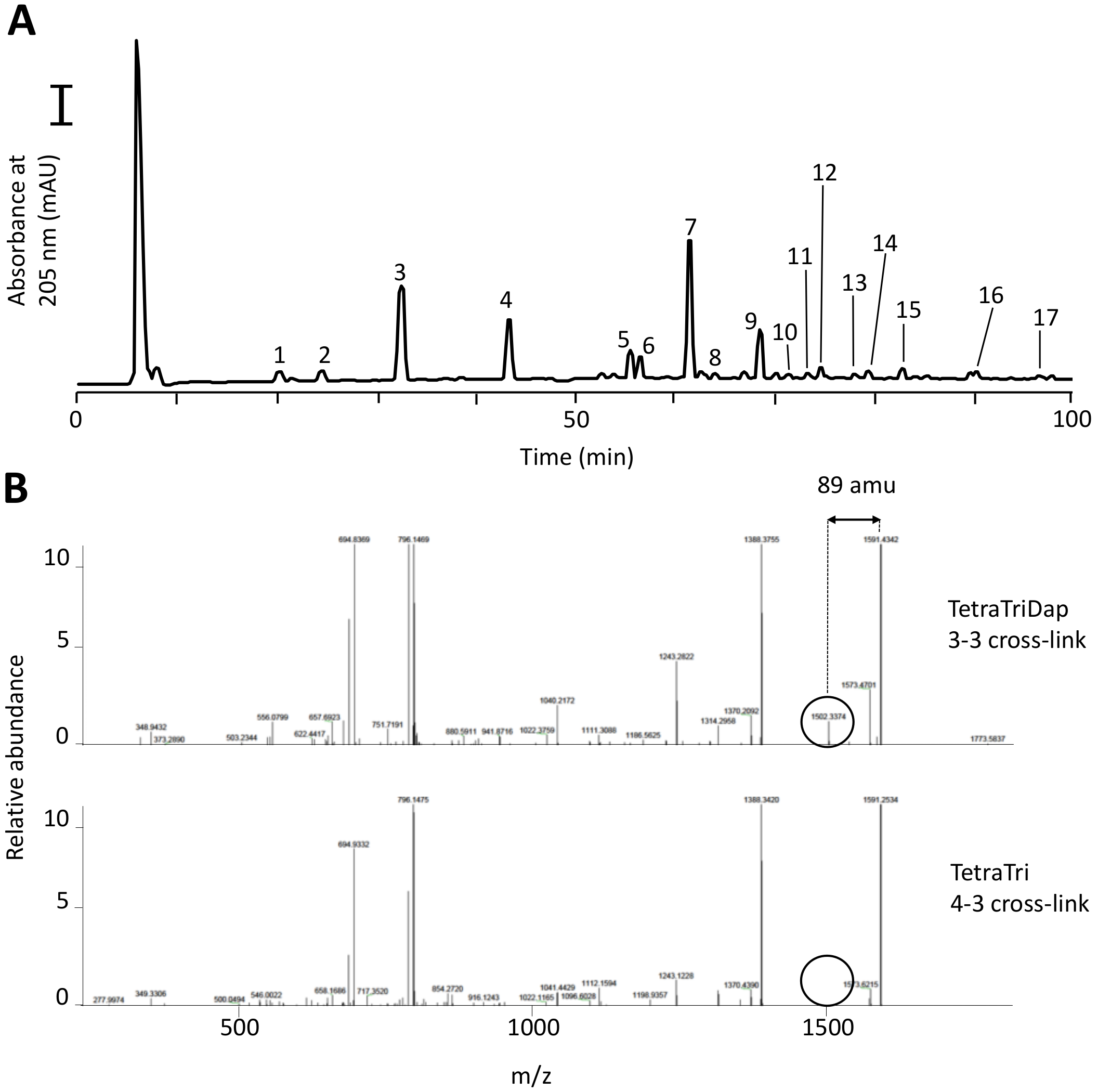

Supplement: S15 Fig — (A) Muropeptide profiles of Rm2011 rgsPdpl grown in TY medium without IPTG for 24 h. Peak numbers refer to muropeptides listed in S4 Table. Bar, 100 mAU. (B) MS/MS fragmentation spectra of 3–3 cross-linked TetraTri(Dap) and 3–4 cross-linked TetraTri. The mass difference of 89 amu indicates the loss of a terminal alanine residue, which is only possible with the 3–3 cross-linked TetraTri(Dap) where the terminal alanine is not part of the peptide cross-link. (TIF) [file pgen.1007594.s015.tif]

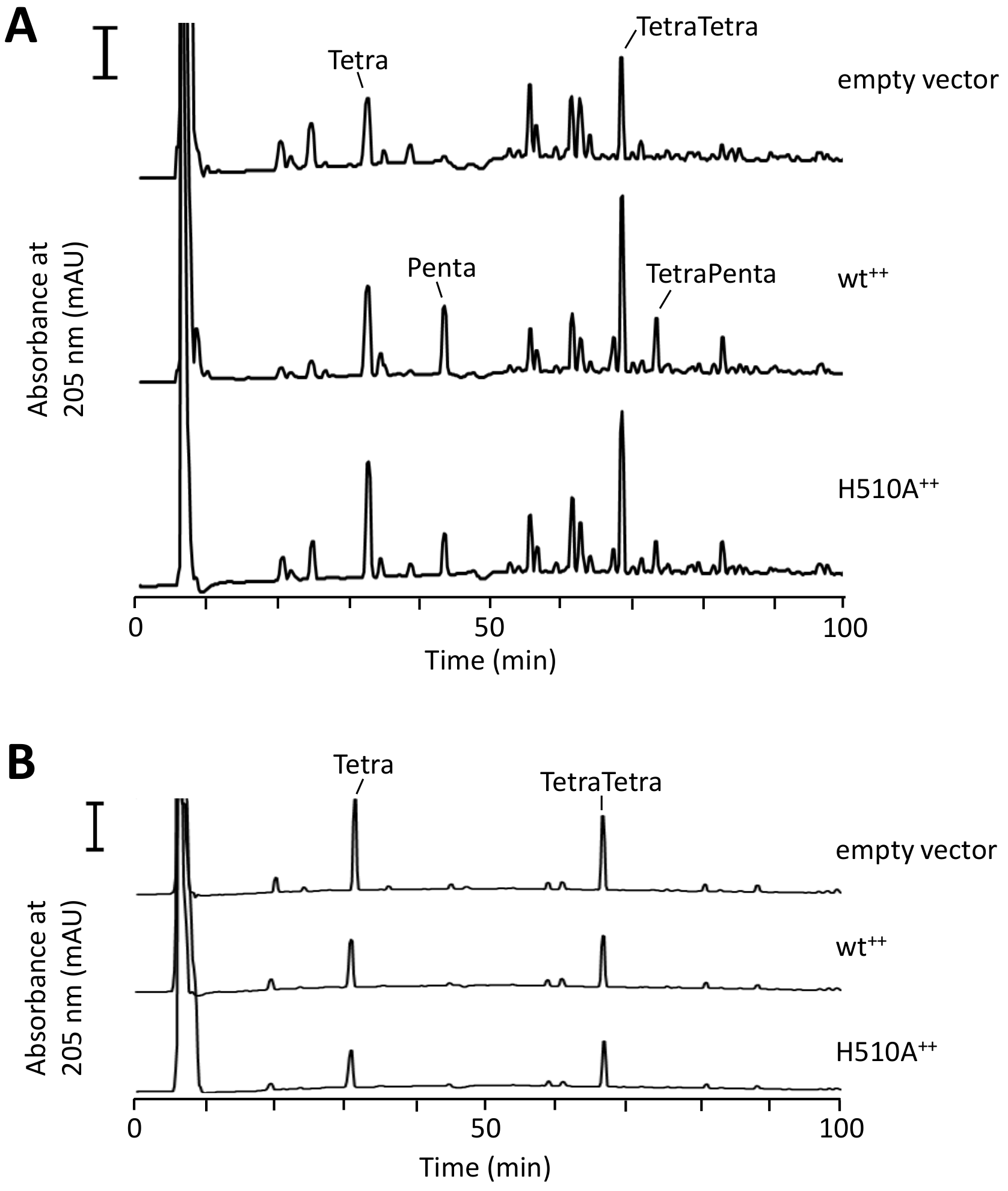

Supplement: S16 Fig — (A,B) Muropeptide profiles of Rm2011 (A) and E. coli S17-1 (B), harboring empty vector pWBT, pWBT-rgsM (wt++) or pWBT-rgsMH510A (H510A++), grown in LB medium with added IPTG for 24 h (A) and 4 h (B). Representative muropeptide profiles of two independent biological replicates are shown. Bars, 100 mAU. (TIF) [file pgen.1007594.s016.tif]

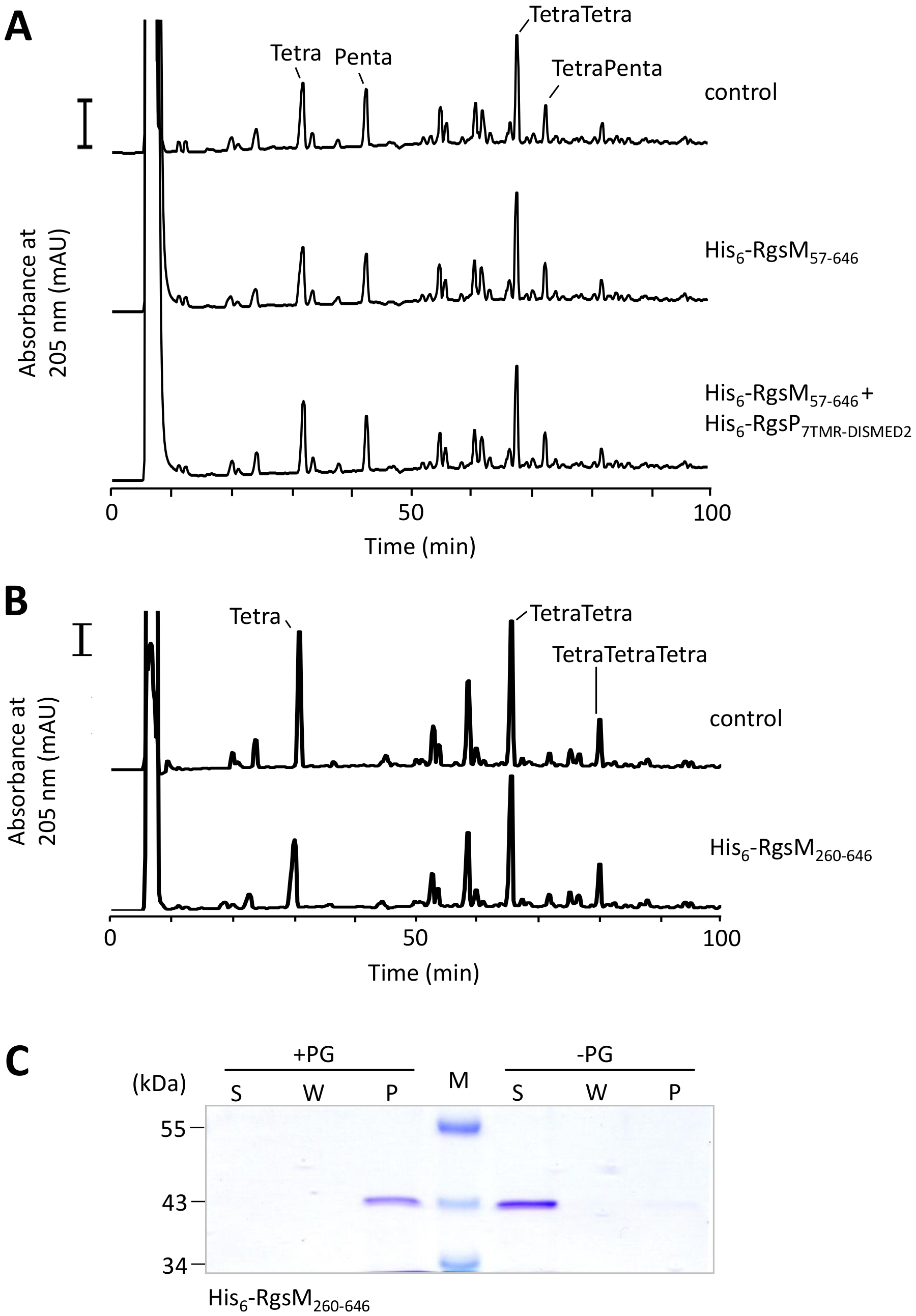

Supplement: S17 Fig — (A,B) Muropeptide profiles of PG sacculi, isolated from strains described in S16A Fig and incubated with His6-RgsM57-646 in presence or absence of His6-RgsP7TMR-DISMED2 (A) and with His6-RgsM260-646 (B), to assay for RgsM PG endopeptidase activity. The control reactions contained no added protein. (C) PG binding ability of His6-RgsM260-646 was assessed in an in vitro binding assay with S. meliloti Rm2011 PG sacculi followed by SDS-PAGE and Coomassie blue staining. S, supernatant from the first centrifugation step. W, supernatant from the washing step. P, pellet. PG, peptidoglycan. M, molecular weight marker. Control reactions were performed in the absence of PG sacculi. (TIF) [file pgen.1007594.s017.tif]

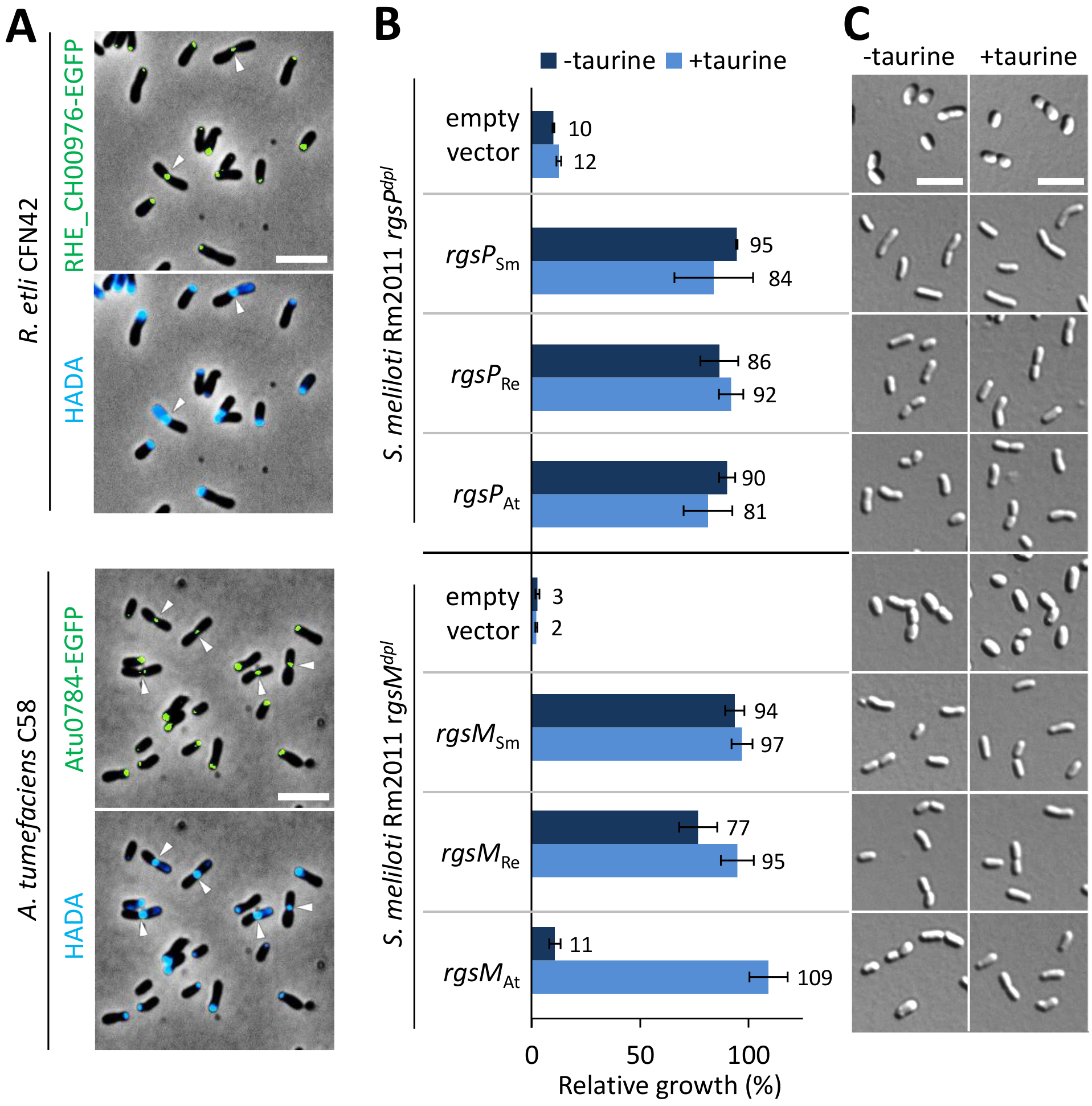

Supplement: S18 Fig — (A) Microscopy images of R. etli CFN42 RHE_CH00976-egfp and A. tumefaciens C58 Atu0784-egfp cells (carrying gene fusions at the native chromosomal location) pulse-labeled with HADA for 3.5 and 2.5 min, respectively. Arrowheads indicate the mid-cell region of pre-divisional cells. Bars, 5 μm. (B) Complementation of the growth defect of RgsP depletion strain Rm2011 rgsPdpl and RgsM depletion strain Rm2011 rgsMdpl by ectopic expression of rgsPSm, rgsPRe or rgsPAt, and rgsMSm, rgsMRe or rgsMAt, respectively, from vector pR-Ptau in presence or absence of taurine. Relative growth was calculated as a ratio of OD600 values obtained for cultures induced for expression of the chromosomally encoded rgsP and rgsM (grown with IPTG) to the OD600 values of cultures non-induced for expression of the wild type rgsP and rgsM alleles (grown without IPTG), respectively, 42 h after inoculation. Error bars represent the standard deviation of three biological replicates. (C) Microscopy images of S. meliloti strains indicated in panel B grown for 24 h with or without taurine and in absence of IPTG. Bars, 5 μm. (TIF) [file pgen.1007594.s018.tif]
