# Supplementary material for: Seven-transmembrane receptor protein RgsP and cell wall-binding protein RgsM promote unipolar growth in Rhizobiales
Source: PLoS Genet. 2018 Aug 13;14(8):e1007594. doi: 10.1371/journal.pgen.1007594 (PMC6107284; doi:10.1371/journal.pgen.1007594)
Supplement: S1 Table — Proteins were identified by mass spectrometry. Identified proteins for Rm2011 harboring the empty vector pWBT were removed from the obtained list of candidate interaction partners. MW, molecular weight; AAs, number of amino acids; PSMs, peptide-spectrum matches. (PDF) [file pgen.1007594.s019.pdf]

S1 Table. Co-immunoprecipitation revealed putative interaction partners of RgsP (SMc00074).

Accession	Description	Coverage	Unique Peptides	Peptides	PSMs	AAs	MW [kDa]
15964671	transmembrane signal peptide protein SMc00074 (RgsP)	36.29	22	27	42	970	107.2
15966360	hypothetical protein SMc02432 (RgsM)	54.64	18	22	25	646	69.7
15966480	hypothetical protein SMc00644	26.16	8	10	12	753	80.0
15964167	small heat shock protein	67.83	5	6	9	143	15.9
15965540;Q92PG9	30S ribosomal protein S4	36.59	5	5	9	205	23.5
15965409;Q92PS4	glucosamine--fructose-6-phosphate aminotransferase	23.36	5	8	14	608	65.8
15965119;Q92QG0	50S ribosomal protein L14	47.54	4	5	8	122	13.4
15965121;Q92QF8	50S ribosomal protein L5	32.43	4	4	8	185	20.9
2580515	citrate synthase	28.44	4	8	12	429	47.9
15965337	peptidyl-prolyl cis-trans isomerase B protein	38.46	3	4	5	169	18.6
15965395	signal peptide protein	33.01	3	5	6	209	22.5
15965248;Q92Q55	30S ribosomal protein S2	22.75	3	4	6	255	28.0
15965112;Q92QG7	50S ribosomal protein L2	22.66	3	3	8	278	30.4
15963759;Q92TE7	preprotein translocase subunit SecB	35.12	2	3	9	168	18.3
15965567	ferredoxin, 2FE-2S FDII electron transport iron-sulfur protein	30.19	2	2	2	106	11.4
15965311;P56898	single-stranded DNA-binding protein	26.44	2	3	5	174	19.0
15965105;Q92QH3	30S ribosomal protein S7	25.64	2	2	7	156	17.7
334320817	heat shock protein Hsp20	21.38	2	2	2	159	17.8
15965096	transcription antitermination protein NusG	37.50	1	3	4	176	19.9
15964367	translation initiation factor IF-1	30.56	1	1	1	72	8.3
15965730	dimethylamine corrinoid protein	25.00	1	2	2	232	25.5
15963996;Q92SW1	30S ribosomal protein S15	24.72	1	1	4	89	10.1
15966770	ABC transporter ATP-binding protein	24.72	1	4	4	356	39.7
8571395	NifU-like protein	23.81	1	1	1	63	6.7
334319033	electron transfer flavoprotein subunit alpha	23.46	1	7	8	567	59.4
384533786	hypothetical protein	22.61	1	2	2	261	29.3
8133104	NADP-dependent isocitrate dehydrogenase	21.78	1	4	4	404	45.2
15963984;Q92SX1	hypothetical protein SMc02906	21.50	1	1	2	107	11.6
