# Supplementary material for: Seven-transmembrane receptor protein RgsP and cell wall-binding protein RgsM promote unipolar growth in Rhizobiales
Source: PLoS Genet. 2018 Aug 13;14(8):e1007594. doi: 10.1371/journal.pgen.1007594 (PMC6107284; doi:10.1371/journal.pgen.1007594)
Supplement: S2 Table — Proteins were identified by mass spectrometry. Identified proteins for Rm2011 harboring the empty vector pWBT were removed from the obtained list of candidate interaction partners. MW, molecular weight; AAs, number of amino acids; PSMs, peptide-spectrum matches. (PDF) [file pgen.1007594.s020.pdf]

S2 Table. Co-immunoprecipitation revealed putative interaction partners of RgsM (SMc02432).

Accession	Description	Coverage	Unique Peptides	Peptides	PSMs	AAs	MW [kDa]
NP_386713.1	hypothetical protein SMc02432 (RgsM)	57.28	23	23	62	646	69.7
NP_385024.1	transmembrane signal peptide protein SMc00074 (RgsP)	17.11	11	11	11	970	107.2
NP_386833.1	hypothetical protein SMc00644	31.08	11	11	11	753	80.0
NP_385532.1	hypothetical protein SMc01011	15.41	4	4	4	331	34.8
NP_385748.1	signal peptide protein SMc00950	11.48	2	2	2	209	22.5
NP_385925.1	ubiquinol-cytochrome C reductase iron-sulfur subunit protein	7.81	1	1	1	192	20.5
YP_002122333.1	molecular chaperone GroES (plasmid)	15.38	1	1	1	104	11.4
NP_385964.1	hypothetical protein SMc00150	0.95	1	1	1	631	69.5
NP_387388.1	hypothetical protein SMc03899	30.12	1	1	1	83	9.8
NP_435918.1	NapC membrane-bound tetraheme cytochrome c subunit (plasmid)	10.73	1	1	1	233	26.1
NP_384173.1	ATP-dependent helicase	4.76	1	1	1	820	88.5
NP_435823.1	hypothetical protein SMa1065 (plasmid)	8.02	1	1	1	262	27.9
NP_437769.1	hypothetical protein SM_b21402 (plasmid)	11.63	1	1	2	387	39.1
NP_386896.1	hypothetical protein SMc04006	7.59	1	1	1	145	16.0
NP_386684.1	hypothetical protein SMc02351	18.06	1	1	1	155	17.1
NP_385170.1	DNA-directed RNA polymerase subunit omega	29.63	1	1	1	135	14.9
NP_386284.1	UDP-N-acetylmuramoyl-L-alanyl-D-glutamate synthetase	6.91	1	1	2	463	48.0
