# Supplementary material for: Seven-transmembrane receptor protein RgsP and cell wall-binding protein RgsM promote unipolar growth in Rhizobiales
Source: PLoS Genet. 2018 Aug 13;14(8):e1007594. doi: 10.1371/journal.pgen.1007594 (PMC6107284; doi:10.1371/journal.pgen.1007594)
Supplement: S4 Table — (PDF) [file pgen.1007594.s022.pdf]

S4 Table. Names and mass spectrometry data for the muropeptide fractions shown in S15A Fig.

Peak No.	Muropeptide[1]	Determined neutral mass (amu)	Theoretical mass (amu)
1	Tri	870.36	870.37
2	TetraGly4	927.40	927.39
3	Tetra	941.41	941.41
4	Penta	1012.45	1012.44
5	TetraTriDapGly4	1779.76	1779.75
6	TriTriDap	1722.75	1722.73
7	TetraTriDap	1793.79	1779.75
8	TetraTri	1793.78	1779.75
9	TetraTetra	1864.82	1864.80
10	TetraAnh	921.39	921.38
11	TetraPenta	1935.86	1935.84
12	TetraTetraTriDap	2717.18	2717.16
13	PentaAnh	992.42	992.42
14	TetraTetraTri	2717.18	2717.16
15	TriTriDapAnh +	1702.71	1702.70
	TetraTetraTetra	2788.21	2788.20
16	TetraTriDapAnh	1773.75	1773.74
	or TetraTriAnh	1773.75	1773.74
17	TetraTetraAnh	1844.79	1844.78

[1]Muropeptides are named according to ref. 81.
