# Supplementary material for: Seven-transmembrane receptor protein RgsP and cell wall-binding protein RgsM promote unipolar growth in Rhizobiales
Source: PLoS Genet. 2018 Aug 13;14(8):e1007594. doi: 10.1371/journal.pgen.1007594 (PMC6107284; doi:10.1371/journal.pgen.1007594)
Supplement: S5 Table — (PDF) [file pgen.1007594.s023.pdf]

S5 Table. Strains and plasmids used in this study.

Strain	Properties	Reference
S. meliloti		
Rm2011	Wild type, Str^r	[1]
Rm2011 ΔXVI	Rm2011 with 16 deletions of GGDEF domain-encoding genes, c-di-GMP^0	[2]
Rm2011 $P_{lac\text{-}T5}$-*rgsP*	Rm2011 *rgsP*::pK18mob2-$P_{lac\text{-}T5}$-*rgsP*, conditional RgsP depletion upon *lacI* expression (yields strain Rm2011 *rgsP*^{dpl}), Km^r	This work
Rm2011 ΔXVI $P_{lac\text{-}T5}$-*rgsP*	Rm2011 ΔXVI *rgsP*::pK18mob2-$P_{lac\text{-}T5}$-*rgsP*, conditional RgsP depletion upon *lacI* expression (yields strain Rm2011 ΔXVI *rgsP*^{dpl}), Km^r	This work
Rm2011 P_{lac}-*rgsM*	Rm2011 *rgsM*::pK18mob2-P_{lac}-*rgsM*, conditional RgsM depletion upon *lacI* expression (yields strain Rm2011 *rgsM*^{dpl}), Km^r	This work
Rm2011 *rgsP-egfp*	Rm2011 expressing *egfp*-tagged *rgsP*, markerless insertion	This work
Rm2011 *rgsP-mCherry*	Rm2011 expressing *mCherry*-tagged *rgsP*, markerless insertion	This work
Rm2011 *rgsP-mCherry pleD-egfp*	Rm2011 *rgsP-mCherry pleD*::pK18mob2-*pleD-egfp*, Km^r	This work
Rm2011 *rgsP-egfp*$_{Km}$	Rm2011 *rgsP*::pK18mob2-*rgsP-egfp*, Km^r	This work
Rm2011 ΔXVI *rgsP-egfp*$_{Km}$	Rm2011 ΔXVI *rgsP*::pK18mob2-*rgsP-egfp*, Km^r	This work
Rm2011 *rgsP-3×flag*	Rm2011 *rgsP*::pG18mob-*rgsP-3×flag*, Gm^r	This work
Rm2011 *rgsM-3×flag*	Rm2011 *rgsM*::pG18mob-*rgsM-3×flag*, Gm^r	This work
Rm2011 *3×flag-mucR*	Rm2011 expressing *3×flag*-tagged *mucR*, markerless insertion	This work
Rm2011 *3×flag-mucR* $P_{lac\text{-}T5}$-*rgsP*	Rm2011 *3×flag-mucR rgsP*::pK18mob2-$P_{lac\text{-}T5}$-*rgsP*, conditional RgsP depletion upon *lacI* expression (yields strain Rm2011 *3×flag-mucR rgsP*^{dpl}), Km^r	This work
Rm2011 *3×flag-mucR rgsP-3×flag*	Rm2011 *3×flag-mucR* expressing *3×flag*-tagged *rgsP*, markerless insertion	This work
Rm2011 *3×flag-mucR rgsM-3×flag*	Rm2011 *3×flag-mucR* expressing *3×flag*-tagged *rgsM*, markerless insertion	This work
Rm2011 *3×flag-mucR* $P_{lac\text{-}T5}$-*rgsP-3×flag*	Rm2011 *3×flag-mucR rgsP-3×flag*::pK18mob2-$P_{lac\text{-}T5}$-*rgsP,* conditional RgsP-3×FLAG depletion upon *lacI* expression (yields strain Rm2011 *3×flag-mucR rgsP-3×flag*^{dpl}), Km^r	This work
Rm2011 *3×flag-mucR* P_{lac}-*rgsM-3×flag*	Rm2011 *3×flag-mucR rgsM-3×flag*::pK18mob2-P_{lac}-*rgsM,* conditional RgsM-3×FLAG depletion upon *lacI* expression (yields strain Rm2011 *3×flag-mucR rgsM-3×flag*^{dpl}), Km^r	This work
Rm2011 *mVenus-rgsM*	Rm2011 expressing *mVenus*-tagged *rgsM*, markerless insertion	This work
Rm2011 *mVenus-rgsM rgsP-mCherry*	Rm2011 *mVenus-rgsM* expressing *mCherry*-tagged *rgsP*, markerless insertion	This work
Rm2011 *mVenus-rgsM* $P_{lac\text{-}T5}$-*rgsP*	Rm2011 *mVenus-rgsM rgsP*::pK18mob2-$P_{lac\text{-}T5}$-*rgsP,* conditional RgsP depletion upon *lacI* expression (yields strain Rm2011 *mVenus-rgsM rgsP*^{dpl}), Km^r	This work
Rm2011 *rgsP-mCherry* P_{lac}-*rgsM*	Rm2011 *rgsP-mCherry rgsM*::pK18mob2-P_{lac}-*rgsM,* conditional RgsM depletion upon *lacI* expression (yields strain Rm2011 *rgsP-mCherry rgsM*^{dpl}), Km^r	This work
A. tumefaciens		
C58	Wild type, isolated from a cherry tree (*Prunus*) tumor	[3]
C58 *Atu0784-egfp*	C58 *Atu0784*::pK18mob2-*rgsP*$_{At}$-*egfp*, Km^r	This work
R. etli		
CFN42	*R. etli* type strain, isolated from *Phaseolus*, Sm^r	[4]
CFN42 *RHE_CH00976-egfp*	CFN42 *RHE_CH00976*::pK18mob2-*rgsP*$_{Re}$-*egfp*, Km^r	This work
E. coli		
BL21(DE3)	F$^-$ *ompT gal dcm lon hsdS*$_B$($r_B^-m_B^-$) λ(DE3 [*lacI lacUV5-T7p07 ind1 sam7 nin5*]) [*malB*$^+$]$_{K\text{-}12}$(λ^S)	New England Biolabs
DH5α	F$^-$ *endA1 glnV44 thi-1 recA1 relA1 gyrA96 deoR nupG purB20* φ80d*lacZ*ΔM15 Δ(*lacZYA-argF*)U169, hsdR17($r_K^-m_K^+$), λ$^-$	[5]
S17-1	*E. coli* 294 Thi RP4-2-Tc::Mu-Km::Tn7 integrated into the chromosome	[6]
BTH101	*cya,* BACTH reporter strain	[7]
**Plasmids**		
pABC2S-mob	variant of mobilizable *repABC*-based mini-replicon, single-copy in *S. meliloti*, Spec^r	[8]
pG18mob	suicide vector, *lacZ*, *mob*, Gm^r	[9]
pK18mob2	suicide vector, *lacZ*, *mob*, Km^r	[9]
pK18mob2-*egfp*	pK18mob2 carrying *egfp, lacZ*, *mob,* Km^r	N. Meier
pK18mobsacB	suicide vector, *lacZ*, *mob*, *sacB,* Km^r	[9]
pK19mob2ΩHMB	integrative plasmid carrying a transcription-termination sequence, Km^r	[10]
pR_*egfp*	reporter fusion plasmid carrying P_{syn} for constitutive expression, Tc^r	[11]
pR-P_{tau}	pR_*egfp* carrying the *tauA* promoter from *S. meliloti* in place of P_{syn}, Tc^r	M. Robledo
pSRKGm	pBBR1MCS-5-derived broad-host-range expression vector containing *lac* promoter and *lacI*^{q}, *lacZ*α$^+$, Gm^r	[12]
pSRKGm-*parB-mCherry*	pSRKGm carrying *S. meliloti parB* fused to *mCherry*, Gm^r	[2]
pSRKKm	pBBR1MCS-5-derived broad-host-range expression vector containing *lac*	[12]

	promoter and *lacI*^{q}, *lacZ*α$^+$, Km^r	
pSRKKm-*egfp*	pSRKKm carrying *egfp*, Km^r	[2]
pWBT	pSRKGm carrying T5 promoter, Gm^r	M. McIntosh
pWH844	expression vector carrying *6×his* and T5 promoter, Amp^r	[13]
pKT25	Plasmid for constructing C-terminal fusions to T25, Km^r	[7]
pUT18	Plasmid for constructing N-terminal fusions to T18, Amp^r	[7]
pUT18C	Plasmid for constructing C-terminal fusions to T18, Amp^r	[7]
pUT18C-*zip*	pUT18C carrying the leucine zipper of GCN4	[7]
pKT25-*zip*	pKT25 carrying the leucine zipper of GCN4	[7]
pG18mob-*3×flag*	pG18mob carrying *3×flag* including a stop codon cloned into the XbaI and HindIII restriction sites, Gm^r	This work
pSRKGm-*mCherry*	pSRKGm carrying *mCherry,* Gm^r	This work
pSRKGm-*mCherry-ftsZ*	pSRKGm carrying *S. meliloti ftsZ* fused to *mCherry*, Gm^r	This work
pSRKKm-P$_{rgsP}$-*egfp*	pSRKKm-*egfp* carrying *rgsP* upstream fragment	This work
pSRKKm-P$_{rimJ}$-P$_{rgsP}$-*egfp*	pSRKKm-*egfp* carrying *rimJ* and *rgsP* upstream fragment	This work
pUT18Spe	pUT18 with a SpeI restriction site upstream of T18 start codon	This work
pUT18Spe-*rgsP*$_{\Delta GGDEF\Delta EAL}$	pUT18Spe carrying *rgsP*(AA$_{1\text{-}524}$)	This work
pUT18C-*rgsM*	pUT18C carrying *rgsM*	This work
pKT25-*rgsM*	pKT25 carrying *rgsM*	This work
<u>Complementation and overexpression constructs</u>		
pDJS31	pET24b(+) carrying *dgcA*^{wt}, Km^r	[14]
pDJS30	pET24b(+) carrying *dmxB*^{wt}, Km^r	[15]
pWBT-*SMc00074*	pWBT carrying *rgsP* (*SMc00074*)	[2]
pWH844-*SMc00074*$_{390\text{-}970}$	pWH844 carrying *rgsP*(AA$_{390\text{-}970}$)	[2]
pABC-*rgsP*	pABC2S-mob carrying 944 bp *rgsP* upstream fragment with a stop codon in *rimJ* at nucleotide position 64, *rgsP* and *egfp*	This work
pABC-*rgsP-egfp*	pABC-*rgsP* without *rgsP* stop codon	This work
pABC-*rgsP-3×flag*	pABC-*rgsP-egfp*	This work
pABC-*rgsP*$_{AAL}$	pABC-*rgsP* containing E746A mutation	This work
pABC-*rgsP*$_{AAL}$-*egfp*	pABC-*rgsP*$_{AAL}$ without *rgsP* stop codon	This work
pABC-*rgsP*$_{AAL}$-*3×flag*	pABC-*rgsP*$_{AAL}$-*egfp* carrying *3×flag* in place of *egfp*	This work
pABC-*rgsP*$_{GAAAF}$	pABC-*rgsP* containing G619A, D620A and Q621A mutations	This work
pABC-*rgsP*$_{GAAAF}$-*egfp*	pABC-*rgsP*$_{GAAAF}$ without *rgsP* stop codon	This work
pABC-*rgsP*$_{GAAAF}$-*3×flag*	pABC-*rgsP*$_{GAAAF}$-*egfp* carrying *3×flag* in place of *egfp*	This work
pABC-*rgsP*$_{AxxA}$	pABC-*rgsP* R609A and D612A mutations	This work
pABC-*rgsP*$_{AxxA}$-*egfp*	pABC-*rgsP*$_{AxxA}$ without *rgsP* stop codon	This work
pABC-*rgsP*$_{AxxA}$-*3×flag*	pABC-*rgsP*$_{AxxA}$-*egfp* carrying *3×flag* in place of *egfp*	This work
pABC-*rgsP*$_{\Delta EAL}$	pABC-*rgsP* with deletion of *rgsP*(AA$_{716\text{-}970}$)	This work
pABC-*rgsP*$_{\Delta EAL}$-*egfp*	pABC-*rgsP*$_{\Delta EAL}$ without *rgsP* stop codon	This work
pABC-*rgsP*$_{\Delta EAL}$-*3×flag*	pABC-*rgsP*$_{\Delta EAL}$-*egfp* carrying *3×flag* in place of *egfp*	This work
pABC-*rgsP*$_{\Delta GGDEF}$	pABC-*rgsP* with deletion of *rgsP*(AA$_{541\text{-}697}$)	This work
pABC-*rgsP*$_{\Delta GGDEF}$-*egfp*	pABC-*rgsP*$_{\Delta GGDEF}$ without *rgsP* stop codon	This work
pABC-*rgsP*$_{\Delta GGDEF}$-*3×flag*	pABC-*rgsP*$_{\Delta GGDEF}$-*egfp* carrying *3×flag* in place of *egfp*	This work
pABC-*rgsP*$_{\Delta GGDEF\Delta EAL}$	pABC-*rgsP* with deletion of *rgsP*(AA$_{541\text{-}970}$)	This work
pABC-*rgsP*$_{\Delta GGDEF\Delta EAL}$-*egfp*	pABC-*rgsP*$_{\Delta GGDEF\Delta EAL}$ without *rgsP* stop codon	This work
pABC-*rgsP*$_{\Delta GGDEF\Delta EAL}$-*3×flag*	pABC-*rgsP*$_{\Delta GGDEF\Delta EAL}$-*egfp* carrying *3×flag* in place of *egfp*	This work
pABC-*rgsP*$_{\Delta PAS}$	pABC-*rgsP* with deletion of *rgsP*(AA$_{423\text{-}524}$)	This work
pABC-*rgsP*$_{\Delta PAS}$-*egfp*	pABC-*rgsP*$_{\Delta PAS}$ without *rgsP* stop codon	This work
pABC-*rgsP*$_{\Delta PAS}$-*3×flag*	pABC-*rgsP*$_{\Delta PAS}$-*egfp* carrying *3×flag* in place of *egfp*	This work
pABC-*rgsP*$_{\Delta PAS\Delta GGDEF\Delta EAL}$	pABC-*rgsP* with deletion of *rgsP*(AA$_{417\text{-}970}$)	This work
pABC-*rgsP*$_{\Delta PAS\Delta GGDEF\Delta EAL}$-*egfp*	pABC-*rgsP*$_{\Delta PAS\Delta GGDEF\Delta EAL}$ without *rgsP* stop codon	This work
pABC-*rgsP*$_{\Delta PAS\Delta GGDEF\Delta EAL}$-*3×flag*	pABC-*rgsP*$_{\Delta PAS\Delta GGDEF\Delta EAL}$-*egfp* carrying *3×flag* in place of *egfp*	This work
pABC-*rgsP*$_{\Delta \text{7TMR-DISMED2}}$	pABC-*rgsP* with deletion of *rgsP*(AA$_{49\text{-}183}$)	This work
pABC-*rgsP*$_{\Delta \text{7TMR-DISMED2}}$-*egfp*	pABC-*rgsP*$_{\Delta \text{7TMR-DISMED2}}$ without *rgsP* stop codon	This work
pABC-*rgsP*$_{\Delta \text{7TMR-DISMED2}}$-*3×flag*	pABC-*rgsP*$_{\Delta \text{7TMR-DISMED2}}$-*egfp* carrying *3×flag* in place of *egfp*	This work
pABC-*rgsP*$_{\Delta \text{7TMR-DISMED2}\Delta \text{7TMR-DISM\_7TM}}$	pABC-*rgsP* with deletion of *rgsP*(AA$_{49\text{-}363}$)	This work
pABC-*rgsP*$_{\Delta \text{7TMR-DISMED2}\Delta \text{7TMR-DISM\_7TM}}$-*egfp*	pABC-*rgsP*$_{\Delta \text{7TMR-DISMED2}\Delta \text{7TMR-DISM\_7TM}}$ without *rgsP* stop codon	This work
pABC-*rgsP*$_{\Delta \text{7TMR-DISMED2}\Delta \text{7TMR-DISM\_7TM}}$-*3×flag*	pABC-*rgsP*$_{\Delta \text{7TMR-DISMED2}\Delta \text{7TMR-DISM\_7TM}}$-*egfp* carrying *3×flag* in place of *egfp*	This work
pR-*rgsP*	pR_*egfp* carrying *rgsP* and 114 bp *rgsP* upstream region	This work
pR-*rgsP-3×flag*	pR-*rgsP* without *rgsP* stop codon and carrying *3×flag*	This work
pR-*rgsP*$_{AAL}$	pR-*rgsP* containing E746A mutation	This work
pR-*rgsP*$_{GAAAF}$	pR-*rgsP* containing G619A, D620A and Q621A mutations	This work
pR-*rgsP*$_{AxxA}$	pR-*rgsP* containing R609A and D612A mutations	This work
pR-*rgsP*$_{\Delta EAL}$	pR-*rgsP* with deletion of *rgsP*(AA$_{716\text{-}970}$)	This work
pR-*rgsP*$_{\Delta EAL}$-*3×flag*	pR-*rgsP*$_{\Delta EAL}$ without *rgsP* stop codon and carrying *3×flag*	This work
pR-*rgsP*$_{\Delta GGDEF}$	pR-*rgsP* with deletion of *rgsP*(AA$_{541\text{-}697}$)	This work
pR-*rgsP*$_{\Delta GGDEF}$-*3×flag*	pR-*rgsP*$_{\Delta GGDEF}$ without *rgsP* stop codon and carrying *3×flag*	This work
pR-*rgsP*$_{\Delta GGDEF\Delta EAL}$	pR-*rgsP* with deletion of *rgsP*(AA$_{541\text{-}970}$)	This work
pR-*rgsP*$_{\Delta GGDEF\Delta EAL}$-*3×flag*	pR-*rgsP*$_{\Delta GGDEF\Delta EAL}$ without *rgsP* stop codon and carrying *3×flag*	This work

pR-$rgsP_{\Delta PAS}$	pR-*rgsP* with deletion of *rgsP*($AA_{423-524}$)	This work
pR-$rgsP_{\Delta PAS}$-*3×flag*	pR-$rgsP_{\Delta PAS}$ without *rgsP* stop codon and carrying *3×flag*	This work
pR-$rgsP_{\Delta PAS\Delta GGDEF\Delta EAL}$	pR-*rgsP* with deletion of *rgsP*($AA_{423-970}$)	This work
pR-$rgsP_{\Delta PAS\Delta GGDEF\Delta EAL}$-*3×flag*	pR-$rgsP_{\Delta PAS\Delta GGDEF\Delta EAL}$ without *rgsP* stop codon and carrying *3×flag*	This work
pR-$rgsP_{\Delta 7TMR\text{-}DISMED2}$	pR-*rgsP* with deletion of *rgsP*(AA_{49-183})	This work
pR-$rgsP_{\Delta 7TMR\text{-}DISMED2}$-*3×flag*	pR-$rgsP_{\Delta 7TMR\text{-}DISMED2}$ without *rgsP* stop codon and carrying *3×flag*	This work
pR-$rgsP_{\Delta 7TMR\text{-}DISMED2\Delta 7TMR\text{-}DISM\_7TM}$	pR-*rgsP* with deletion of *rgsP*(AA_{49-363})	This work
pR-$rgsP_{\Delta 7TMR\text{-}DISMED2\Delta 7TMR\text{-}DISM\_7TM}$-*3×flag*	pR-$rgsP_{\Delta 7TMR\text{-}DISMED2\Delta 7TMR\text{-}DISM\_7TM}$ without *rgsP* stop codon and carrying *3×flag*	This work
pR-P_{tau}-$rgsP_{At}$	pR-P_{tau} carrying *Atu0784*	This work
pR-P_{tau}-$rgsP_{Re}$	pR-P_{tau} carrying *RHE_CH00976*	This work
pR-P_{tau}-$rgsP_{Sm}$	pR-P_{tau} carrying *SMc00074*	This work
pR-P_{tau}-$rgsM_{At}$	pR-P_{tau} carrying *Atu4178*	This work
pR-P_{tau}-$rgsM_{Re}$	pR-P_{tau} carrying *RHE_CH03752*	This work
pR-P_{tau}-$rgsM_{Sm}$	pR-P_{tau} carrying *SMc02432*	This work
pSRKKm-*rgsM-phoA*	pSRKKm carrying *rgsM* fused to $phoA_{Ec}$(AA_{27-471})	This work
pSRKKm-$rgsM_{1-66}$-*phoA*	pSRKKm carrying *rgsM*(AA_{1-66}) fused to $phoA_{Ec}$(AA_{27-471})	This work
pSRKKm-$rgsM_{60-646}$-*phoA*	pSRKKm carrying *rgsM*(AA_{60-646}) fused to $phoA_{Ec}$(AA_{27-471})	This work
pWBT-*rgsM*	pWBT carrying *rgsM*	This work
pWBT-*rgsM-3×flag*	pWBT-*rgsM* without *rgsM* stop codon and carrying *3×flag*	This work
pWBT-$rgsM_{H510A}$	pWBT-*rgsM* containing H510A mutation	This work
pWBT-$rgsM_{H510A}$-*3×flag*	pWBT-$rgsM_{H510A}$ without *rgsM* stop codon and carrying *3×flag*	This work
pWH844-$rgsP_{7TMR\text{-}DISMED2}$	pWH844 carrying *rgsP*(AA_{38-194})	This work
pWH844-$rgsP_{PAS\text{-}GGDEF}$	pWH844 carrying *rgsP*($AA_{390-715}$)	This work
pWH844-$rgsP_{PAS\text{-}GGDEF\text{-}EAL}$-AAL	pWH844-$SMc00074_{390-970}$ containing E746A mutation	This work
pWH844-$rgsM_{57-646}$	pWH844 carrying *rgsM*(AA_{57-646})	This work
pWH844-$rgsM_{260-646}$	pWH844 carrying *rgsM*($AA_{260-646}$)	This work
<u>Integrative plasmids</u>		
pK18mob2-*pleD-egfp*	pK18mob2-*egfp* carrying 3' portion of *pleD*	[2]
pG18mob-*rgsP-3×flag*	pG18mob-*3×flag* carrying 3' portion of *rgsP*	This work
pG18mob-*rgsM-3×flag*	pG18mob-*3×flag* carrying 3' portion of *rgsM*	This work
pK18mob2-*rgsP-egfp*	pK18mob2-*egfp* carrying 3' portion of *rgsP*	This work
pK18mob2-$rgsP_{At}$-*egfp*	pK18mob2-*egfp* carrying 3' portion of *Atu0784*	This work
pK18mob2-$rgsP_{Re}$-*egfp*	pK18mob2-*egfp* carrying 3' portion of *RHE_CH00976*	This work
pK18mob2-P_{lac}-*rgsM*	pK18mob2 carrying 5' portion of *rgsM*	This work
pK18mob2-$P_{lac\text{-}T5}$-*rgsP*	pK18mob2 carrying T5 promoter, a Shine-Dalgarno sequence and 5' portion of *rgsP*	This work
pK18mobsacB-*rgsP-3×flag*	pK18mobsacB carrying 3' portion of *rgsP* excluding the stop codon, *3×flag* and *rgsP* downstream fragment	This work
pK18mobsacB-*rgsM-3×flag*	pK18mobsacB carrying 3' portion of *rgsM* excluding the stop codon, *mCherry* and *rgsM* downstream fragment	This work
pK18mobsacB-*rgsP-egfp*	pK18mobsacB carrying 3' portion of *rgsP* excluding the stop codon, *egfp* and *rgsP* downstream fragment	This work
pK18mobsacB-*rgsP-mCherry*	pK18mobsacB carrying 3' portion of *rgsP* excluding the stop codon, *mCherry* and *rgsP* downstream fragment	This work
pK18mobsacB-*mVenus-rgsM*	pK18mobsacB carrying 5' portion of *rgsM* excluding the start codon, *mVenus* and *rgsM* upstream fragment	This work
pK18mobsacB-*3×flag-mucR*	pK18mobsacB carrying *mucR* excluding the start codon, *3×flag* and *mucR* upstream fragment	This work
pK19mob2ΩHMB-*rgsM*	pK19mob2ΩHMB carrying internal portion of *rgsM*	This work

1. Casse F, Boucher C, Julliot J, Michel M, Dénarié J (1979) Identification and characterization of large plasmids in *Rhizobium meliloti* using agarose gel electrophoresis. *Microbiology* 113:229-242.
2. Schäper S, Krol E, Skotnicka D, Kaever V, Hilker R, Søgaard-Andersen L, et al. (2016) Cyclic di-GMP regulates multiple cellular functions in the symbiotic Alphaproteobacterium *Sinorhizobium meliloti*. J Bacteriol 198:521-535. doi: 10.1128/JB.00795-15. PMID: 26574513
3. Hamilton RH, Fall MZ (1971) The loss of tumor-initiating ability in *Agrobacterium tumefaciens* by incubation at high temperature. Experientia 27:229-230. PMID: 5544763
4. Quinto C, De La Vega H, Flores M, Leemans J, Cevallos MA, Pardo MA, et al. (1985) Nitrogenase reductase: A functional multigene family in *Rhizobium phaseoli*. Proc Natl Acad Sci U S A 82:1170-1174. PMID: 16593543
5. Grant SG, Jessee J, Bloom FR, Hanahan D (1990) Differential plasmid rescue from transgenic mouse DNAs into *Escherichia coli* methylation-restriction mutants. Proc Natl Acad Sci U S A 87:4645-4649. PMID: 2162051
6. Simon R, Priefer U, Pühler A (1983) A broad host range mobilization system for in vivo genetic engineering: transposon mutagenesis in gram-negative bacteria. Nature Biotechnology 1:784-791
7. Karimova G, Pidoux J, Ullmann A, Ladant D (1998) A bacterial two-hybrid system based on a reconstituted

signal transduction pathway. Proc Natl Acad Sci U S A 95:5752-5756. PubMed PMID: 9576956

8. Döhlemann J, Wagner M, Happel C, Carillo M, Sobetzko P, Erb TJ, et al. (2017) A family of single copy *repABC*-type shuttle vectors stably maintained in the alpha-proteobacterium *Sinorhizobium meliloti*. ACS Synth Biol. Epub 2017/03/06. doi: 10.1021/acssynbio.6b00320. PMID: 28264559
9. Schäfer A, Tauch A, Jäger W, Kalinowski J, Thierbach G, Pühler A (1994) Small mobilizable multi-purpose cloning vectors derived from the *Escherichia coli* plasmids pK18 and pK19: selection of defined deletions in the chromosome of *Corynebacterium glutamicum*. Gene 145:69-73. PMID: 8045426.
10. Luo L, Yao SY, Becker A, Rüberg S, Yu GQ, Zhu JB, et al. (2005) Two new *Sinorhizobium meliloti* LysR-type transcriptional regulators required for nodulation. J Bacteriol 187:4562-4572. doi: 10.1128/JB.187.13.4562-4572.2005. PMID: 15968067
11. Torres-Quesada O, Millán V, Nisa-Martínez R, Bardou F, Crespi M, Toro N, et al. (2013) Independent activity of the homologous small regulatory RNAs AbcR1 and AbcR2 in the legume symbiont *Sinorhizobium meliloti*. PLoS One 8:e68147. doi: 10.1371/journal.pone.0068147. PMID: 23869210
12. Khan SR, Gaines J, Roop RM, Farrand SK (2008) Broad-host-range expression vectors with tightly regulated promoters and their use to examine the influence of TraR and TraM expression on Ti plasmid quorum sensing. Appl Environ Microbiol 74:5053-5062. doi: 10.1128/AEM.01098-08. PMID: 18606801
13. Schirmer F, Ehrt S, Hillen W (1997) Expression, inducer spectrum, domain structure, and function of MopR, the regulator of phenol degradation in *Acinetobacter calcoaceticus* NCIB8250. J Bacteriol 179:1329-1336. PMID: 9023219
14. Skotnicka D, Smaldone GT, Petters T, Trampari E, Liang J, Kaever V, et al. (2016) A minimal threshold of c-di-GMP is essential for fruiting body formation and sporulation in *Myxococcus xanthus*. PLoS Genet 12:e1006080. doi: 10.1371/journal.pgen.1006080. PMID: 27214040
15. Skotnicka D, Petters T, Heering J, Hoppert M, Kaever V, Søgaard-Andersen L (2016) Cyclic di-GMP regulates type IV pilus-dependent motility in *Myxococcus xanthus*. J Bacteriol 198:77-90. doi: 10.1128/JB.00281-15. PMID: 26124238
