# Supplementary material for: Seven-transmembrane receptor protein RgsP and cell wall-binding protein RgsM promote unipolar growth in Rhizobiales
Source: PLoS Genet. 2018 Aug 13;14(8):e1007594. doi: 10.1371/journal.pgen.1007594 (PMC6107284; doi:10.1371/journal.pgen.1007594)
Supplement: S6 Table — (PDF) [file pgen.1007594.s024.pdf]

S6 Table. Oligonucleotides used in this study.

**Primer**	**Sequence**	**Purpose**
PrimJ_NotI_fwd	ATATGCGGCCGCTGAACGACATATTGTCCTCGCTGA	Construction of pABC-*rgsP* and its derivatives
SMc00074_1243_Van91I_rev	ATATCCAGCGCCTGGCGCTCGAGAT	
RimJ_51_stop_fwd	TGACATTATCTGCTCCGCCTCCC	
RimJ_51_stop_rev2	GGGAGGCGGAGCAGATAATGTCACTGGCTGGCGATCTCGAC	
EGFP-Van91I-KpnI_fwd	ATATCCAGGCGCTGGCGCTGGGTACCAGCAAGGGCGAGGAGCTGTT	
EGFP-ScaI_rev	ATATAGTACTTTACTTGTACAGCTCGTCCATGC	
EGFP-Van91I-Stop-KpnI_fwd	ATATCCAGGCGCTGGCGCTGTGAGGTACCAGCAAGGGCGAGGAGCTGTT	
SMc00074_1243_Van91I_fwd	ATATCCAGGCGCTGGCGCTCAC	
SMc00074+stop_KpnI_rev	ATATGGTACCTCAAGCCCGCTTCATCAGCGGAAAT	
SMc00074-nostop_KpnI_rev	ATATGGTACCAGCCCGCTTCATCAGCGGAAAT	
SMc00074GGDEF+stop_KpnI_rev	ATATGGTACCTCATTCGAGCTGTAGGCGGTCC	
SMc00074GGDEF-nostop_KpnI_rev	ATATGGTACCTTCGAGCTGTAGGCGGTCC	
SMc00074PAS+stop_KpnI_rev	ATATGGTACCTCACAGAAGCGCGTTGTGCAA	
SMc00074PAS-nostop_KpnI_rev	ATATGGTACCCAGAAGCGCGTTGTGCAA	
SMc00074-full_-100_Bam_f	ATATGGATCCTGAGCGTGAAGGCTATTTGAGA	Construction of pR-*rgsP* and its derivatives
SMc00074-full-Xba_r	ATATTCTAGATCAAGCCCGCTTCATCAG	
SMc00074_EAL-del_Xba_r	ATATTCTAGATCATTCGAGCTGTAGGCGGT	
SMc00074_GGDEF-EAL-del_Xba_r	ATATTCTAGATCACAGAAGCGCGTTGTG	
SMc00074_PAS-GGDEF-EAL-del_Xba_r	ATATTCTAGATCAGGTATCGCCTGATCCC	
SMc00074_E>A-f	GCGGCCCTCATGCGCTGGGA	Generating $rgsP_{AAL}$
SMc00074_E>A-r	AGCGCATGAGGGCCGCGAAGCCGGCGATCTCGGCAT	
SMc00074_GGDQF>GAAAF_f	GCCGCGGCCTTCGGCTTGATCCTCATGT	Generating $rgsP_{GAAAF}$
SMc00074_GGDQF>GAAAF_r	AGGATCAAGCCGAAGGCCGCGGCGCCAAGACGCGCGAGCGT	
SMc00074_Isite>AxxA_f	GCCCCGCAGGCCACGCTCGCGCGTCTTGGC	Generating $rgsP_{AxxA}$
SMc00074_Isite>AxxA_r	ACGCGCGAGCGTGGCCTGCGGGGCCAGAAGCCTGCGCAGGCG	
SMc00074_GGDEF-del_f	GAGCCGTTCCGTCCGGCTTT	Generating $rgsP_{\Delta GGDEF}$
SMc00074_GGDEF-del_r	GACGGAACGGCTCCAGAAGCGCGTTGTGCAA	
SMc00074_PAS-del_f	ACCGAACAGCGCAATTCG	Generating $rgsP_{\Delta PAS}$
SMc00074_PAS-del_r	TGCGCTGTTCGGTGGTATCGCCTGATCCCGTGA	
SMc00074_DISMED2-del_f	GACGCCTACAAGGATACCGT	Generating $rgsP_{\Delta 7TMR\text{-}DISMED2}$
SMc00074_DISMED2-del_r	TCCTTGTAGGCGTCCGCCGTGTCCTCGCGCGA	
SMc00074_DISM-6TM-del_f	ACCGGTCAACTCGCCAAT	Generating $rgsP_{\Delta 7TMR\text{-}DISMED2\Delta 7TMR\text{-}DISM\_7TM}$
SMc00074_DISM-6TM-del_r	CGAGTTGACCGGTCGCCGTGTCCTCGCGCGA	
SMc02432_H510A_fwd	GCCACCGGCGTCGACTGGTCC	Generating $rgsM_{H510A}$
SMc02432_H510A_rev	GACCAGTCGACGCCGGTGGCCATGCGCGAGAAGCCGAGGATA	
SMc00074-390_Bam_fwd	ATATGGATCCACCGTCATGCAGCACGCCTTTT	Construction of pWH844-$RgsP_{PAS\text{-}GGDEF\text{-}EAL}$-AAL and pWH844-$RgsP_{PAS\text{-}GGDEF}$
SMc00074_Sal_rev	ATATGTCGACTCAAGCCCGCTTCATCAG	
SMc00074-715_Sal_rev	ATATGTCGACTCATTCGAGCTGTAGGCGGTCC	
SMc00074-38_Bam_fwd	ATATGGATCCGAGCCGGTGAAGATCTCG	Construction of pWH844-$rgsP_{7TMR\text{-}DISMED2}$
SMc00074-194_+stop_SalI_rev	ATATGTCGACTCAGGTGAAGGCGTTGACGGTAT	

SMc02432_57_Xho_fwd<br>SMc02432_Pst_rev	ATATCTCGAGCTCGACGGCCGGCAGCAACT<br>ATATCTGCAGCTACTTGCTTGCGACCTCGTT	Construction of pWH844-*rgsM*$_{57\text{-}646}$
SMc02432+778-BglII-f<br>SMc02432-stop-Pst	ATATAGATCTAAGCAGAGCATCGAATACGCC<br>TTGCCTGCAGCTACTTGCTTGCGACCTCGTT	Construction of pWH844-*rgsM*$_{260\text{-}646}$
SMc02432-full-Xba-f<br>SMc02432-full-Kpn-r	ATATTCTAGAATGAGCAGCGATAAGAACATGC<br>ATATGGTACCCTACTTGCTTGCGACCTCGTT	Construction of pWBT-*rgsM* and pWBT-*rgsM*$_{\text{H510A}}$
SMc00074-TIR-RBS_Bam_f<br>SMc00074-full-Xba_r	ATATGGATCCTGATTAACTTTATAAGGAGGAAAAACATATGCCCCTGACCCGTAAGC<br>ATATTCTAGATCAAGCCCGCTTCATCAG	Construction of pR-P$_{tau}$-*rgsP*$_{\text{Sm}}$
RHE_CH00976-TIR-RBS_Bam_f<br>RHE_CH00976-full_Xba_r	ATATGGATCCTGATTAACTTTATAAGGAGGAAAAACATATGCTGCCCAAGTCACCGT<br>ATATTCTAGATCAGGCCCTTTTCGTCAGCG	Construction of pR-P$_{tau}$-*rgsP*$_{\text{Re}}$
Atu0784-TIR-RBS_Bam_f<br>Atu0784-full_Xba_r	ATATGGATCCTGATTAACTTTATAAGGAGGAAAAACATATGACCTACAATCACACTGCGCC<br>ATATTCTAGATCAAGACGCCCGCTTCGT	Construction of pR-P$_{tau}$-*rgsP*$_{\text{At}}$
SMc02432-full-Xba-f<br>SMc02432-full-X-r	ATATTCTAGAATGAGCAGCGATAAGAACATGC<br>ATATTCTAGACTACTTGCTTGCGACCTCGTT	Construction of pR-P$_{tau}$-*rgsM*$_{\text{Sm}}$
RHE_CH03752-TIR-RBS_Xba_f<br>RHE_CH03752-full_Xba_r	ATATTCTAGATGATTAACTTTATAAGGAGGAAAAACATATGATCCGCTCGCTGGGCAA<br>ATATTCTAGATTATTTGCTCGCCACCTGATCG	Construction of pR-P$_{tau}$-*rgsM*$_{\text{Re}}$
Atu4178-TIR-RBS_Bam_f<br>Atu4178-full_Xba_r	ATATGGATCCTGATTAACTTTATAAGGAGGAAAAACATATGACTGCGGATCGCAATGT<br>ATATTCTAGATCAGCGGCTCGCCACCTGAT	Construction of pR-P$_{tau}$-*rgsM*$_{\text{At}}$
Psmc00074_Hind_fwd<br>Psmc00074_Xba_rev<br>PrimJ_Hind_fwd	ATATAAGCTTCGGCGCTGCGCAGAACTG<br>ATATTCTAGACAGGGGCATCAAAGGGGCAAAT<br>ATATAAGCTTTGAACGACATATTGTCCTCGCTGA	Construction of pSRKKm-P$_{rgsP}$-*egfp* and pSRKKm-P$_{rimJ}$-P$_{rgsP}$-*egfp*
SMc00074-C-SalI-fwd<br>SMc00074-C-XbaI-rev	ATATGTCGACTTTCCCTCGTATACCAGCCCA<br>ATATTCTAGAAGCCCGCTTCATCAGCGGAA	Construction of pK18mob2-*rgsP-egfp*
RHE_CH00976-C_Hind_f<br>RHE_CH00976-C_Xba_r	ATATAAGCTTCAGCTCGAAACCGATCTGCG<br>ATATTCTAGAGGCCCTTTTCGTCAGCGGAAA	Construction of pK18mob2-*rgsP*$_{\text{Re}}$-*egfp*
Atu0784-C_Sal_f<br>Atu0784-C_Xba_r	ATATGTCGACGCAAGGAGCTGTCCCTCGCCTAT<br>ATATTCTAGAAGACGCCCGCTTCGTCGG	Construction of pK18mob2-*rgsP*$_{\text{At}}$-*egfp*
SMc00074-C-KpnI-fwd<br>SMc00074-C-XbaI-rev	ATATGGTACCTTTCCCTCGTATACCAGCCCA<br>ATATTCTAGAAGCCCGCTTCATCAGCGGAA	Construction of pG18mob-*rgsP-3×flag*
SMc02432-C717-EcoRI-f<br>SMc02432-C738-XbaI-f	ATATGAATTCTTGGCAAGCAATGTCGACTT<br>ATATTCTAGACTTGCTTGCGACCTCGTT	Construction of pG18mob-*rgsM-3×flag*
SMc00074-C-SalI-fwd<br>mCh/egfp-Kpn-r<br>SMc00074-763-r-K_f<br>SMc00074-763-r-S_r	ATATGTCGACTTTCCCTCGTATACCAGCCCA<br>GTACGGTACCTTACTTGTACAGCTCGTCCATG<br>ATATGGTACCGGGCATCTGCCCCGTCAG<br>ATATCCCGGGATGGCGAAAAACGTGCTG	Construction of pK18mobsacB-*rgsP-egfp,* pK18mobsacB-*rgsP-mCherry* and pK18mobsacB-*rgsP-3×flag*
SMc02432-326-HindIII_fwd<br>SMc02432-326-PstI_rev	ATATAAGCTTGCGCGAGATCTCGATGCG<br>ATATCTGCAGACGTCTGATGGTTGGCGG	Construction of pK19mob2ΩHMB-*rgsM*
PT5-BamH-f<br>SMc00074-N584-Hind_r	ATATGGATCCCATATGCTTTCGTCTTCACCTC<br>ATATAAGCTTACAGGGTGAAGGCGTTGA	Construction of pK18mob2-P$_{lac\text{-}T5}$-*rgsP* (pWBT-*SMc00074* used as PCR template)
PhoAEc+79-f-Xba<br>PhoAEcstop-r-Kpn	GTGTTCTAGACCTGTTCTGGAAAACCGGG<br>TTCGGGTACCTTATTTCAGCCCCAGAGCGGC	Construction of pSRKKm-*rgsM-phoA,*

SMc02432-full-N-f	ATATCATATGAGCAGCGATAAGAACATGC	pSRKKm-*rgsM*$_{1-66}$-*phoA* and pSRKKm-*rgsM*$_{60-646}$-*phoA*
SMc02432-C738-XbaI-f	ATATTCTAGACTTGCTTGCGACCTCGTT	
SMc02432+198-r-Xba	GCCTCTAGACGGAATCGCCAGTTGCTGCCG	
SMc02432+178-f-Nde	GCCTCATATGCGGCAGCAACTGGCGATTCCG	
EGFP/mCh-f-Nde	GAGTCATATGGTGAGCAAGGGCGAG	Construction of pSRKGm-*mCherry,* pSRKGm-*mCherry-ftsZ*, pK18mobsacB-*mVenus-rgsM* and pK18mobsacB-*rgsM-3×flag*
EGFP/mCh-Xba-r-nostop	GTACTCTAGACTTGTACAGCTCGTCCATG	
mVenus_Kpn_fwd	ATATGGTACCATGGTGAGCAAGGGCGAGGAG	
SMc02432_-start_Xba_fwd	ATATTCTAGAAGCAGCGATAAGAACATGCTTC	
SMc02432_+750_Hind_rev	ATATAAGCTTCATGTTCTCCTCGACGACGC	
SMc02432-768-l-B_f	ATATGGATCCCGCTCGACTTGCATGTGTTAA	
SMc02432-768-l-K_r	ATATGGTACCGTTCTTATCGCTGCTCATGCAGG	
SMc02432-774-r-K_f	ATATGGTACCCCACACAGCAACGGTATCG	
SMc02432-774-r-S_r	ATATCCCGGGAGTACATGGAGAAGCATTCGGT	
FtsZ+1-Xba-f	GAATCTAGAATGGCCATCAACTTGCAGAAG	
FtsZ-end-Hind-r	CGCGAAGCTTTCAGCTCGACTGGCGGCGC	
N-Flag-mucR-XbaI_fwd	ATATTCTAGAACAGAGACTTCGCTCGGTACG	Construction of pK18mobsacB-*3×flag-mucR*
N-Flag-mucR-HindIII_rev	ATATAAGCTTTCACTTGCCGCGACGCTT	
N-FLAG-mucR-454-left-EcoRI_fwd	ATATGAATTCATCCGTGATAGATCAGCTTCCC	
N-FLAG-mucR-454-left-NdeI_rev	ATATCATATGTTCTTTCTCCTATCGGAATCCAG	
3xFLAG_Kpn-blnt_oligo1	CGATTACAAGGATCACGATGGTGATTACAAGGATCACGATAT CGATTACAAGGATGACGATGACAAGTGA	Construction of pABC-*rgsP-3×flag*, pR-*rgsP-3×flag*, pWBT-*rgsM-3×flag,* pK18mobsacB-*rgsP-3×flag,* pK18mobsacB-*rgsM-3×flag* and their derivatives
3xFLAG_Kpn-blnt_oligo2	TCACTTGTCATCGTCATCCTTGTAATCGATATCGTGATCCTTG TAATCACCATCGTGATCCTTGTAATCGGTAC	
FLAG_KpnI_rev	ATATGGTACCTCACTTGTCATCGTCATCCTTGTA	
SMc02432-C738-KpnI-r	ATATGGTACCCTTGCTTGCGACCTCGTT	
pUT18-Spe-f	GAACACTAGTATGACCATGATTACGCCAAG	Construction of pUT18Spe, pUT18Spe-*rgsP*$_{\Delta GGDEF\Delta EAL}$, pUT18C-*rgsM* and pKT25-*rgsM*
pUT18-Spe-r	CCTAACTAGTTTCCTGTGTGAAATTGTTATCC	
SMc00074-Xba_fwd	ATATTCTAGAATGCCCCTGACCCGTAAG	
SMc02432+1575-Kpn-+2-r	TATAGGTACCGTGATGTCGTTGATGGTCCCG	
SMc02432+3-Xba-f	TTCTCTAGAGAGCAGCGATAAGAACATGCTT	
SMc02432-end-r-Kpn	ATATGGTACCCTACTTGCTTGCGACCTCGTT	
SMc00074-seq-550	ACGCCTACAAGGATACCGTCA	Sequencing
SMc00074-seq-1146	ATCGTGCTCCTGATCGGC	
SMc00074-seq-1780	ACAATGTCCTCATCGCGCT	
SMc02432_679	TTCTATGTCGACCCGCAAC	
RHE_CH00976_seq-395	GAGCGAAGGCTTTGCGCT	
RHE_CH00976_seq-1141	CTGATCGGCTTCACCGTCAT	
RHE_CH03752_seq-361	AAGATGACGCTGGCCGCCAA	
Atu0784_seq-388	CAGCGCATCATCGCCATCA	
Atu0784_seq-1141	GTGCTGATCGTGCTGCTGAT	
Atu4178_seq-371	CTCACGTCAAGATTCCGCTG	
PCR1	CGGGCCTCTTCGCTATT	
PCR2	TTAGCTCACTCATTAGG	
egfp_rev	ACTTCAGGGTCAGCTTGCCGTA	

405	GATCCGGCAAACAAACCACC
456	CGCTCTCCTGAGTAGGACAAA
pQE_fwd	CGGATAACAATTTCACACAG
pQE_rev	CAAGCTAGCTTGGATTCTCACC
T25-r	CCTCTTCGCTATTACGCCAGC
T18-r	CGGAGACGGTCACAGCTTGTC
T18_fwd	AAAGCCTGTTCGACGATGG
T25_fwd	TGACGGCGGATATCGACATGT
